# Electron Transport through a Tryptophan Quadruplex in a Dimeric Azurin Construct

**DOI:** 10.1021/acs.jpcb.5c06932

**Published:** 2026-01-21

**Authors:** Martin Melčák, Jan Heyda, Filip Šebesta, Harry B. Gray, Stanislav Záliš, Antonín Vlček

**Affiliations:** † J. Heyrovský Institute of Physical Chemistry, Czech Academy of Sciences, Dolejškova 3, Prague CZ-182 23, Czech Republic; ‡ Department of Physical Chemistry, 534468 University of Chemistry and Technology Prague, Technická 5, Prague CZ-166 28, Czech Republic; § Department of Chemical Physics and Optics, Faculty of Mathematics and Physics, Charles University, Ke Karlovu 3, Prague CZ-121 16, Czech Republic; ∥ 6469 Beckman Institute, California Institute of Technology, Pasadena, California 91125, United States; ⊥ Department of Chemistry, 4617 Queen Mary University of London, London E1 4NS , U.K.

## Abstract

A tryptophan quadruplex at a protein–protein interface in a dimeric azurin construct mediates 8–11 ns intramolecular as well as interfacial electron hole transfer (HT) triggered by ultrafast photooxidation by a covalently attached organometallic chromophore (Takematsu et al. *J*. *Phys*. *Chem*. *B*., 2019, 123, 1578–1591). MM/MD and QM/MM/MD simulations characterized intermediates of through-quadruplex HT (i.e., states with one of the tryptophans oxidized) and assessed the feasibility of individual HT pathways. Simulations demonstrated that the oxidized quadruplex in aqueous solution occurs in four distinct states where the charge is predominantly (≥90%) localized at individual tryptophan indoles. Distributions of indole–indole distances, electronic couplings, as well as electrostatic potentials at indoles indicate kinetic and energetic preferences of interfacial over intramolecular ET. Interfacial indoles are tightly solvated by a chain of quasi-structural water molecules that are shielded from bulk water by protein folds. Solvating water molecules support ET by 0.1–0.2 Å shifts toward positively charged indoles. PDB search revealed that 4-Trp clusters are rather common among naturally occurring oxidoreductases.

## Graphical abstract

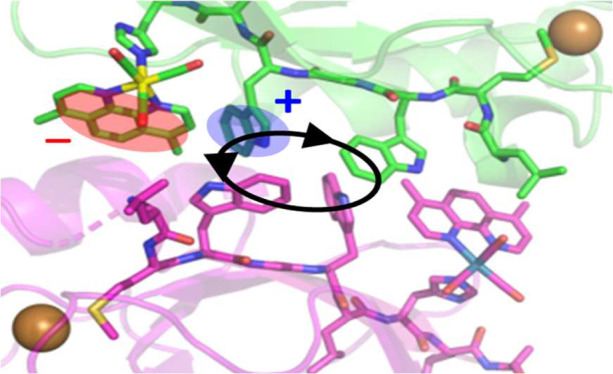

## Introduction

Tryptophan-containing mutants of the blue copper protein azurin provide a convenient platform to investigate multistep long-range electron transfer (ET) in folded polypeptide environments.^1–11^ In our work, a native Cu^I^ center was oxidized by sequential ET through one or two tryptophan residues to an electronically excited [Re^I^(His-azurin)­(CO)_3_(dmp)]^+^ (*Re, dmp = 4,7-Me_2_-phenanthroline), a chromophore bound to the protein through a histidine residue (His).^1–5^ **Re126W124W122Cu** ^**I**^ (Scheme 1) is a case in point.^4^ Optical excitation of [Re^I^(H126)­(CO)_3_(dmp)]^+^ prepares a Re­(CO)_3_ → dmp metal to ligand charge transfer (MLCT) excited state (*Re) that oxidizes the proximal tryptophan (W124) creating a charge-separated state, Re^I^(H126)­(CO)_3_(dmp^•–^)­(W124^•+^)­(W122)­Cu^I^, with multiexponential kinetics in the <1–450 ps range. A second ET step (W124^•+^ ← W122) produces a Re^I^(H126)­(CO)_3_(dmp^•–^)­(W124)­(W122^•+^)­Cu^I^ in ca. 8 ns. The ET sequence concludes by rate-determining (ca. 60 ns) W122^•+^←Cu^I^ ET forming Re^I^(H126)­(CO)_3_(dmp^•–^)­(W124)­(W122)­Cu^II^. Overall, the azurin Cu^I^ center is oxidized by 3-step *Re ← W124 ← W122 ← Cu^I^ ET over 22.9 Å in about 80 ns, which is roughly 10,000-times faster than estimated for single-step *Re←Cu^I^ electron tunneling over the same distance.^4^ The first two ET steps *Re ← W124 ← W122 take place between water-exposed residues at the protein surface. The second step (W124^•+^ ← W122 ET) can be described as a hole transfer (HT) W124^•+^ → W122.

**Scheme 1:**
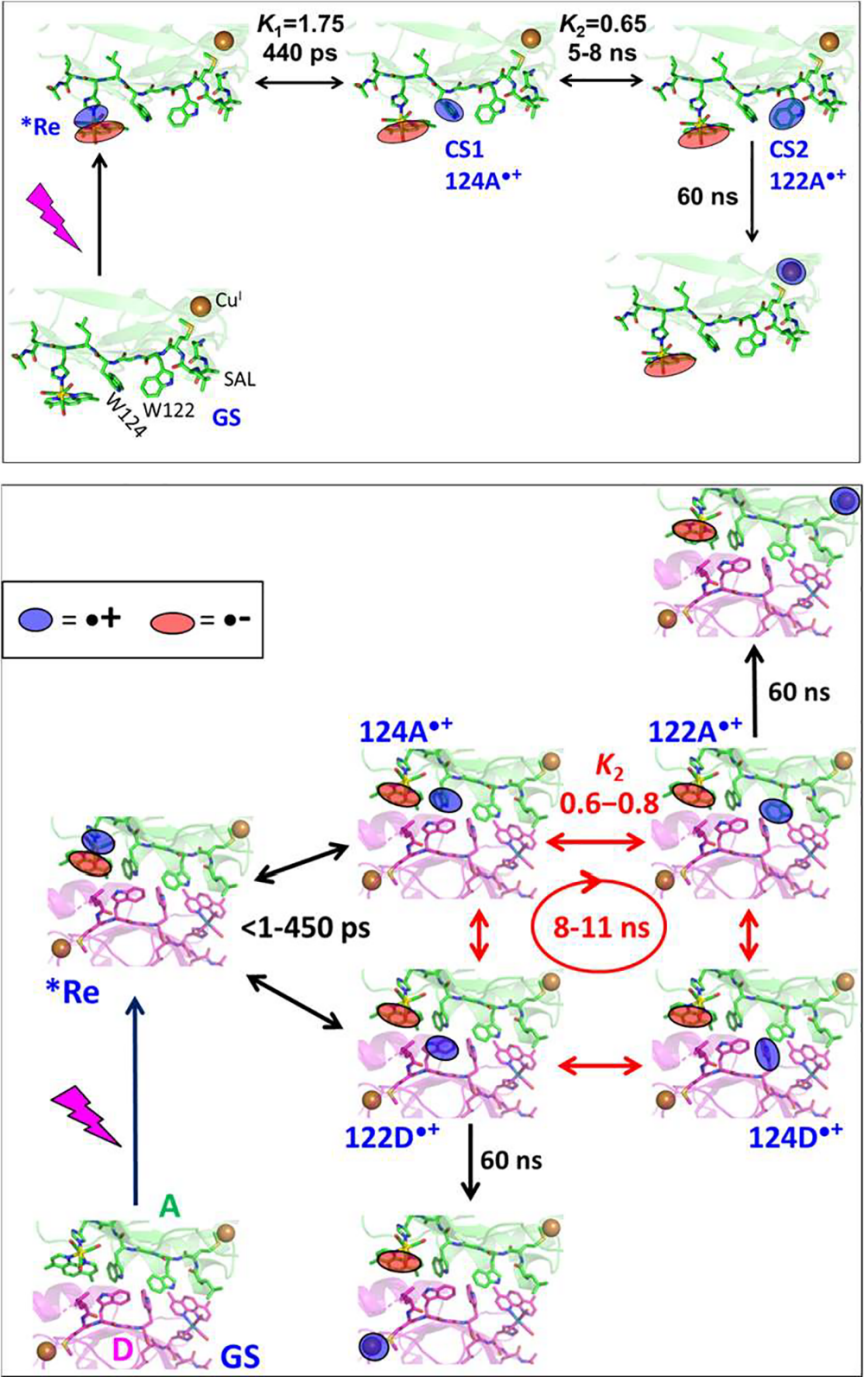
Hole Hopping through the **Re126W124W122Cu** ^**I**^ Monomer (Top) and Dimer **{Re126W124W122Cu** ^**I**^ **}**_**2**_ (Bottom, Showing the Interface between Molecules A (Green) and D (Purple) in the Crystal Structure PDB: 6MJS.^4,5^ ) Recombination and Back-ET Steps Are Omitted for Clarity)^a^

a Optically prepared *Re oxidizes one of the proximal tryptophans—124A in the monomer and either 124A or 122D in the dimer. Hole passage through the quadruplex occurs with an 8-11 ns lifetime. Oxidation of either one of the Cu^I^ centers (brown balls) follows. The present work analyzes hole hopping within the oxidized quadruplex (red arrows). The four possible states of the one-electron oxidized quadruplex are labelled by the position numbers of the oxidized tryptophan indole side-chain together with a letter A or D specifying the protein chain. In our previous work,^4,5,12,13^ the two states of the oxidized monomer were labelled CS1 and CS2, as shown in the top panel.

Theoretical analysis of W124^•+^ → W122 HT in **Re126W124W122Cu** ^**I**^ indicated^12^ that it is an adiabatic reaction controlled by environmental dynamics, whereby the hole transfer is accompanied by water molecules shifting in the same direction. Water fluctuations affect relative energies of the W124 and W122 sites driving W124^•+^ and W122 to degeneracy, and eventually enabling the HT. Simulations showed that, as W122 is partly shielded from the solvent by a nearby S118A119L120 segment (SAL, Scheme 1 top), concerted protein and water dynamics are required to initiate W124^•+^ → W122 HT. SAL-shielding increases the W122 formal potential relative to W124, making this step slightly endergonic and shifting the equilibrium toward W124^•+^, which, in conjunction with competing decay to the ground state through Re^I^(dmp^•–^)/W124^•+^ recombination, diminishes the overall Cu^I^ oxidation yield.^4,12^

Solvent exposure of the Re–W124–W122 system changes profoundly at higher concentrations (>200 μM) when **Re126W124W122Cu** ^**I**^ dimerizes.^5,14^ Examination of the crystal structure revealed a protein–protein interface (|) where the two intramolecular HT pathways interact through dmp ligands and the four indoles (Figure 1).^5^ The interface consists of two nearly coplanar units (dmpA|122D and dmpD|122A) and a tryptophan quadruplex {124A,122A|122D,124D}, where the four indoles are T-oriented relative to each other. Intramolecular and interfacial distances between cofactors are relatively short (Figure 1). Optical excitation of **Re** at one of the protein chains in **{Re126W124W122Cu** ^**I**^ **}**_**2**_ leads to Cu^I^ oxidation with a rate comparable to that of the monomer (Scheme 1 bottom). The process starts with ultrafast oxidation of the quadruplex. Hole transport through the oxidized quadruplex is an 8–11 ns process, followed by ∼60 ns ET from either of the two Cu^I^ sites. The parallel occurrence of intramolecular and interfacial ET was demonstrated by vastly different rates of back ET from dmpA^•–^ to the two Cu^II^ centers produced.^5^

**Figure 1:**
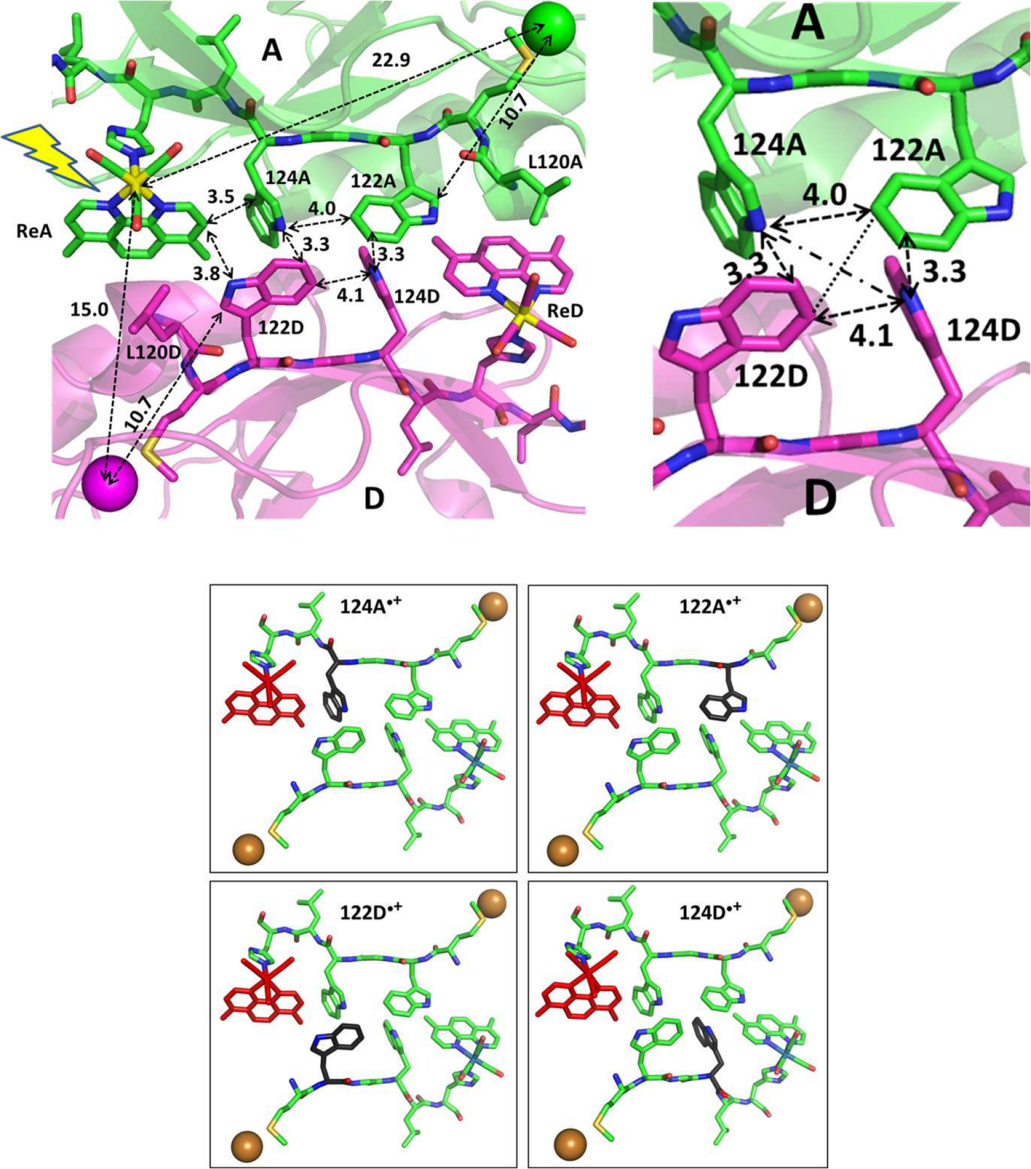
Top left: Structure of the interface between molecules A and D in the asymmetric unit of {Re126W124W122Cu^I^}_2_ (PDB: 6MJS). Right: Detail of the quadruplex structure. ET-relevant shortest distances (Å) are shown by dashed black arrows. Diagonal distances: 3.7 Å (122D–122A, dotted); 5.4 Å (124A–124D, dash-dot). (All distances were measured between C or N atoms, disregarding hydrogens.) Bottom: four states of the oxidized quadruplex simulated in this work. Red: Re^I^(CO)_3_(dmpA^•–^) (ReA^–^). Black: cationic tryptophan residues.

In this work, we have focused on the molecular and electronic structures of the one-electron oxidized tryptophan quadruplex and, namely, on its hole-transporting behavior. Experimentally, we know that there are two entry- and two exit sites (Scheme 1) and that hole transport through the quadruplex occurs in a kinetically distinct 8–11 ns step.^5^ Hole passage through the quadruplex could involve structural changes, hole transfer between the tryptophan indoles, as well as its localization (delocalization) at (over) particular sites. Since such steps were not distinguished experimentally, we have resorted to Born–Oppenheimer QM/MM/MD simulations of **{Re126W124W122Cu** ^**I**^ **}**_**2**_ in aqueous solution to identify the most likely structural rearrangements and HT steps taking place in the tryptophan quadruplex upon its oxidation and to assess its propensity in mediating interprotein ET. In particular, we have modeled the behavior of the structurally well characterized interface between molecules A and D in the crystal structure PDB: 6MJS (Figure 1). The ET-active tryptophan indole groups are labeled by their corresponding position numbers plus a letter A or D specifying the protein molecule. Assuming predominant hole localization, the oxidized quadruplex could occur in four states schematically shown in Scheme 1 and Figure 1-bottom. The simulated processes were started by “instantaneous” hole injection from *Re on chain A (shown in the structures at top-left) that oxidized either 124A or 122D. Final Cu^I^ oxidation occurs either from 122A^•+^ or 122D^•+^.

The work started with classical simulations of **{Re126W124W122Cu** ^**I**^ **}**_**2**_ in the four oxidized quadruplex states, which revealed distributions of their structures and solvation. Then, we proceeded to calculating quantum mechanical (QM/MM/MD) charge and spin trajectories that confirmed predominant hole localization at individual indoles and provided background for analyzing hole transfer. In particular, electronic coupling pointed to kinetically favorable hopping pathways, while differences in electrostatic potentials exerted by the environment on indoles together with classically estimated free energy changes indicated their energetics. Finally, we discovered that tryptophan clusters are relatively common among naturally occurring oxidoreductases, likely with predominantly structural functions. The present azurin construct represents an unusual case where an interfacial tryptophan quadruplex mediates electronic interactions and supports hole hopping between protein molecules.

## Computational Approach

Simulations were performed on **{Re126W124W122Cu** ^**I**^ **}**_**2**_ solvated by 38289 water molecules and 6 Na^+^ ions for charge compensation. Both H35 and H35′ were neutral (deprotonated). The simulation procedure is outlined in Figure 2. MM/MD simulations were performed in the presumed order of ET steps, going from GS to MLCT, from which they proceeded to 124A^•+^ and 122D^•+^, and then to 122A^•+^ and 124D^•+^, respectively. Each state was described by a unique set of force-field parameters (SI-1, Section S9.1) that we have developed earlier.^12,13^ A 20 ns GS trajectory was calculated first, starting from the dimeric unit of molecules A and D in the PDB structure 6MJS. (Note that A/D is a crystallographical notation bearing no relation to an acceptor/donor behavior.) In the next step, parametrization of Re­(H126)­(CO)_3_(dmpA)^+^ (**ReA**) was switched to that of the MLCT state and four 1 ns simulations of the ^3^MLCT state were run, starting from snapshots of the GS trajectory at 5, 10, 15, and 20 ns. The end structures of MLCT trajectories then served as starting points for one 10 ns classical simulation of the 124A^•+^ state and one classical simulation of 122D^•+^. Next, 10 ns 122A^•+^ and 124D^•+^ simulations were run, starting at 1, 4, and 10 ns of 124A^•+^ and 122D^•+^ trajectories, respectively. Classical MM/MD simulations of each state were complemented by 500 fs long quantum-mechanical (QM/MD) simulations that started at different MM/MD times (every 100 or 50 ps, depending on the simulated state), employing the CAM-B3LYP functional. In general, MM/MD simulations provided distributions of molecular structural and solvation parameters. QM/MD trajectories of Mulliken charges and spin densities at individual cofactors (Re­(CO)_3_, dmp, and all four tryptophans) revealed hole localization in each CS state, electronic couplings between them, as well as electrostatic potentials at individual indoles exerted by protein and water molecules.

**Figure 2:**
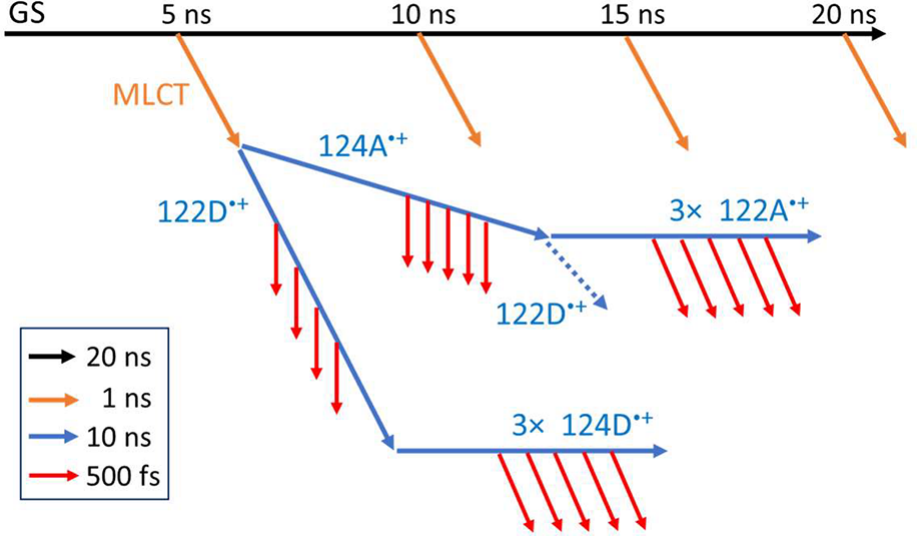
Visualization of the QM/MM/MD procedure showing the sequence of the simulation steps. Black, brown, and blue arrows depict classical trajectories. Thirty QM/MM/MD simulations (indicated in red) of each oxidized state started at various points of corresponding MM/MD trajectories. Dotted arrow: An alternative MM/MD 122D^•+^ simulation started from 124A^•+^ instead of MLCT.

## Results

### Structures

MM/MD trajectories of shortest indole–indole and indole-dmp distances are shown in Figures S1–S13. Distributions of ET-relevant distances are displayed in Figure S14 while Figures 3 and S15–S17 correlate various ET-relevant distances with the 124A–122A distance in the four quadruplex oxidized states. The GS simulation revealed that the neutral quadruplex changes shape from near-rhombic in a crystal to trapezoidal in solution, the 124A–122A “side” becoming shorter than 122D–124D whose lengths were more broadly distributed. Interfacial separations (edges) 124A–122D and 122A–124D as well as the 122D–122A diagonal remained short, whereas the 124A–124D diagonal lengthened (Figure S14 top row). ^3^MLCT excitation of **Re** kept the structure virtually unchanged. *Re­(H126)­(CO)_3_(dmpA)^+^ (*ReA) was oriented so that the dmpA ligand pointed toward 124A, resulting in a narrow distribution of short dmpA-124A distances. Interfacial dmpA-122D distances were slightly longer and more broadly distributed. Simulated ^3^MLCT structures were compatible with intramolecular as well as interfacial oxidation of 124A and 122D, respectively.

**Figure 3:**
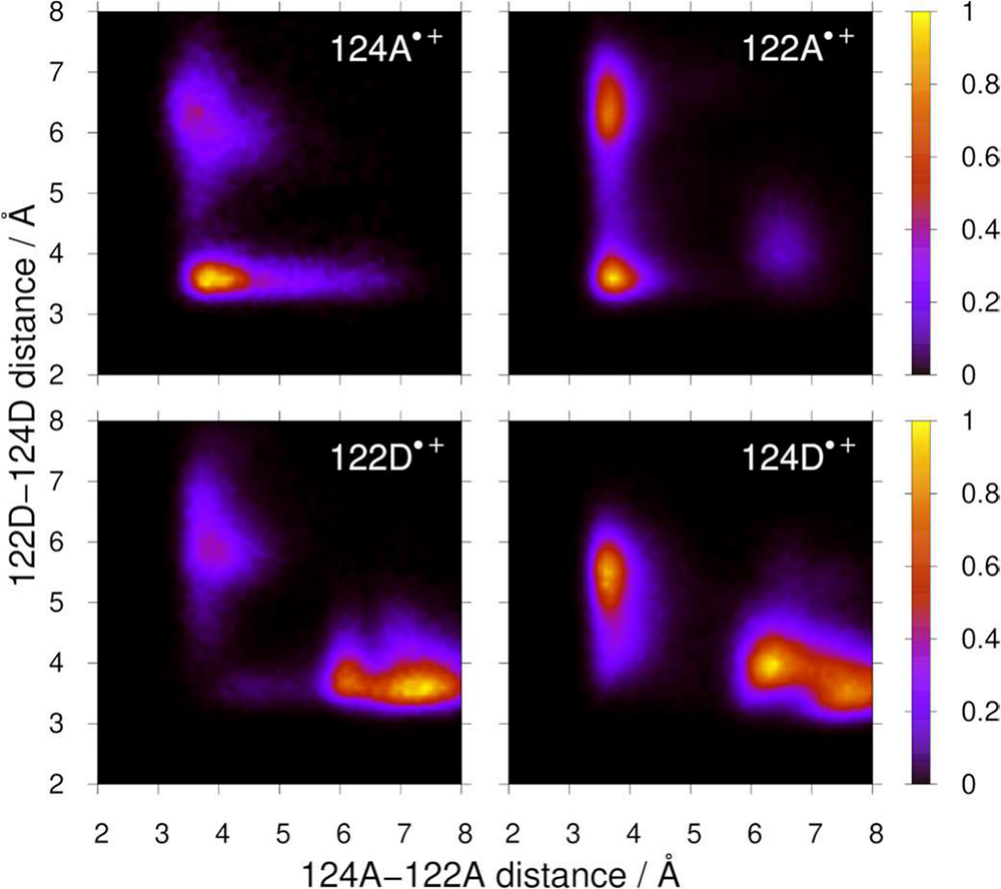
2D structural maps correlating 124A–122A and 122D–124D shortest distances in the four oxidized quadruplex states for structures with “*in*” ReA^–^ orientations. More correlations are in Figures S15–S17.

The 124A^•+^ state occurred in two forms with short and long dmpA–124A distances whose distributions peaked between 3–4 and 5–6 Å, respectively. These two structural forms correspond to “*in*” and “*out*” conformers analogous to those found in MM/MD simulations of the monomeric state Re^I^(H126)­(CO)_3_(dmp^•–^)­(W124^•+^)­(W122)­Cu^I^ where only the “*in*” conformer underwent 124^•+^ → 122 HT.^12^ The *in*-124A^•+^ form of the dimer exhibited two main quadruplex conformers: an essentially rhombic one with both 124A–122A and 122D–124D distances short, and a short 124A–122A/long 122D–124D trapezoid. In addition, a very small subpopulation of long/short structures emerged from the long tail of the 124A–122A distance distribution. The interfacial edges (124A–122D and 122A–124D) and the 122D–122A diagonal were short in all forms. This structural pattern was largely preserved in the 122A^•+^ state, only with a more prominent short 124A–122A/long 122D–124D trapezoidal subpopulation (Figure S16).

The 122D^•+^ state can be formed either directly from MLCT (by *ReA ← 122D ET) or from the 124A^•+^ state (124A^•+^ → 122D HT). MM/MD simulations starting from end-points of MLCT and 124A^•+^-state trajectories yielded similar results, except for the presence of a small population of “*out*” conformations in 124A^•+^-started simulations (Figure S18). The majority population was trapezoidal with long 124A–122A and short 122D–124D distances (Figure S15), owing to flipping the intramolecular distances upon transferring the hole to chain D, which occurred within 1–2 ns (Figure S6). The “edges” stayed short in most structures, as did the 122D-122A diagonal. Minor contributions were made by conformations with short 124A–122A and long 122D–124D diagonal (Figure S15).

The 124D^•+^ state occurred in several forms of comparable abundance: two subpopulations of a flipped trapezoid (long 124A–122A/short 122D–124D) and short/long trapezoids, each with different combinations of edge and diagonal distances (Figure S17).

In summary, MM/MD simulations indicated that the interfacial tryptophan quadruplex in aqueous solution is a stable structural motif, noncovalently linking two protein molecules in neutral as well as monocationic states. The one-electron oxidized quadruplex was structurally more heterogeneous than the neutral GS, occurring in a mixture of rhombic (only 124A^•+^ and 122A^•+^ states) and at least two trapezoidal (all states) forms. The lengths of the 124A–122A and 122D–124D “sides” varied relative to each other, being comparable and short in 124A^•+^ and 122A^•+^ rhomboids. Hole transfer to molecule D was often accompanied by 122D–124D shortening. The two interfacial indole separations (edges) and the 122D–122A diagonal were short in all states most of the time. The 124D^•+^ state was the most structurally heterogeneous. Having established structural distributions of the oxidized quadruplex, we proceed to examine hydration of the indole side chains.

### Solvation

The structural heterogeneity of the oxidized quadruplex translated to even larger solvational heterogeneity. Figure S19 shows overlays of water radial distribution functions (rdf) around indole NH groups in the four oxidized states obtained from all calculated MM/MD trajectories. Their large spread disfavored developing a meaningful ensemble-average solvation model, although most (but not all) trajectories indicated greater solvation of the oxidized indole in each state, owing to its positive charge. The huge solvational heterogeneity mostly arose from greatly variable relative positions and distances between S118A119L120 segments of each protein chain (SAL­(A), SAL­(D)) and the indoles (Figure S20). Similarly to the monomer,^12^ water access to 122A (and 122D) depended on the distance between their N–H groups and the closest SAL backbone oxygen atom, which effectively picked between N–H···OH_2_ and N–H···O­(SAL) hydrogen bonding.

In order to understand solvation changes accompanying 124A^•+^ → 122A and 124A^•+^ → 122D HT, we reduced the structural heterogeneity by selecting MM/MD trajectories of *in*-124A^•+^ conformers whose starting structures had 124A–122A and 122D–124D shortest distances smaller than 4 Å and SAL­(A)–122A and SAL­(D)–122D shortest distances longer than 3.5 Å, corresponding to an *in*-124A^•+^ subpopulation of nearly rhombic structures where 122A and 122D solvation was permitted by reasonably long distances to SAL. In each of these trajectories, indole water coordination numbers and 3D structural maps of the 124A^•+^ state were calculated over 200 ps, after which the charge distribution was switched either to 122A^•+^ or 122D^•+^, which amounted to forced HT. In these states, simulations continued for another 200 ps. Ground-state water coordination numbers were obtained separately from GS trajectories with 124A–122A distances <4 Å. Average quadruplex molecular structures in the oxidized states were superimposable (Figure S21), so that changes in average water positions and coordination numbers can be attributed to movements of water molecules relative to a virtually rigid quadruplex (whose geometry ensembles fluctuated comparably in all states). Only a limited number of water molecules was found in the sterically restricted quadruplex region, which essentially kept their ground-state positions on going from GS to 124A^•+^ and then switching to 122A^•+^ or 122D^•+^. Upon HT, only the closest water molecules shifted by 0.1–0.2 Å toward the oxidized indole and its NH group, and away from the indole that was reduced (Figures 4 and S22). Hydration of the quadruplex region appeared as an organized framework of quasi-structural water molecules interacting with each other as well as C=O and NH groups of the quadruplex and SAL. Their movement and exchange with the bulk water reservoir were restricted by the protein fold.

**Figure 4:**
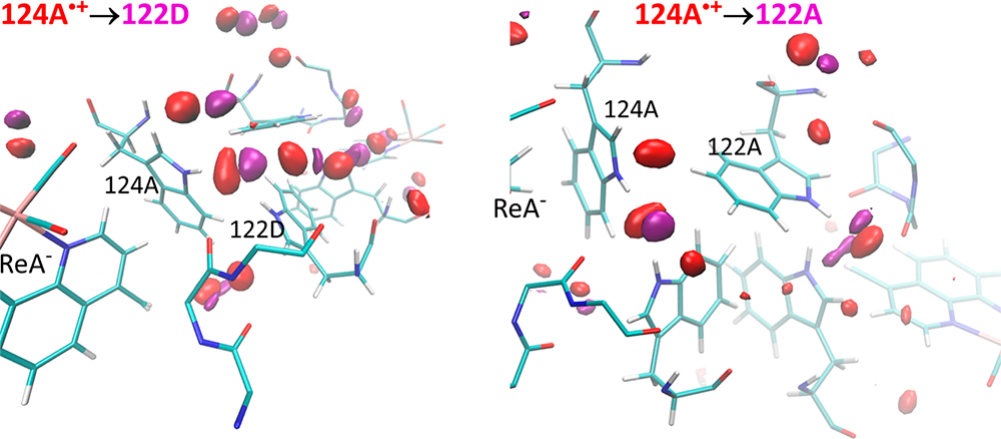
Spatially resolved hydration shifts upon 124A^•+^ → 122D (left) and 124A^•+^ → 122A (right) hole transfers. Difference 3D spatial density maps of water oxygen atoms (Δρ = ρ_final_ – ρ_124A•+_) superimposed on the average molecular structure of tryptophan quadruplex side chains. Regions of excess hydration in the 124A^•+^ state are shown at the density isocontour Δρ = −2.5 × ρ_0_ (red), and regions of excess hydration in the final states (122D^•+^, 122A^•+^) at Δρ = +2.5 × ρ_0_ (violet) of the bulk water density (ρ_0_). Water oxygen maps are shown within 4 Å from NH groups at 0.1 Å spatial resolution. Density differences were calculated from maps in Figure S22D. Alternative representations are shown in Figure S22.

Solvation changes accompanying 124A^•+^ → 122A and 124A^•+^ → 122D HT followed different mechanisms: 124A^•+^ → 122A involved a water molecule shift from the region between SAL­(A), dmpD and 124D toward 122A, while loss of charge on 124A resulted in another water molecule shifting away from 124A-NH. On the other hand, 124A^•+^ → 122D HT involved a single water molecule shift from 124A-NH toward 122D-NH. Another water molecule moved away from the 124A aromatic region (Figures 4 and S22, also manifested by a drop in 124A water coordination number, Figure 5).

**Figure 5:**
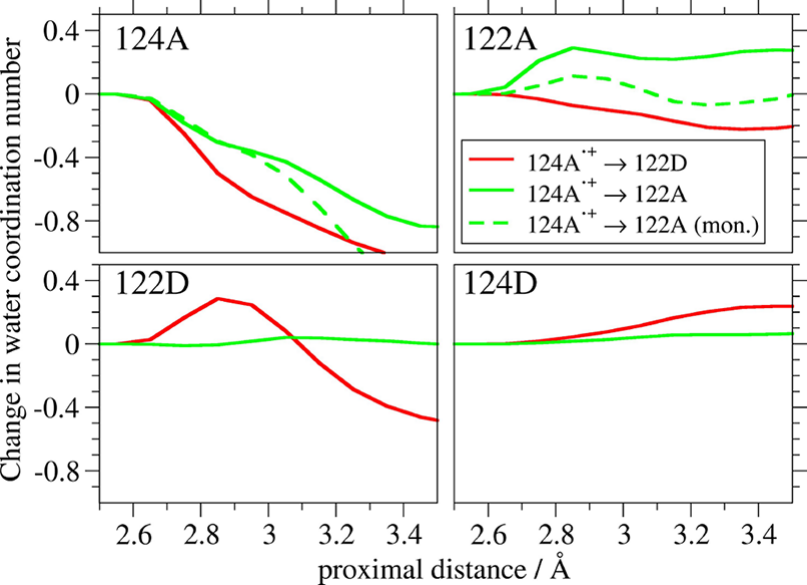
Changes of water coordination numbers of the four indoles upon 124A^•+^ → 122D (red) and 124A^•+^ → 122A (green) HT in the dimer and monomer (dashed green). (Defined as the final state minus 124A^•+^). See the text for trajectories selection. Coordination numbers are exclusive, i.e., each molecule is assigned to the single closest indole. Changes of NH coordination numbers and their absolute values are displayed in Figures S23–S25.

Changes of water coordination numbers of indoles and their NH groups are shown in Figures 5 and S23–S25. In accord with 3D mapping of water movements (Figures 4 and S22), 124A^•+^ → 122D interfacial HT was accompanied by an increase (decrease) of 122D (124A) water coordination numbers due to a 124A-NH - bound water moving near 122D-NH. This movement was accompanied by a smaller water shift on the other side of the quadruplex from 122A to 124D. Longer-range 122D and especially 124A solvation decreased as water moved away from their aromatic regions, also demonstrated by overall quadruplex dehydration within the 3.5 Å range (Figure S26). Intramolecular 124A^•+^ → 122A HT involved increasing short-range 122A-NH hydration as well as hydration of the 122A indole aromatic region at longer distances. 124A water coordination numbers decreased concomitantly, albeit less than upon 124A^•+^ → 122D. Changes around 122D and 124D were negligible. The overall quadruplex desolvation, which was ca. 1/3 of that observed upon 124A^•+^ → 122D HT, occurred mostly at 124A (compare Figures S26 and 5).

Comparing hydration changes of 122A upon 124A^•+^ → 122A HT (green curves in Figures 5 and S23) with those of 122D upon 124A^•+^ → 122D HT (red curves) indicated that water had a better access to the produced 122A^•+^ than 122D^•+^ indoles at distances longer than ca. 3 Å. In fact, 122D^•+^ experienced dehydration of its aromatic region. The better water access to 122A than 122D at longer distances accords with the different mechanisms of solvent response to 124A^•+^ → 122A and 124A^•+^ → 122D HT discussed above. It likely is attributable to different 122A and 122D environments containing negatively charged dmpA^–^ (**ReA** ^**–**^) and positively charged **ReD**, respectively.

Water coordination numbers of 124A and 122A indoles also were calculated for the two oxidized states of the **Re126W124W122Cu** ^**I**^ monomer (dashed curves in Figures 5 and S23–S26). Despite occurring at the protein surface, short-range indole and NH hydration in either monomer state was similar (for 124A^•+^ up to 2.9 Å) or smaller (for 122A^•+^ up to 3.15 Å; up to at least 3.5 Å for 122A^•+^-NH) than in the dimer (Figures S24 and S25). Lower 122A^•+^ hydration in the monomer is attributable to tighter SAL­(A) binding. Fewer structural water molecules occurred in the immediate vicinity of 124A···122A at the monomer surface, but they were connected to bulk water molecules, leading to more rapid increase of water coordination numbers of both indoles at longer distances than in the dimer (depicted in Figure S28a,b visualizing water molecules at 3.5, 4.0, and 5.0 Å from the indoles).

Upon 124A^•+^ → 122A HT, overall 122A^•+^ as well as 122A^•+^-NH short-range hydration increased less in the monomer than in the dimer. Above ca. 3 Å, 122A^•+^-NH monomer hydration rise overtook that of the dimer, whereas overall monomer 122A^•+^ hydration dropped to the level of neutral 122A whereas that of the dimer stayed higher. It appeared that above 3 Å the 122A^•+^ aromatic region stayed better solvated in the dimer, whereas the NH group was more solvated in the monomer. 3D maps of water distribution in the monomer were consistent with a water molecule moving away from 124A upon 124A^•+^ → 122A HT into bulk water (Figure S29). A small coincident rise of 122A hydration originated mainly from a shift and localization of a water molecule placed between 122A and SAL­(A). This behavior was similar to that described above for 124A^•+^ → 122A HT in the dimer.

In summary, tryptophan quadruplex solvation was found to be heterogeneous. The oxidized indole in each state is more strongly solvated than neutral ones. Indoles are solvated by a short chain of water molecules largely disconnected from the bulk. 124A^•+^ → 122D and 124A^•+^ → 122A HT are accompanied by increasing solvation of the oxidized indole and 124A^•+^ desolvation but the underlying mechanisms are different: a direct water-molecule shift from 124A^•+^ to 122D upon 124A^•+^ → 122D HT; and water shifting toward 122A from its broader surroundings while a different water molecule moves away from the aromatic 124A region in the case of 124A^•+^ → 122A.

### Hole Localization

Charge and spin-density distributions were examined by extending MM/MD trajectories with QM/MM/MD simulations, typically for 500 fs. The quantum part consisted of **Re** ^**–**^ on the protein chain A (**ReA** ^**–**^) and the whole tryptophan quadruplex (including G123 that link tryptophans in each protein). The rest of the system including water molecules was described by MM. QM calculations employed the CAM-B3LYP range-separated functional, which is suitable for describing aromatic indole groups interacting over a broad range of distances. For some trajectories, we switched the functional after 500 fs to PBE0 and continued the simulation for another 200–500 fs.

Charges (spin densities) were narrowly distributed in each state around ca. +0.9 (1.0) at the oxidized indole and close to 0 at the other three (Figure 6). Inspection of individual CAM-B3LYP QM/MM/MD trajectories (Figures S30–S33) revealed occasional short periods of partial interfacial indole–indole delocalization along the quadruplex edges and along the short diagonal 122D-122A in the 122D^•+^ and 122A^•+^ states.

**Figure 6:**
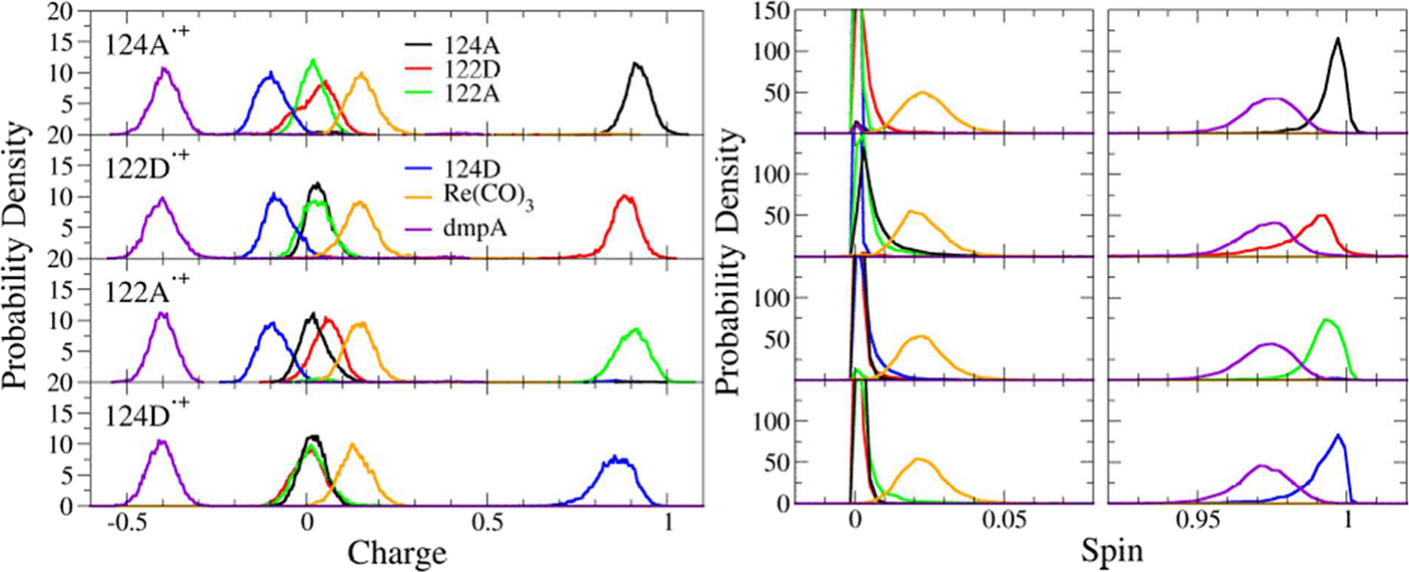
Mulliken charge (left) and spin-density (right) distributions at the four indoles, dmpA, and Re­(CO)_3_.

In particular, 124A^•+^ was fully charge-localized (Figure S30), whereas 122D^•+^ showed short periods of partial delocalization within 122D^•+^–124A and 122D^•+^–122A indole pairs (Figure S31). 122A^•+^ was localized most of the time, although a few trajectories showed brief edge delocalizations to 124D that in one case resulted in a complete hole transfer (Figure S32). One trajectory showed a short period of intramolecular 122A^•+^–124A delocalization that was followed by 122A^•+^ → 124A back-HT ca. 30 fs later; and another trajectory indicated minor diagonal delocalization with 122D. 124D^•+^ was predominantly localized with some trajectories exhibiting occasional hole delocalization along the edge to 122A (Figure S33).

The dmpA ligand had a ca. −0.4 charge in all states. Re­(CO)_3_ was slightly positive (ca. +0.2), in accord with the Re^I^(H126)­(CO)_3_(dmpA^•–^) formulation of **ReA** ^**–**^. Spins on dmpA (and Re­(CO)_3_) were smaller than 1 (and slightly above 0). Systematic anticorrelation of small charge- and spin-density fluctuations arose from variable mixing between dπ­(Re) and π*­(dmpA) orbitals.

Switching the functional from CAM-B3LYP to PBE0 at the end of QM/MM/MD simulations preserved the predominantly hole-localized character of all four states, but partial delocalization between cationic and neutral indoles increased. Charges (and spins) on oxidized indoles were slightly lower in PBE0 than CAM-B3LYP trajectories and their fluctuations anticorrelated with those at neutral indoles lying opposite along interfacial edges (compare Figures S30–S33 with S34–S37) and occasionally along the short diagonal or the quadruplex sides.

Overall, QM/MM/MD charge and spin distributions supported the predominantly hole-localized character of all four oxidized quadruplex states (Figure 1). Partial hole delocalization occurred rarely for short periods along the edges and the short diagonal within indole pairs. There was no evidence for hole delocalization over the whole quadruplex. The hole-localized electronic structure supports treating hole transport through the tryptophan quadruplex as a sequence of one or two hole-transfer steps between indole side chains. To assess their energetic feasibility, we examined electrostatic potentials at individual indoles in the four oxidized quadruplex states, obtained from QM/MM/MD simulations.

### Electrostatic Potentials and Reaction Free Energies

The environment (protein, **ReA** ^**–**^, **ReD**, solvent and counterions) creates an electrostatic potential at the indoles (ϕ­(indole)). A more negative potential at a given indole favors hole localization at that site. Potential distributions at the oxidized indole in different states partly overlapped while 122D^•+^ and 122A^•+^ subpopulations experienced more negative potentials than 124A^•+^, and even more than 124D^•+^ (Figure 7 top left), suggesting that electrostatic energies of the oxidized states increase in the order 122D^•+^ ≤ 122A^•+^ < 124A^•+^ < 124D^•+^. Partitioning the total potential to protein (including **ReA** ^**–**^ and **ReD**) and solvent (H_2_O + Na^+^) contributions (Figures 7, S38, and S39) showed that 124A^•+^ and 122D^•+^ were predominantly stabilized by protein, and 122A^•+^ and 124D^•+^ by solvent. (Different solvent and protein electrostatic effects on 122A^•+^ and 122D^•+^ accord with solvation differences discussed in the previous section, namely lower 122D^•+^ water coordination than 122A^•+^).

**Figure 7:**
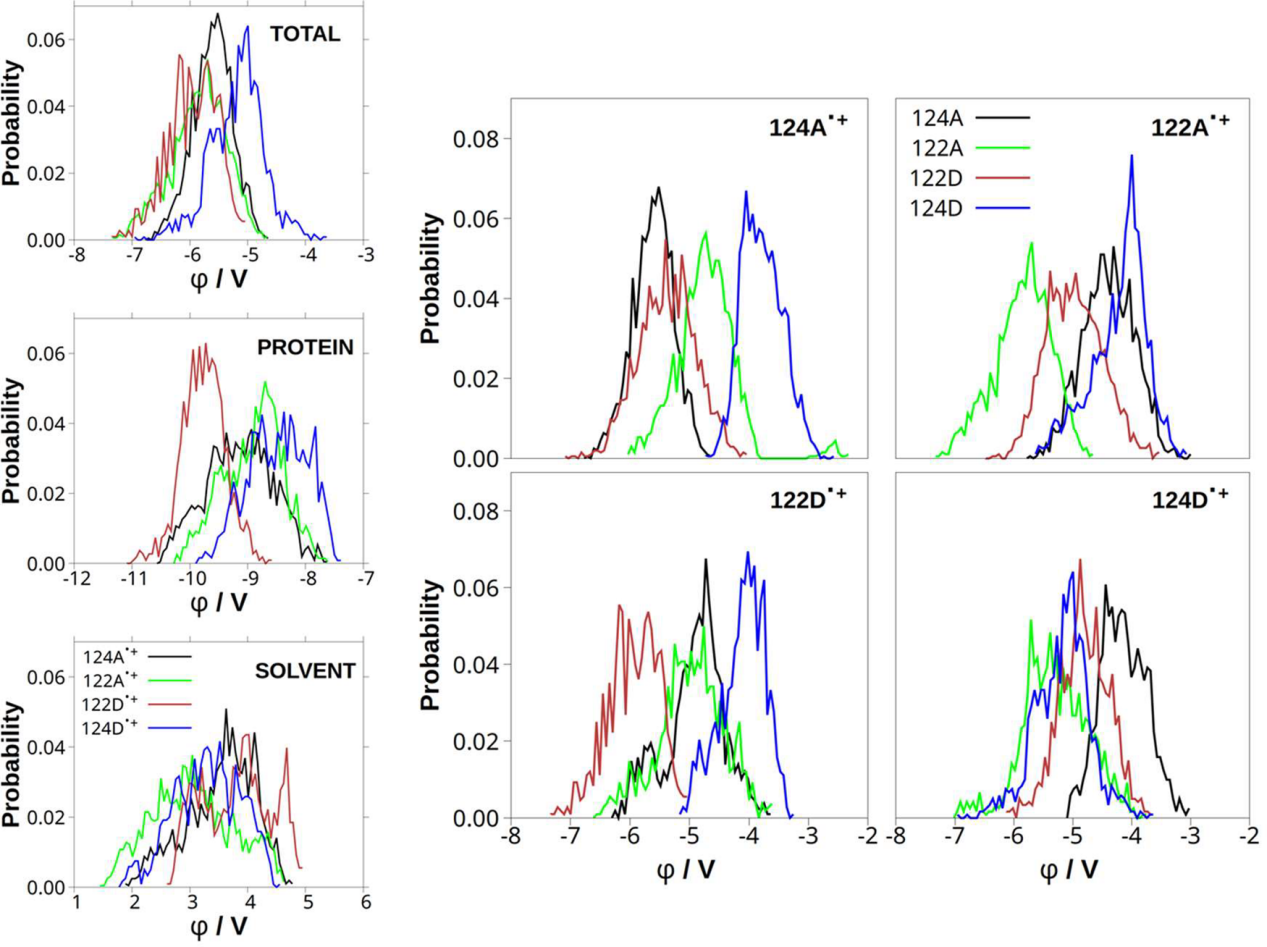
Left column: Distributions of total electrostatic potentials at oxidized indoles (top) and their contributions from protein (middle) and solvent (H_2_O+Na^+^, bottom). Right: distributions of total potentials (generated by solvent and protein except the oxidized indole) at individual indoles in each state. Potentials were calculated from CAM-B3LYP QM/MM/MD trajectories after the first 300 fs of equilibration.

Potentials at neutral indoles were calculated in two ways, either including (Figure S38) or excluding (Figures 7-right, 8, and S39) the oxidized indole from protein. Comparison of Figures S38 and S39 shows that the potential generated by the oxidized indole increased potentials at the other three indoles but did not really discriminate among them. Further separation of the protein environment revealed a strong destabilizing effect of the **ReD** positive charge (the dmpD ligand) that increased potentials at 124D and, less, 122A in all states. On the other hand, the negative charge on the **ReA** ^**–**^ (dmpA^•–^) negatively shifted potentials at 124A and 122D relative to those at the other two indoles (Figure S40). The SAL segment, Q107, and M109 of either protein chain had negligible effects on electrostatic potentials at the indoles.

**Figure 8:**
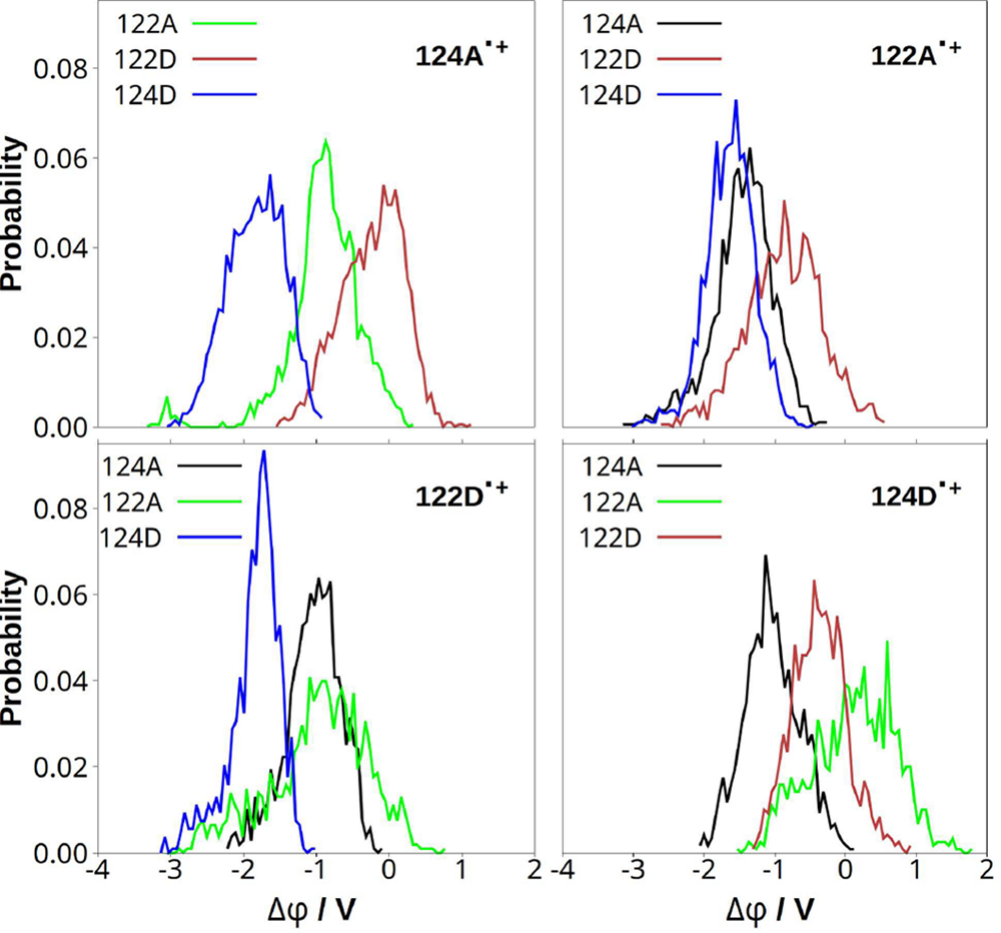
Distributions of differences (Δϕ) between the oxidized indole potentials and those of three neutral indoles in each state. Potentials generated by the whole system except the oxidized indole were calculated from CAM-B3LYP QM/MM/MD trajectories after the first 300 fs of equilibration.

The difference between electrostatic potentials at oxidized and neutral indoles (Δϕ = ϕ­(indole^•+^) – ϕ­(indole_*i*_)) indicates the thermodynamic feasibility of indole^•+^ → indole_*i*_ HT (the subscript *i* denotes any of the three neutral indoles) in a given state. Higher Δϕ values correspond to a larger ET driving force. The most positive Δϕ values were found (Figure 8) for 122D in 124A^•+^ and 122A^•+^ states; and for 122A in the 124D^•+^ state, favoring 124A^•+^ → 122D, 122A^•+^ → 122D, and 124D^•+^ → 122A HT steps. 124A^•+^ → 122A and 124D^•+^ → 122D HT also appeared feasible. These qualitative estimates based on electrostatic potentials (obtained from QM/MM/MD simulations) accord with reaction free energies (Δ*G*) obtained from all classical (MM/MD) trajectories (summarized in Table 1).

**Table 1:** Reaction Free Energies (Δ*G*, meV) of Individual HT Steps Obtained from All MM/MD Trajectories^a^

	to 124A	to 122A	to 122D	to 124D
from 124A^•+^		+67	–320	+172
from 122A^•+^	–67		–296	+18
from 122D^•+^	+320	+296		+262
from 124D^•+^	–172	–18	–262	

a Calculated according to ref 15. Reported Δ*G* values refer to the whole ensemble. The large structural/solvational heterogenity leads to a large spread of values over various subpopulations, as indicated in Table S1.

### Electronic Coupling

Calculated electronic couplings medians (*H*_ab_, Table 2) among the four states (approximated as indole-localized diabatic states) were the highest for HT along the short diagonal (122D^•+^ → 122A), followed by edges (124D^•+^ → 122A, 124A^•+^ → 122D), and then intramolecularly along the sides (124A^•+^ → 122A, 122D^•+^ → 124D). *H*_ab_ values in opposite directions were smaller but still sufficient for HT. The dependence of *H*_ab_ on HT direction arose from different structure and solvation distributions in the four states. *H*_ab_ values were in each case broadly and asymmetrically distributed, tailing toward larger values (Figures S41–S44) and producing large differences between median and average values (Table S2).

**Table 2:** Median Electronic Coupling (*H*_ab_) Values (meV) for Hole Transfer from Oxidized Indoles Specified in the First Column to Neutral Indoles in the First Row

	to 124A	to 122A	to 122D	to 124D	to **ReA** ^**–**^
from 124A^•+^		5	31	0.2	10
from 122A^•+^	14		11	18	0
from 122D^•+^	25	41		7	0
from 124D^•+^	0.1	31	8		

## Discussion

Experimental multistep ET kinetics of **{Re126W124W122Cu** ^**I**^ **}**_**2**_ are comparable to those of the monomer (Scheme 1): Tryptophan oxidation by optically excited *Re is multiexponential with a principal 400–500 ps component, followed by 8–11 ns hole hopping through the tryptophan quadruplex, and ∼60 ns Cu^I^ oxidation by 122A^•+^ or 122D^•+^. The occurrence of both intramolecular and interfacial processes was confirmed by kinetically distinguishing back-ET in the corresponding Cu^II^ reaction products.^4,5^

Our previous QM/MM/MD simulations of 124A^•+^ → 122A HT in the monomer^12^ showed that it is an adiabatic process driven by increasing 122A solvation in the course of structural fluctuations that shifted 122A toward 124A^•+^ and away from SAL­(A), creating an opening for the incoming water molecule. In addition, 2–3 water molecules from the broader 124A^•+^ vicinity moved slightly toward 122A. Electronic coupling increased, making the system even more sensitive to solvent fluctuations. A rarely occurring “right” coincidence of structure and solvent fluctuations was required, diminishing the ET probability. Thus, only 3 out of 33 simulations exhibited 124^•+^ → 122 HT.

The **ReA**–124A–122A hopping chain is water-exposed at the monomer protein surface, although 122A is less solvated than 124A, owing to partial shielding by SAL­(A). The solvent access changes profoundly on going to the dimer where the four indoles at the protein–protein interface are solvated by a chain of several quasi-structural water molecules while this whole system is largely shielded from bulk water by protein folds (Figure 9).

**Figure 9:**
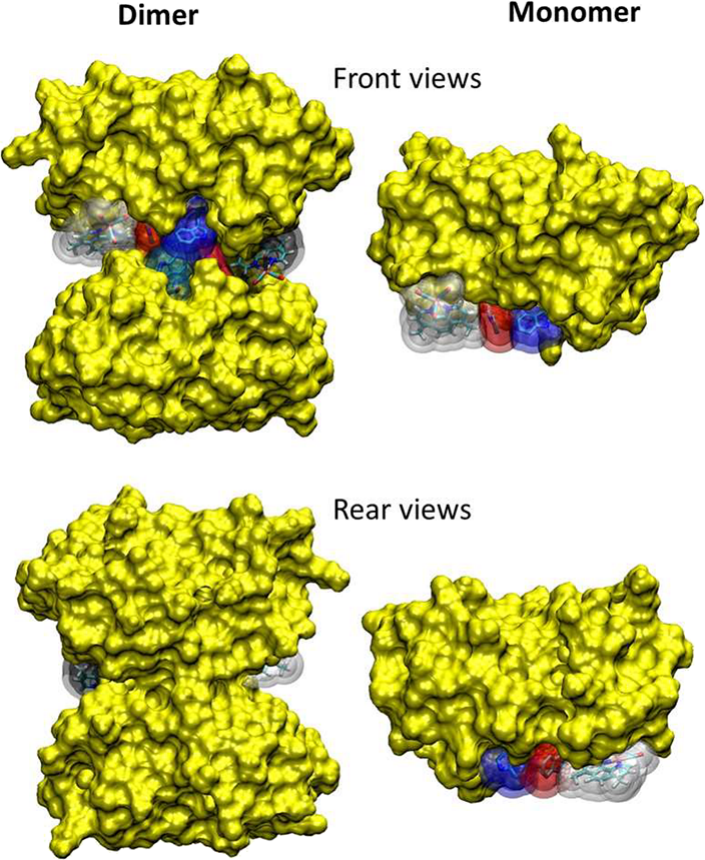
Proximal volumes around ReA^–^, ReD, and indoles in the dimer (left) and monomer (right). The contours are at 2 and 3 Å from heavy atoms.

Although kinetics experiments demonstrated the occurrence of both intramolecular and interfacial hole transport, they could not distinguish individual ET steps.^5^ Here, we examined the propensity of a tryptophan quadruplex to carry electron holes along as well as across a protein–protein interface, especially in view of restricted indole solvation. HT within the quadruplex was assumed to start either at 124A^•+^ or 122D^•+^. QM/MM/MD trajectories did not show HT, which would require unrealistically long simulation times. Nevertheless, the simulations characterized the four states of the oxidized quadruplex and assessed their roles in hole transport.

### Hole Hopping Pathways: Energetics

The relative energetic feasibility of individual HT steps was estimated from differences of electrostatic potentials at oxidized and neutral indoles (Figure 8; obtained from QM/MM/MD) and from Δ*G* values calculated from MM/MD trajectories (Table 1), which led to the same qualitative conclusions. The thermodynamically feasible steps (ΔG ≤ +100 meV) are shown in Scheme 2-left.

**Scheme 2:**
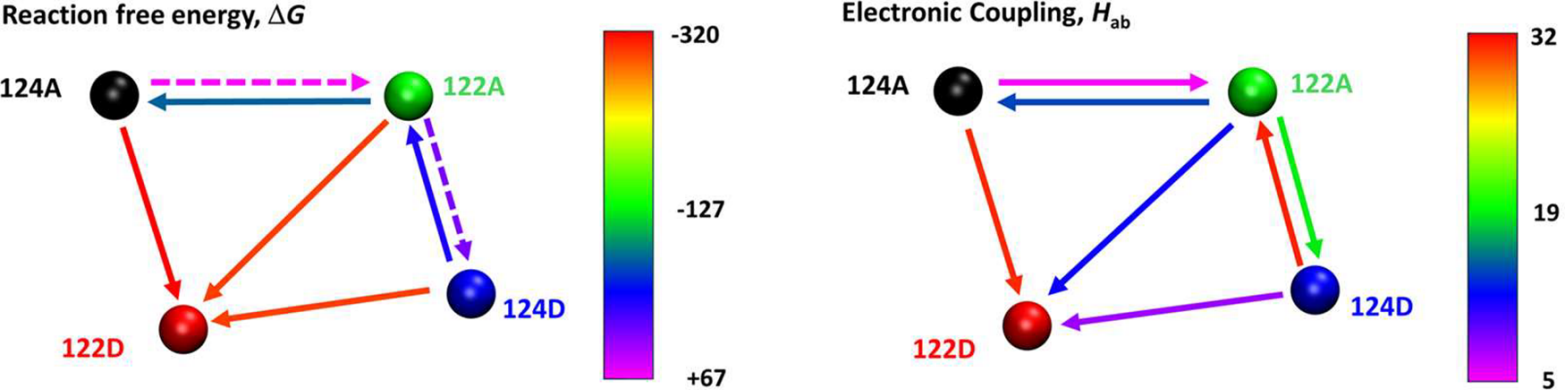
Thermodynamically (Δ*G* < +70 meV) and Kinetically (*H*_ab_ > 0.2 meV) Feasible Hole-Transfer Pathways within the Tryptophan Quadruplex, Color-Graded by the Free Energy Change (Left) and the Median Electronic Coupling (Right)^a^

a All values are in meV. Dashed arrows in the left panel denote uphill steps. Δ*G* and *H*_ab_ for all HT steps are diagrammatically shown in Scheme S1.

Both approaches identified 124A^•+^ → 122D, 122A^•+^ → 122D and 124D^•+^ → 122D HT as the energetically most downhill steps, so that all thermodynamically feasible HT pathways eventually terminated at 122D^•+^ (from which Cu^I^ on chain D is oxidized in a subsequent step). Intramolecular HT 124A^•+^ → 122A was slightly uphill (+67 meV), which would allow establishing a left-shifted equilibrium between 124A^•+^ and 122A^•+^. (This also is the case in the monomer; +11 meV.^4,5^ ) The “dead-end” state 124D^•+^, if populated by uphill (+18 meV) HT from 122A^•+^, would be largely depleted through strongly downhill HT to 122D either directly or through back-HT to 122A. Hence, the 124D^•+^ population would be negligible.

Of interest is that **ReA** ^**–**^ and **ReD** are not just spectators of indole–indole ET, as their charges stabilize 122D^•+^ and destabilize 124D^•+^, affecting the HT energetics. We also found that **ReA** ^**–**^ rotation with respect to 124A inhibited HT in the monomer,^12^ and the same is expected for the dimer. Similarly, rates of all Trp–Trp HT steps in photolyases depend on the flavin oxidation state, presumably owing to subtle structural differences.^16,17^

### Hole Hopping Pathways: Electronic Coupling

The pattern of intramolecular distances was generally matched by electronic couplings, *H*_ab_, whose medians were largest along the short diagonal, closely followed by edges. Couplings along the sides were smaller but still substantial (Table 2, Schemes 2, and S1). However, the broadness of *H*_ab_ distributions suggested the presence of well-coupled configurations for each step. (For example, values for relatively weakly coupled 124A^•+^ → 122A HT were mostly in the units-of-meV range but the distribution tailed toward 60–80 meV). In accord with *H*_ab_ distributions and medians, trajectories of charges and spins occasionally showed moments of partial hole delocalization in tryptophan pairs along the edges and short diagonal 122D–122A.

The strongest coupling was found for 124A^•+^ → 122D and 124D^•+^ → 122A along the edges, suggesting fast and adiabatic interfacial HT producing the 122D^•+^ state (that mediates Cu^I^ oxidation) and depopulation of the 124D^•+^ dead-end state. Diagonal 122A^•+^ → 122D and intramolecular 124A^•+^ → 122A and reverse 122A^•+^ → 124A couplings are weaker but still sufficient for fast HT, especially considering broad *H*_ab_ distributions toward higher values. It follows that energetic (Δ*G*) and electronic (*H*_ab_) factors need to be optimized separately when designing protein systems for electron (hole) transport, since they are not related while both affecting the rate constant.

### Adiabaticity and Role of Hydration

The broad distribution of *H*_ab_ values implies that for each step there are structures with tens-of-meV couplings or higher (Table 2 and S2, and Figures S41–S44), suggesting HT is (at least partly) adiabatic^12,18–20^ (assuming reorganization energy λ = 800 meV and effective nuclear frequency (ν_eff_)^−1^ > 100 fs). HT rates are thus expected to be controlled mostly by environmental fluctuations. Monomer simulations^12^ showed that 124A^•+^ → 122A HT required simultaneous structural and solvational fluctuations that brought their energies temporarily to degeneracy. Such fortuitous coincidence appears less probable in the dimer, where indoles are solvated by an organized “chain” of quasi-structural water molecules at nearly fixed positions that are largely shielded from bulk water by protein folds (Figure 9). Also, the number of water molecules available in the dimer was smaller and some of those facilitating 124A^•+^ → 122A ET in the monomer were not available (Figure S28). On the other hand, the immediate indole solvation in the dimer was tighter and 122A-SAL­(A) and 122D-SAL­(D) moieties were looser compared to the monomer. Water molecules in the quadruplex shifted upon HT by 0.1–0.2 Å toward the oxidized indole, suggesting that solvent fluctuations carrying the system toward the transition state are possible.

### Comparing Hole Hopping Rates

Hole transport through the tryptophan quadruplex (8–11 ns, similar or slightly longer than in the monomer) is 2–3 orders of magnitude slower than through tryptophan chains in photolyases (PL) and cryptochromes (CRY) (units-tens of ps),^16,17,21–23^ even if free-energy changes, indole–indole distances and orientations are comparable, as is the electronic coupling. (Depending on a particular tryptophan pair, *H*_ab_ values of 23 ± 14 and 38 ± 26 meV were calculated for animal 6–4 PL^24^ and 2.5–10 meV for *E. coli* PL.^25^) Relatively slow hole hopping steps within the oxidized quadruplex (as well as in the monomer duplex) are attributable to its rugged potential energy surface (with many local minima), as evidenced by large structural/solvational heterogeneity. Different distributions of quadruplex structures in individual states (Figures 3 and S14–S16) lead to high reorganization energies that include quadruplex restructuring in addition to solvent reorganization and indole/indole^•+^ bonding changes. Structure and solvent fluctuations explore the energy landscape until their low-probability coincidence brings the system to the “right” configurational space, from which HT can occur. These fluctuations likely involve small movements of quasi-structural water molecules within the quadruplex and of protein residues (including **ReA** ^**–**^, **ReD**) as well as changes of H-bonding between waters, indoles and SAL.^5,26,27^ Populating rarely occurring reactive configurations likely is the rate-determining step. Although some microscopic details may differ, HT in the dimer and monomer follow the same general mechanism, in accord with similar immediate indole solvation, without regard to bulk water access. This type of nanosecond adiabatic HT is relevant to hole-hopping in tryptophan/tyrosine chains that protect redox enzymes by removing and deactivating oxidizing equivalents in sequential HT steps.^7–11,28^ On the other hand, tryptophan chains in photoenzymes such as PL, CRY, and plant UV-light receptors^29^ are evolution-optimized, whereby the chromophore, tryptophan residues, and corresponding environments are structurally more homogeneous, being organized in more rigid configurations from which ultrafast HT occurs as a nonergodic process with very small effective reorganization energy.^18,30–35^

### Tryptophan Clusters in Naturally Occurring Oxidoreductases

**{Re126W124W122Cu** ^**I**^ **}**_**2**_ is a protein construct originally designed to extend an electron (hole) transport pathway.^4^ Dimerization emerged spontaneously, owing to the propensity of surface tryptophan residues to stabilize protein–protein interfaces by ππ interactions, dispersion, and hydrophobic forces.^5,14^ A tryptophan quadruplex (together with embedded “structural” water molecules) emerged as a stable structural motif where indole–indole interactions couple two protein units electronically and provide ET pathways. An interesting question arises whether Nature uses tryptophan clusters to mediate ET in multiprotein redox- and photoenzymes. A search of 3906 oxidoreductase (EC-1) X-ray structures found 311 four-tryptophan units (Supporting Information 2), as well as smaller numbers of larger clusters (Figure 10). Out of the 311 4-Trp cases, 216 were intramolecular and 95 interfacial, out of which there were 49 dimers linked by Trp quadruplexes. However, none of these structures had all six shortest indole–indole distances below 5 Å as in **{Re126W124W122Cu** ^**I**^ **}**_**2**_. Distances between naturally occurring quadruplex indoles and other redox centers (flavins, porphyrins, nonheme Fe, NAD^+^, NADH) were rather long for fast ET (10−20 Å). It appears that interfacial tryptophan clusters are rather common among naturally occurring oxidoreductases, but, in many cases, play mostly structural roles. The ET activity of **{Re126W124W122Cu** ^**I**^ **}**_**2**_ indicates that tryptophan quadruplexes could be engineered into de novo proteins and artificial (photo)­enzymes to provide electron (hole) hopping pathways between subunits. In that respect, tryptophan quadruplexes could be considered as protein counterparts to guanine tetrads whose stacks form conductive junctions in DNA constructs.^36^

**Figure 10:**
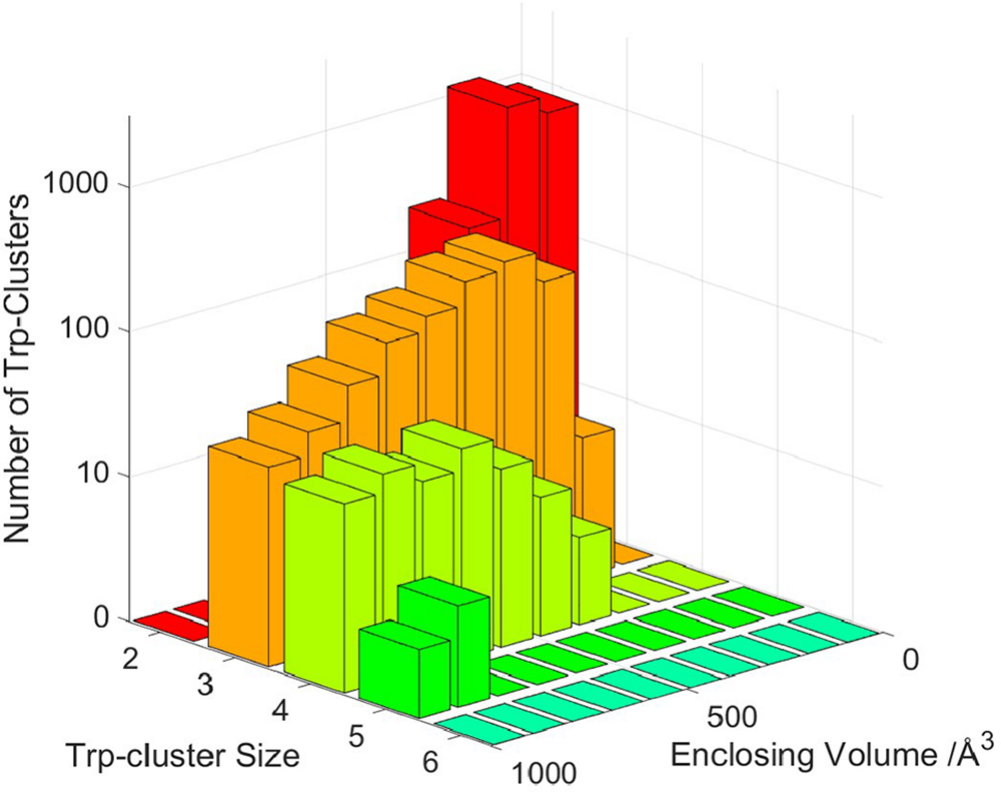
Size-distribution of tryptophan clusters among 3906 pdb-ids for X-ray structures of EC-1 oxidoreductases. The search was restricted to proteins with less than 90% sequence identity. Tryptophans in a cluster were restricted to be within 10 Å of one another (further information on 4-Trp clusters is summarized in Supporting Information 2).

## Conclusions

Neutral as well as one-electron oxidized tryptophan quadruplexes are stable structural motifs of the dimeric azurin construct **{Re126W124W122Cu** ^**I**^ **}**_**2**_ in solution. Besides stabilizing the protein–protein interface, indole–indole interactions couple the two protein units electronically on the order of tens of meV and enable hole transport through the quadruplex. Interfacial HT from the photooxidized 124A^•+^ to 122D and then to Cu^I^ on chain D is preferred thermodynamically as well as kinetically over intramolecular HT to 122A (Scheme 2). 122D^•+^ is the most stable state of the oxidized quadruplex. On the other hand, the dead-end state 124D^•+^ is thermodynamically least stable, essentially eliminated from the hole-transport mechanism. Population of the 122A^•+^ site, from which Cu^I^ on chain A can be oxidized, was found unfavorable and kinetically noncompetitive. It appears that the quadruplex in the dimer transports holes predominantly across the interface whereas parallel intramolecular Cu^I^ oxidation could be due to dissociated monomers present in solution.

The quadruplex is solvated by several quasi-structural water molecules disconnected from bulk water. They keep their positions upon switching between the states, while shifting 0.1–0.2 Å toward the oxidized indole and away from the reduced indole^•+^. They promote HT by carrying the system toward the transition state. Although **ReA** ^**–**^ and **ReD** complexes are not directly involved in HT within the quadruplex, they affect its energetics and solvation through electrostatic potentials (negative on the 124A|122D side, positive on the other). Tryptophan clusters are present in many naturally occurring oxidoreductases but crystal structures indicate that they have mostly structural roles. In this study, a tryptophan quadruplex emerged as a functional unit capable of stabilizing protein–protein interfaces and also mediating interfacial hole transport in multiprotein photosystems on a nanosecond time scale. These reactions are slower than picosecond HT between conserved tryptophan residues in photolyases, presumably owing to a much more rugged potential energy surface being explored by protein/solvent fluctuations while searching for reactive configurations.

## Supplementary Material





## Data Availability

Data are openly available from ZENODO, DOI 10.5281/zenodo.17292746 or upon request.

## Notes

MM/MD trajectories, distance distributions, water distribution functions, coordination numbers, 3D maps, charge and spin QM/MM/MD trajectories, distributions of electrostatic potentials and electronic coupling, and computational details (PDF)
Four-tryptophan clusters in EC-1 oxidoreductase structures (PDF)
