# Supplementary material for: Electron Transport through a Tryptophan Quadruplex in a Dimeric Azurin Construct
Source: J Phys Chem B. 2026 Jan 21;130(5):1503–14. doi: 10.1021/acs.jpcb.5c06932 (PMC12884451; doi:10.1021/acs.jpcb.5c06932)
Supplement: Supplementary file 1 [file jp5c06932_si_001.pdf]

Supporting Information 1

Electron Transport through a Tryptophan Quadruplex in a Dimeric Azurin Construct

Martin Melčák,[a,b] Jan Heyda,[a,b] Filip Šebesta,[c,a] Harry B. Gray,*[,d] Stanislav Záliš,*[,a] Antonín Vlček*[,a,e]

[a] J. Heyrovský Institute of Physical Chemistry, Czech Academy of Sciences, Dolejškova 3, CZ-182 23 Prague, Czech Republic
[b] Department of Physical Chemistry, University of Chemistry and Technology Prague, Technická 5, CZ-166 28 Prague, Czech Republic
[c] Department of Chemical Physics and Optics, Faculty of Mathematics and Physics, Charles University, Ke Karlovu 3, CZ-121 16 Prague, Czech Republic
[d] Beckman Institute, California Institute of Technology, Pasadena, California 91125, United States
[e] Department of Chemistry, Queen Mary University of London, E1 4NS London, U.K.

Table of contents

S1. MM/MD trajectories of GS, ^{3}MLCT, and oxidized states S2 - S13

S2. Distance distributions and 2D maps S14 - S16

S3. Hydration S17 - S26

S4. Charge and spin trajectories S27 - S31

S5. Electrostatic potential distributions S32 - S34

S6. Reaction free energies S35

S7. Electronic coupling distributions S36 - S38

S8. ΔG and H_{ab} summary S38

S9. Computational details S39

S9.1. Classical MM/MD simulations S39
S9.2. QM/MM/MD simulations S41
S9.3. Electronic coupling and reaction energy calculations S43
S9.4. Electrostatic potentials S43
S9.5. Proximal volume $V(r)$, coordination number $N(r)$, and distribution function $g(r)$. Spatial distribution function S44

References S46

S1. MM/MD trajectories of GS, ^{3}MLCT, and oxidized states

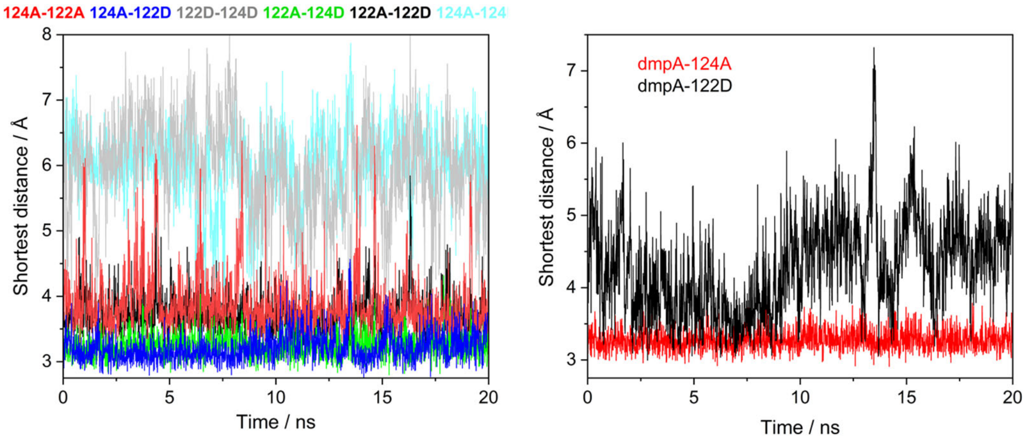

Figure S1. Ground-state MM/MD simulations of indole-indole (left) and dmpA-indole (right) shortest distances (excluding H atoms). Calculated for **{Re126W124W122Cu^{I}}$_2$** composed of equivalent monomeric units, each containing neutral H35. See Figure 1 for labeling of Trp residues.

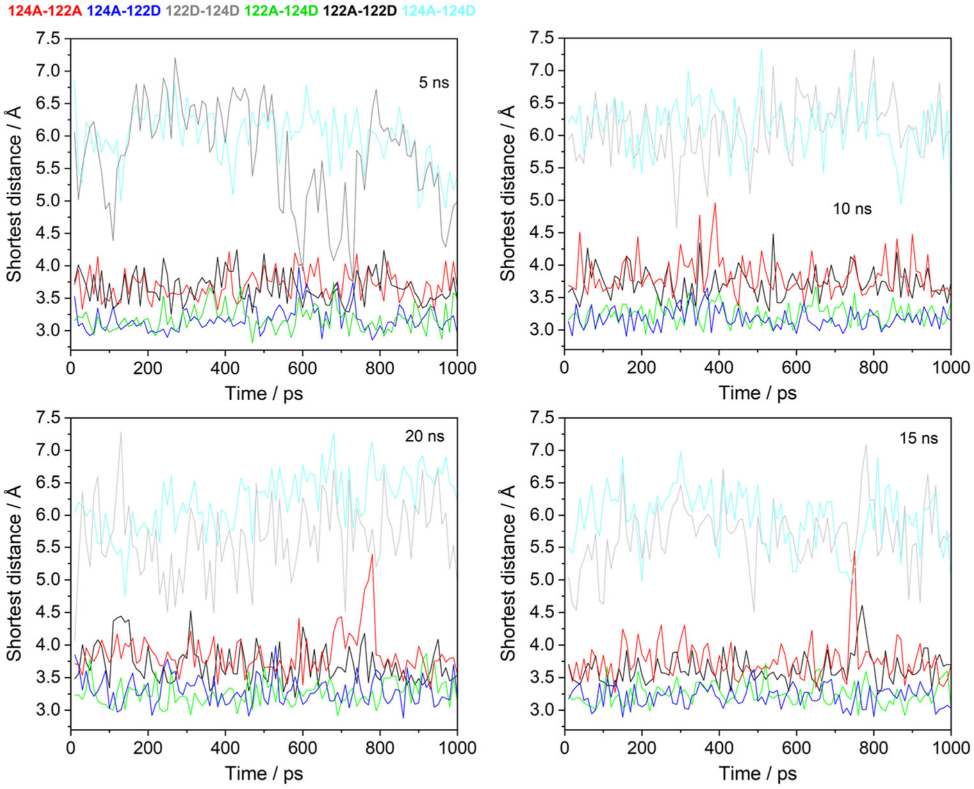

Figure S2. MM/MD trajectories of shortest indole-indole distances in the ^{3}MLCT excited state (H-atoms excluded). Simulations started from GS structures obtained at 5, 10, 15, and 20 ns.)

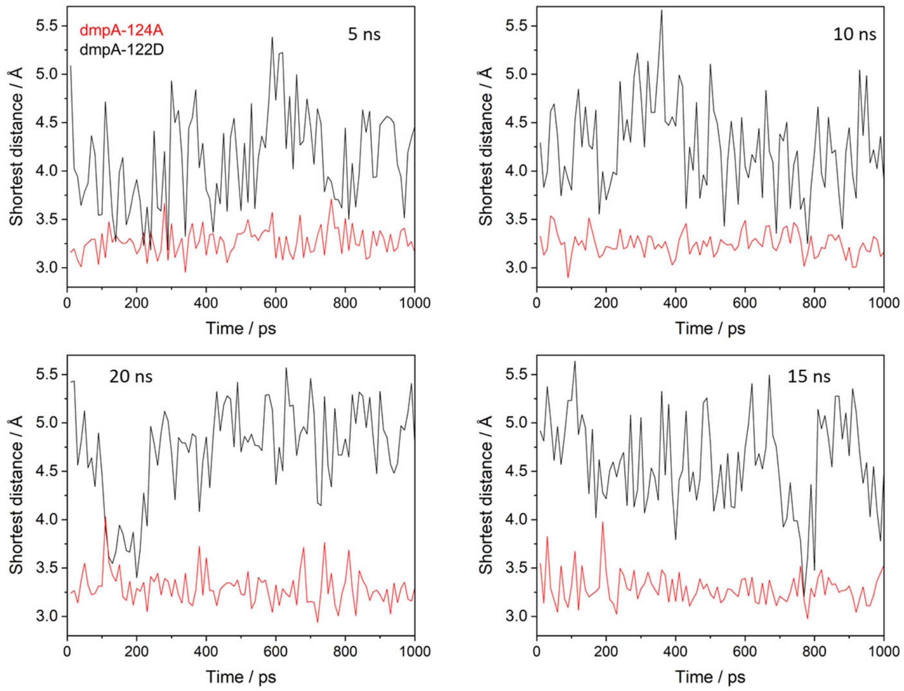

Figure S3. MM/MD trajectories of shortest dmpA-indole distances in the ^{3}MLCT excited state (H-atoms excluded). Simulations started from GS structures obtained at 5, 10, 15, and 20 ns.

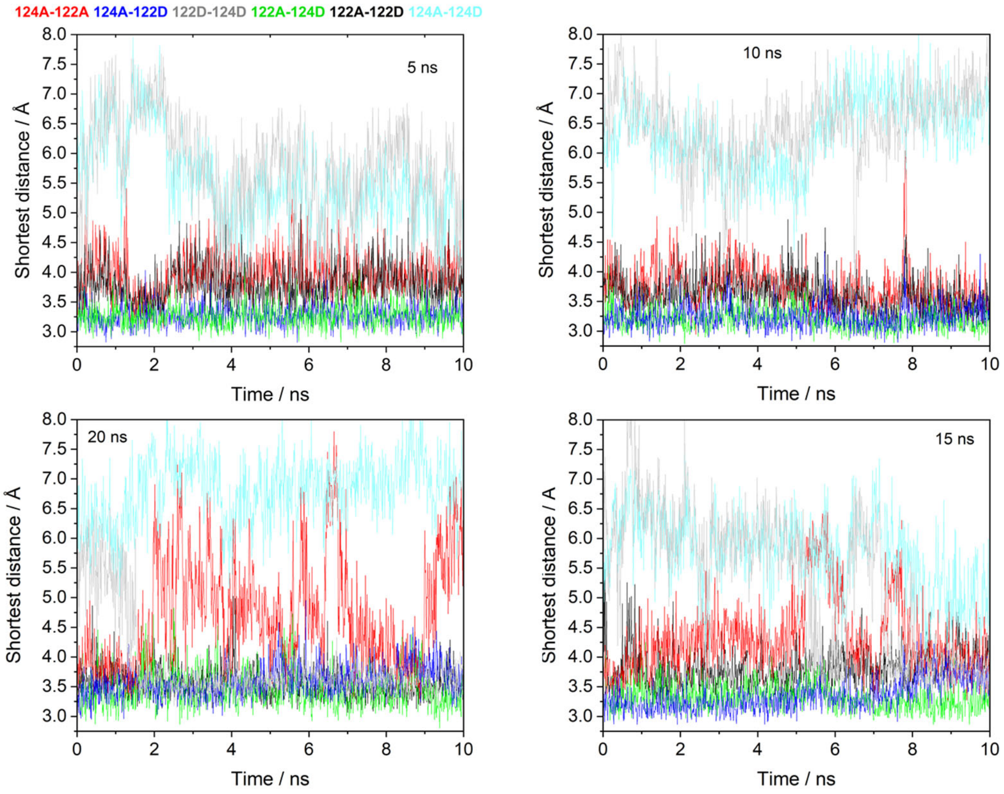

Figure S4. MM/MD trajectories of shortest indole-indole distances in the 124A$^{\bullet+}$ state (H-atoms excluded). Simulations started from end-structures of ^{3}MLCT trajectories specified by their initial GS structures.

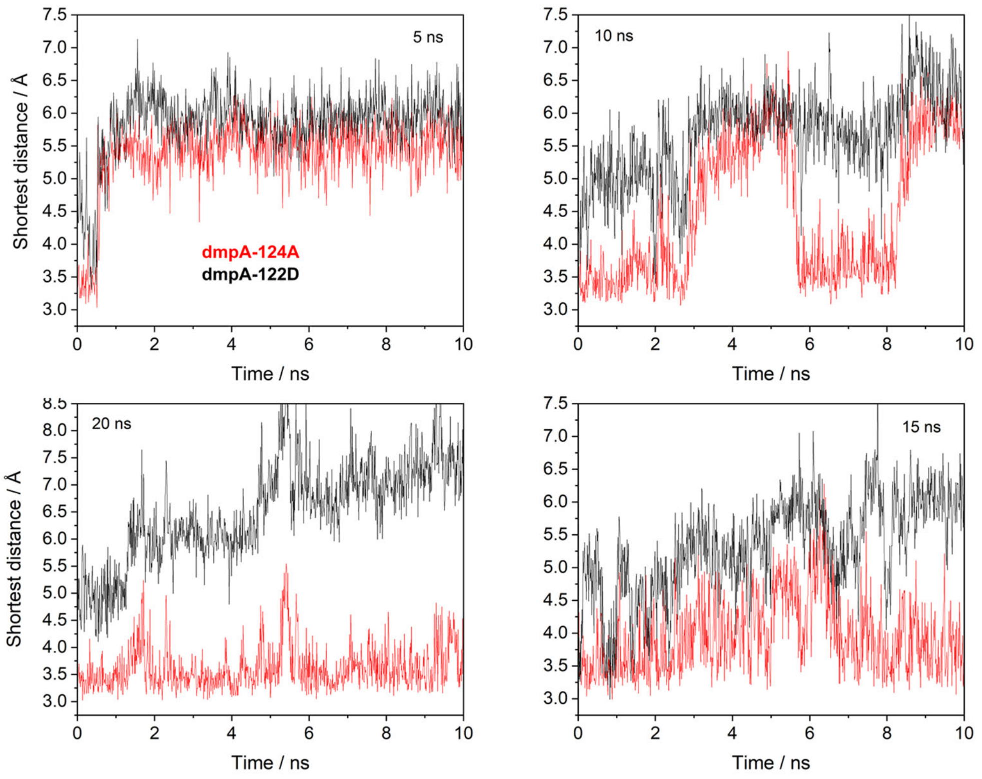

Figure S5. MM/MD trajectories of shortest dmpA-indole distances in the $124A^{\bullet+}$ state (H-atoms excluded). Simulations started from end-structures of MLCT trajectories specified by their initial GS structures.

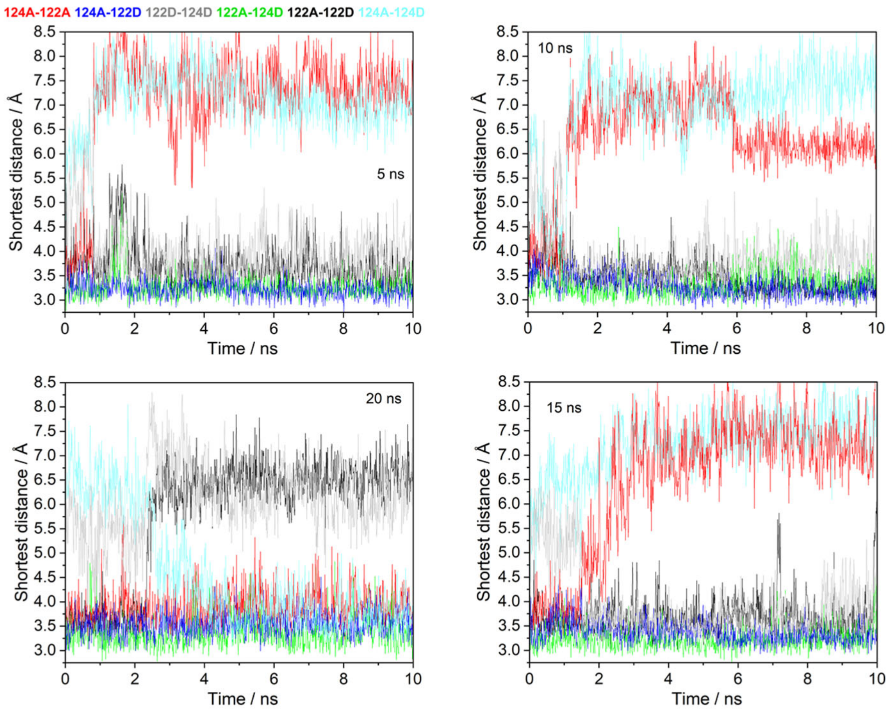

Figure S6. MM/MD trajectories of shortest indole-indole distances in the $122D^{\bullet+}$ state (H-atoms excluded). Simulations started from end-structures of ^{3}MLCT trajectories specified by their initial GS structures.

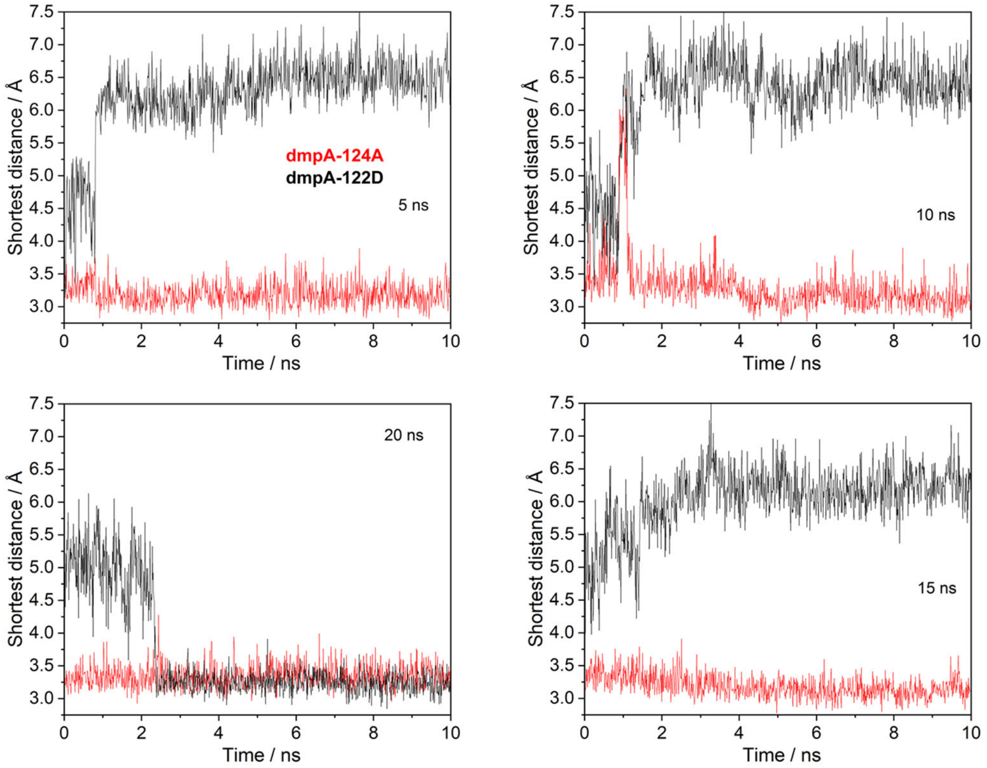

Figure S7. MM/MD trajectories of shortest dmp-indole distances in the 122D$^{\bullet+}$ state (H-atoms excluded). Simulations started from end-structures of MLCT trajectories specified by their initial GS structures.

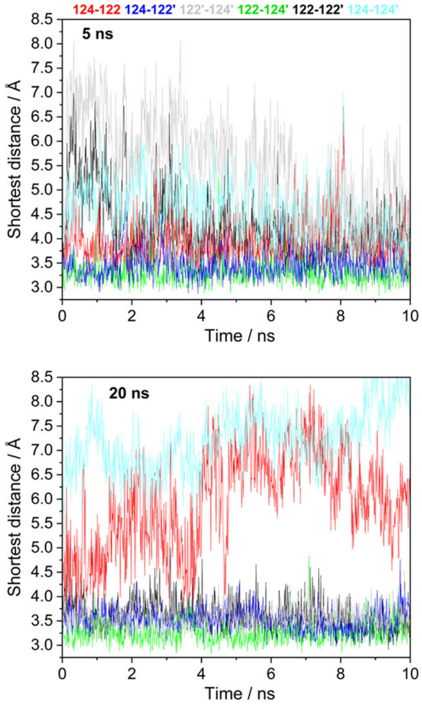

Figure S8. Typical MM/MD trajectories of shortest indole-indole distances in the $122D^{\bullet+}$ state (H-atoms excluded). Simulations started from 4-ns structures of $124A^{\bullet+}$ trajectories specified by their initial GS structures.

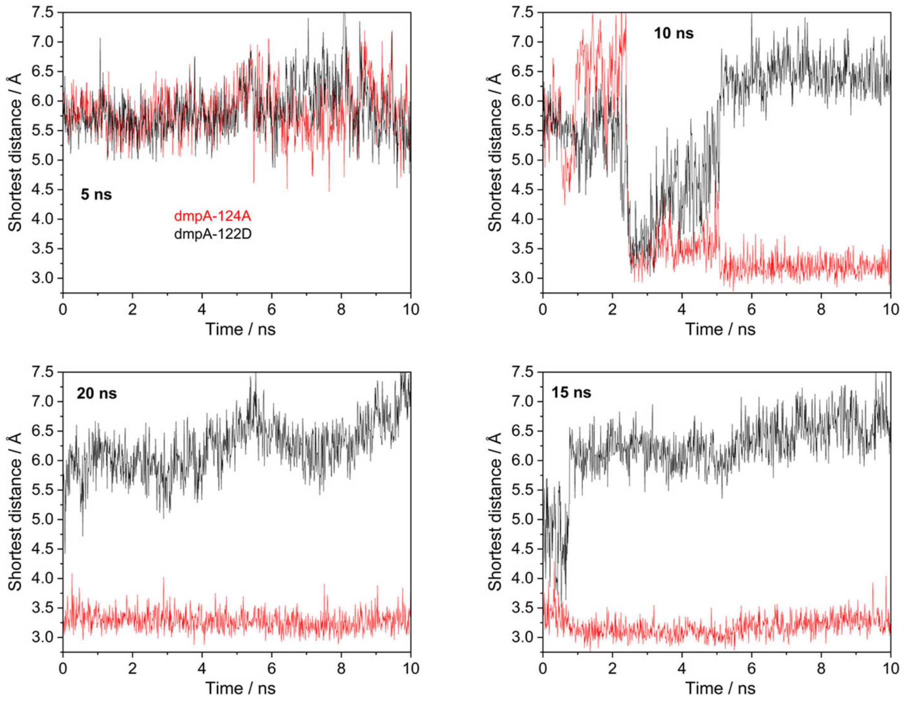

Figure S9. Typical MM/MD trajectories of shortest dmpA-indole distances in the 122D$^{\bullet+}$ state (H-atoms excluded). Simulations started from 4-ns structures of 124A$^{\bullet+}$ trajectories specified by their initial GS structures.

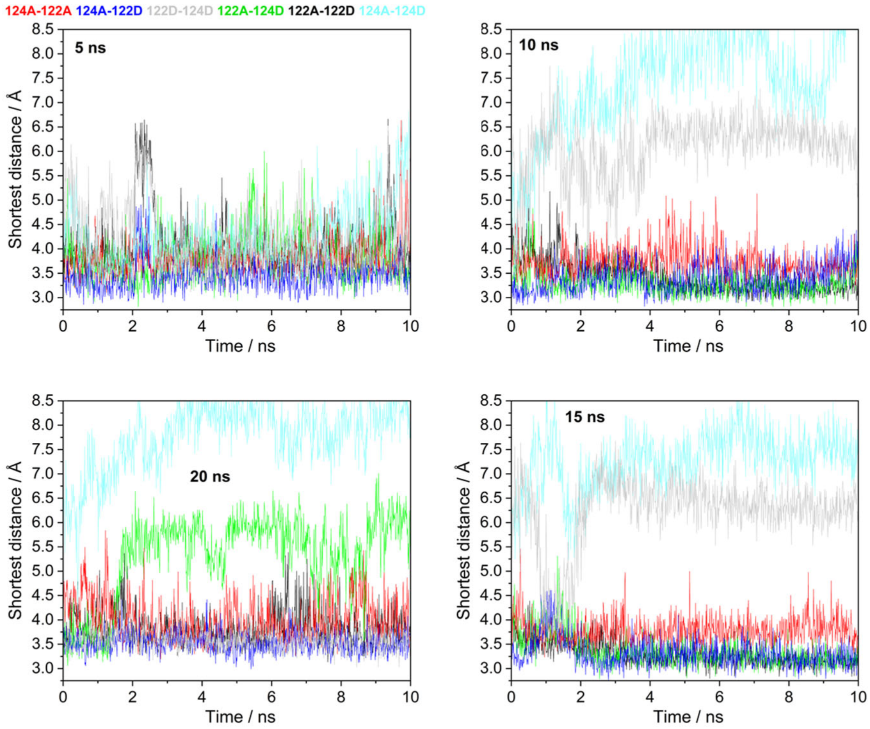

Figure S10. Typical MM/MD trajectories of shortest indole-indole distances in the 122A$^{\bullet+}$ state (H-atoms excluded). Simulations started from 4-ns structures of 124A$^{\bullet+}$ trajectories specified by their initial GS structures.

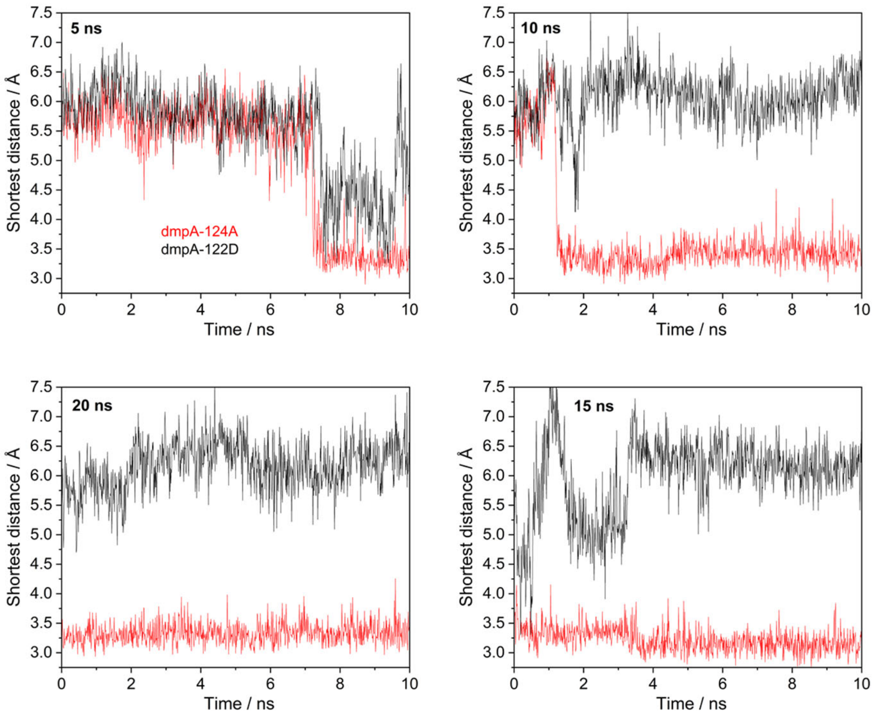

Figure S11. Typical MM/MD trajectories of shortest dmpA-indole distances in the $122A^{\bullet+}$ state (H-atoms excluded). Simulations started from 4-ns structures of $124A^{\bullet+}$ trajectories specified by their initial GS structures.

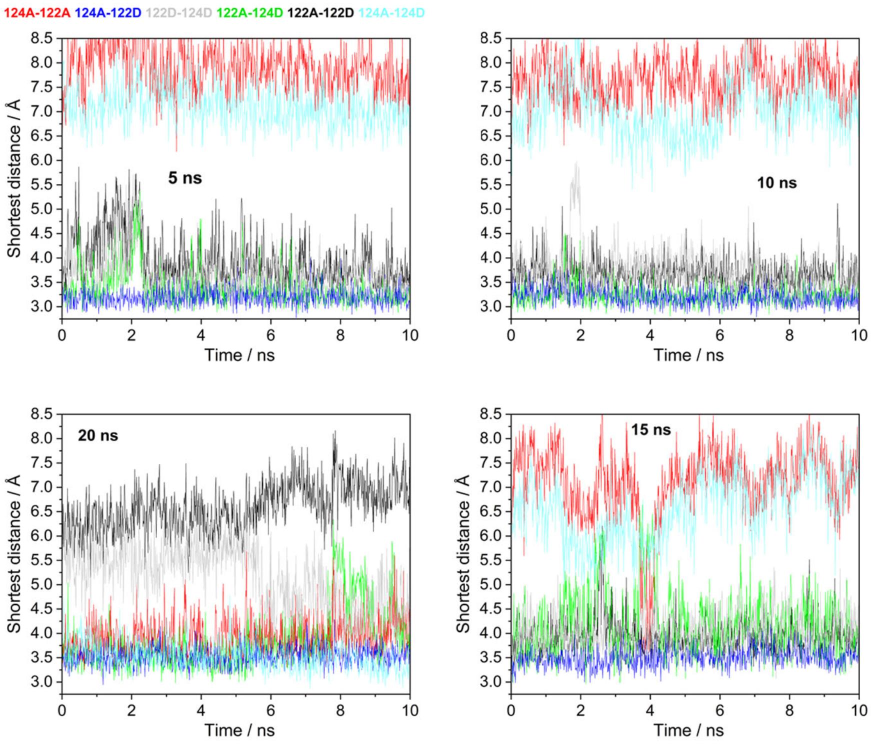

Figure S12. Typical MM/MD trajectories of shortest indole-indole distances in the 124D$^{•+}$ state (H-atoms excluded). Simulations started from 4-ns structures of 122D$^{•+}$ trajectories specified by their initial GS structures.

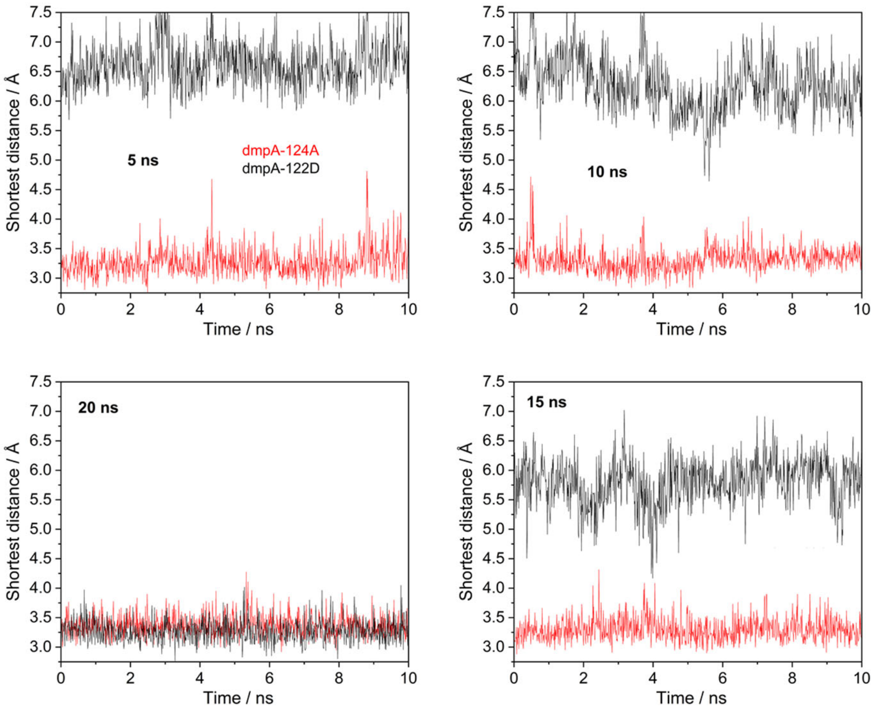

Figure S13. Typical MM/MD trajectories of shortest dmp-indole distances in the $124D^{\bullet+}$ state (H-atoms excluded). Simulations started from 4-ns structures of $124D^{\bullet+}$ trajectories specified by their initial GS structures.

S2. Distance distributions and 2D maps

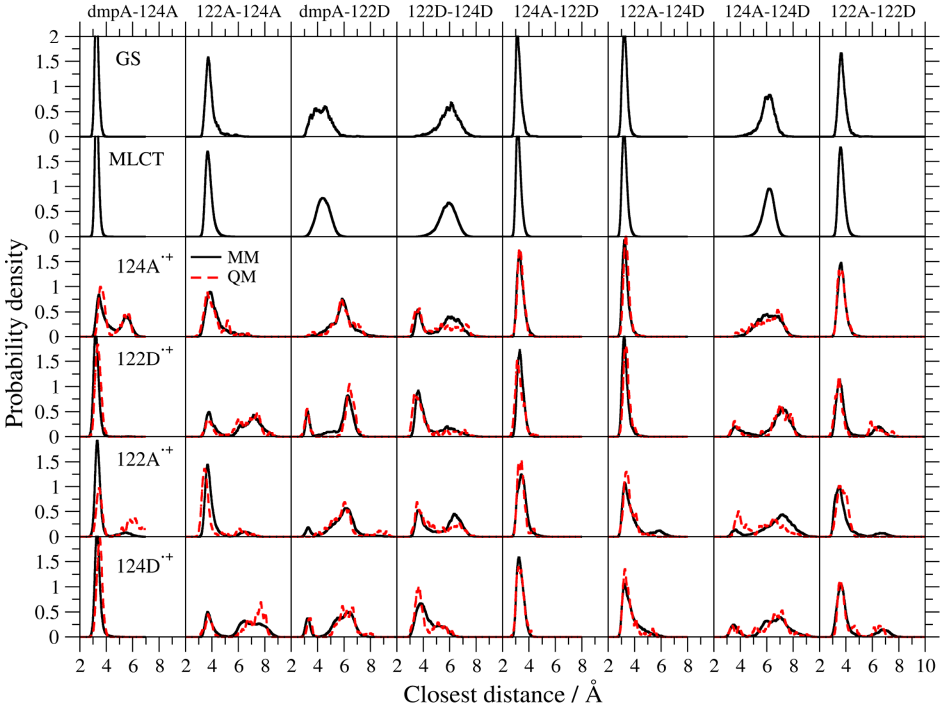

Figure S14. Distributions of closest indole-indole and indole-dmp distances from all MM/MD and QM/MM/MD simulations showing the most ET-relevant distances.

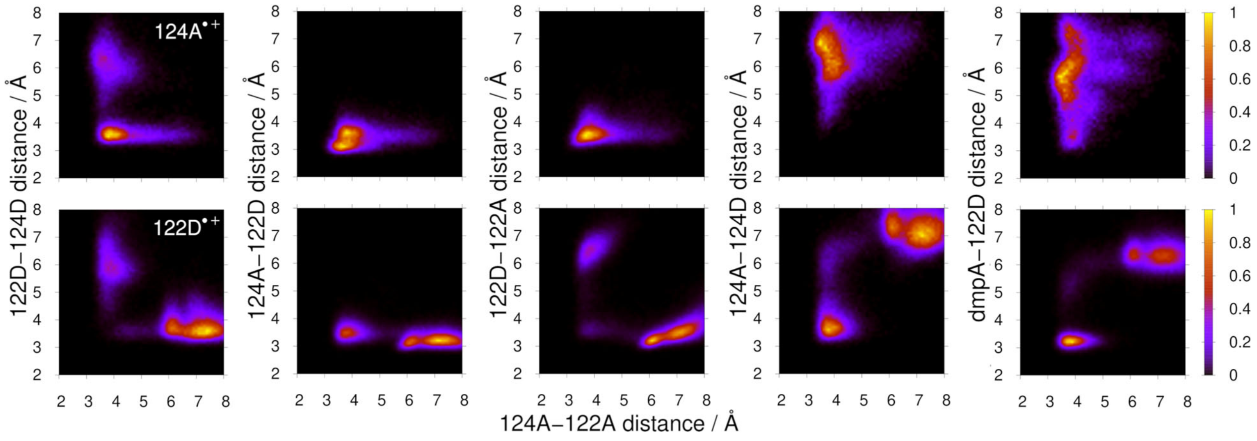

Figure S15. Correlations of selected indole-indole and dmpA-indole shortest distances with the 124A–122A shortest distance. Top row: the $124A^{\bullet+}$ state. Bottom row: the $122D^{\bullet+}$ state. "*in*" **ReA**⁻ orientation selected.

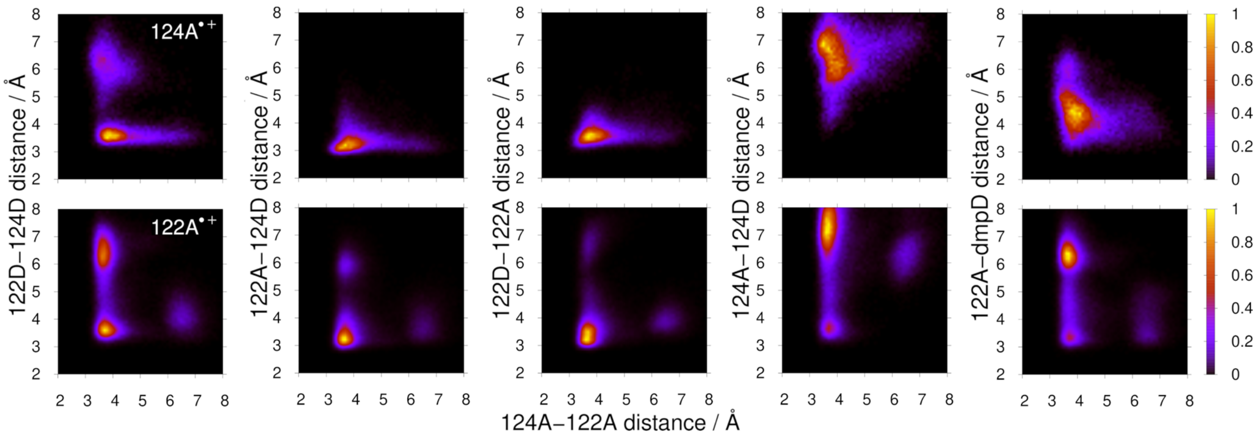

Figure S16. Correlations of selected indole-indole and dmpD-indole shortest distances with the 124A–122A shortest distance. Top row: the $124A^{\bullet+}$ state. Bottom row: the $122A^{\bullet+}$ state. "*in*" **ReA**⁻ orientation selected.

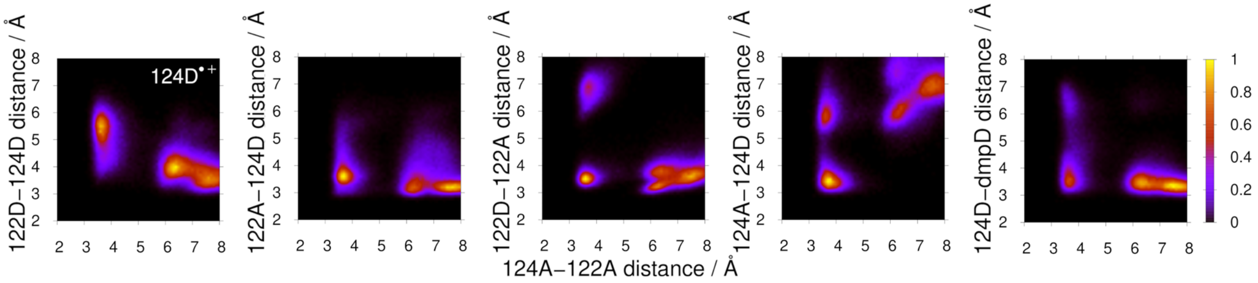

Figure S17. Correlations of selected indole-indole, dmpD-indole shortest distances with the 124A–122A shortest distance in the 124$^{\bullet+}$ state. "*in*" **ReA^{-}** orientation selected.

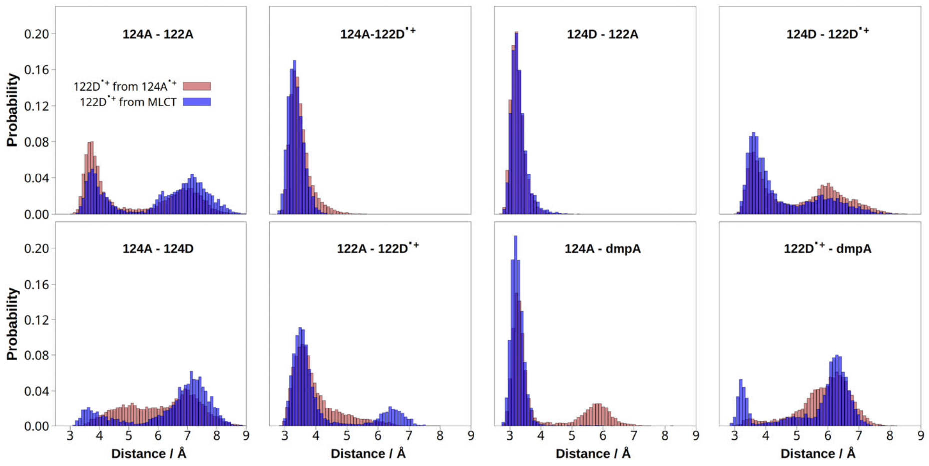

Figure S18. Distributions of selected indole-indole and dmpA-indole shortest distances in the 122D$^{\bullet+}$ state generated from 124$^{\bullet+}$ (red) and MLCT (blue).

S3. Hydration

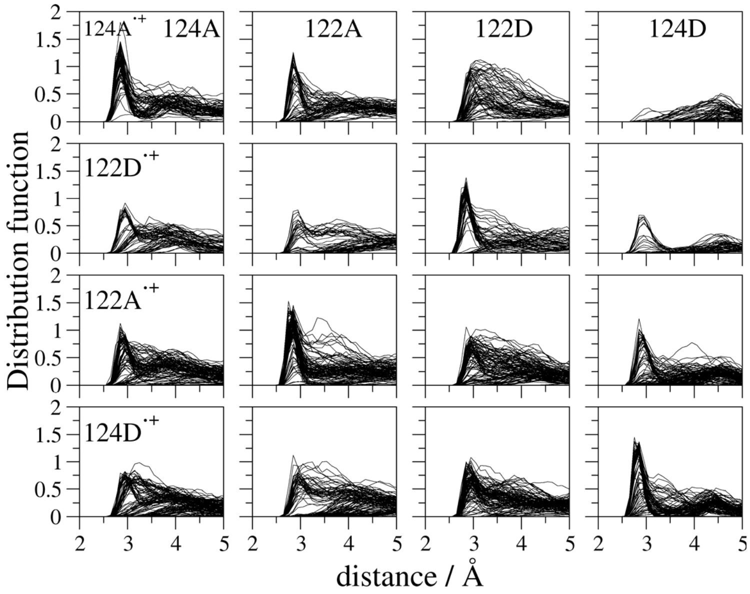

Figure S19. Overlay of water proximal distribution functions at the four indole NH groups in each state calculated from all MM/MD trajectories using the exclusive approach (i.e., each water molecule is accounted to a single closest N atom). The state is specified for each row in the top-left corner of the first panel. The large spread of values and shapes values documents the solvational heterogeneity of the system.

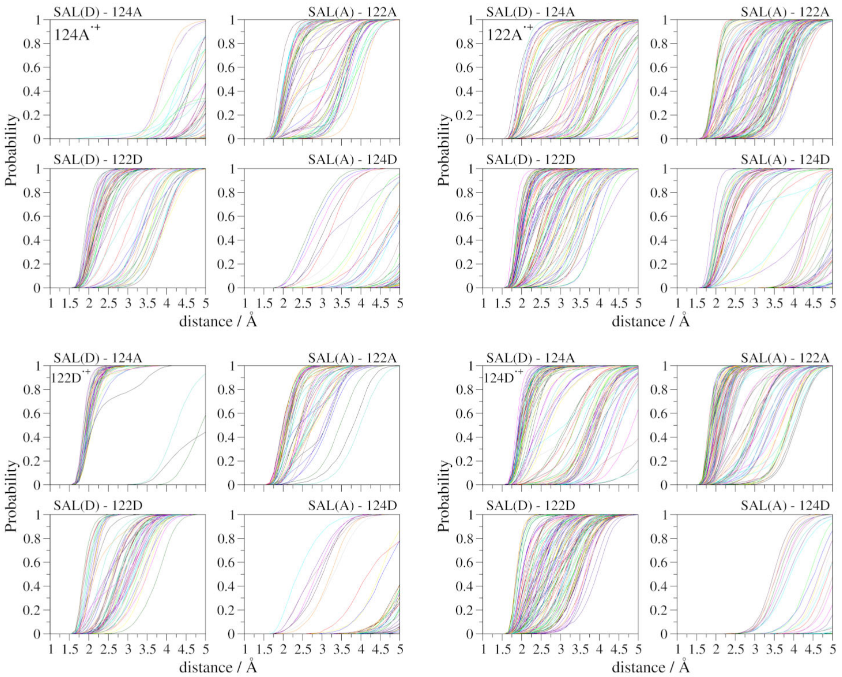

Figure S20. Probability of finding a SAL peptide O atom within a given distance from an indole NH H atom plotted for all MM/MD trajectories. States are specified in top-left corners of each panel. It is assumed that a water molecule can squeeze in and form an indole NH···OH_2 H-bond only for indole NH – O(SAL) distances longer than 2.55 Å.

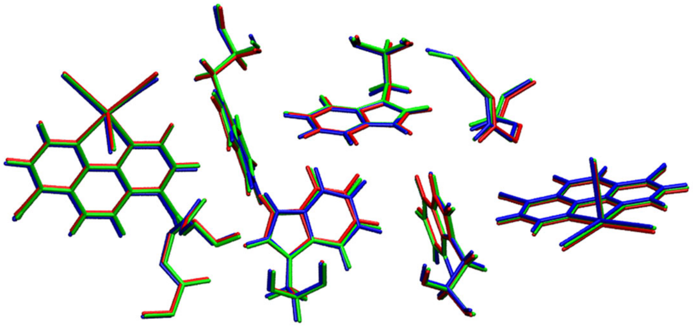

Figure S21. Overlay of average structures of the $124A^{\bullet+}$ (blue), $122D^{\bullet+}$ (red), and $122A^{\bullet+}$ (green) states. (Average structures match each other since geometry ensembles fluctuated comparably in all states.)

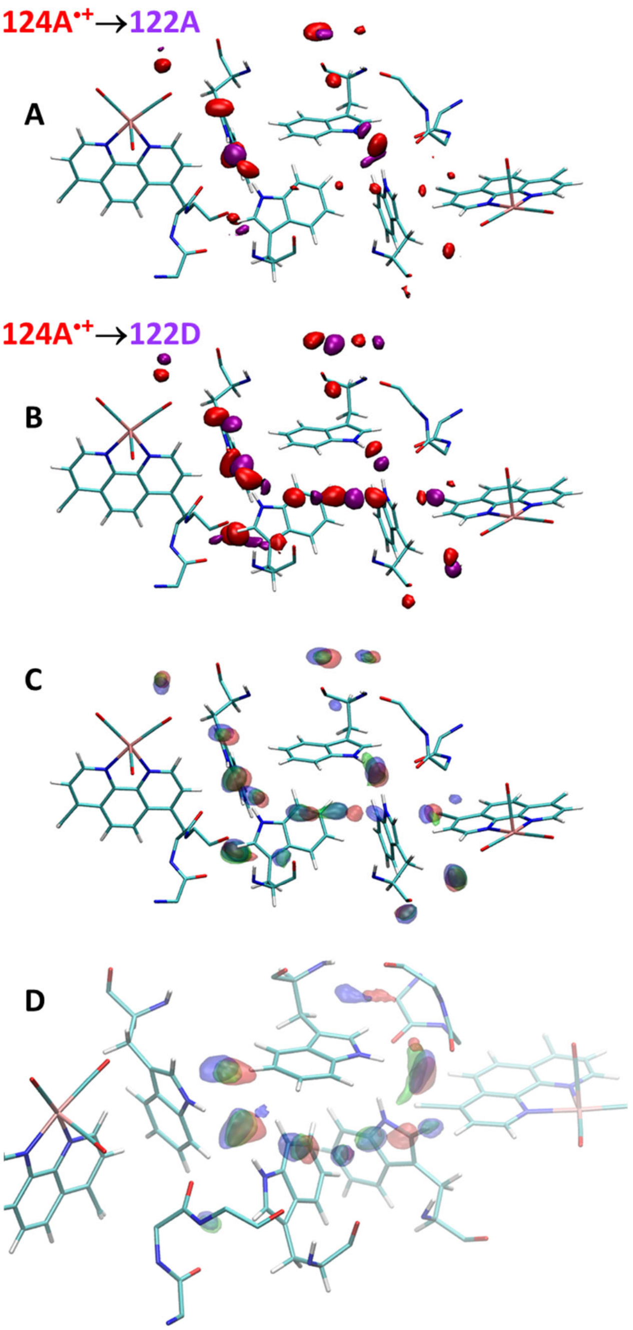

Figure S22. 3D spatial density maps of water oxygen atoms superimposed on the average molecular structure. A, B: Difference 3D spatial density maps showing hydration shifts upon upon $124A^{\bullet+} \rightarrow$ 122D and $124A^{\bullet+} \rightarrow$ $122A^{\bullet+}$ HT. A: regions of excess hydration in $124^{\bullet+}$ (red) and in $122^{\bullet+}$ (violet). B: regions of excess hydration in $124A^{\bullet+}$ (red) and in $122D^{\bullet+}$ (violet). A, B show water oxygen densities within 6 Å from the indoles at a spatial resolution of 0.25 Å. Density differences (ρ_{final} - $\rho_{124A\bullet+}$) were calculated from maps in (C) and are presented at +2.5× (violet) or -2.5× (red) the bulk water density isocontour. C: Overlay of 3D spatial densities of water oxygen atoms at the isocontour of 5× the bulk water density in $124A^{\bullet+}$ (blue), $122D^{\bullet+}$ (red), and $122A^{\bullet+}$ (green) within 6 Å from the indoles with a 0.25 Å resolution. D: Like C, within 4 Å from NH with a 0.1 Å resolution.

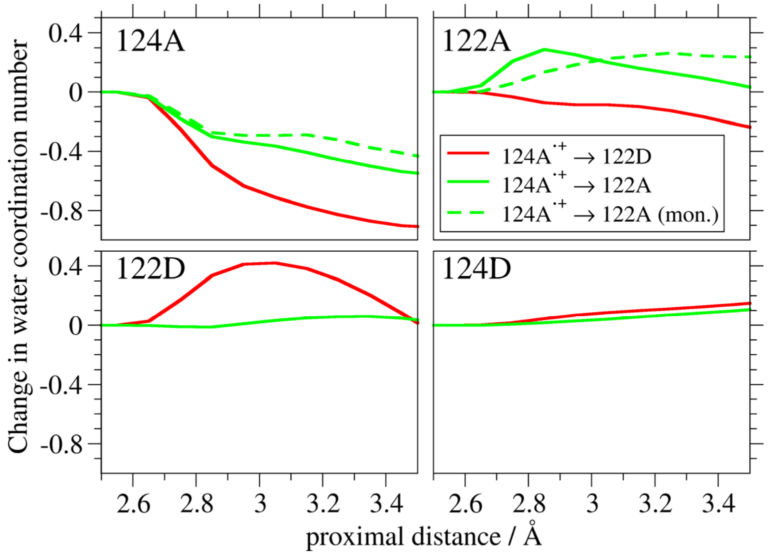

Figure S23. Changes of water coordination numbers of the indole-NH groups upon $124^{\bullet+}$→122D (red) and $124^{\bullet+}$→122A (green) HT. Dashed green: $124^{\bullet+}$→122A in the monomer. (Defined as final state minus $124^{\bullet+}$). Corresponding absolute coordination numbers are shown in Figure S25.

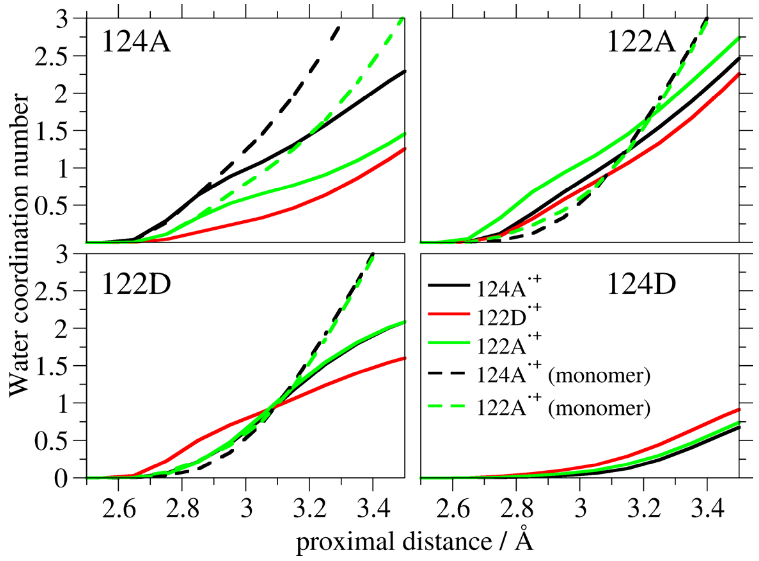

Figure S24. Black: Indole water coordination numbers calculated over the first 100 ps of the $124A^{\bullet+}$ MM/MD simulations starting from nearly rhombic structures with short 124A–122A & 122D–124D distances <4 Å and moderately distant SAL (SAL(A)–122A & SAL(D)–122(D) > 3.5 Å). Green, red: Indole water coordination numbers calculated over the first 100 ps after switching the force-field parametrization to $122A^{\bullet+}$ (green) or $122D^{\bullet+}$ (red). Full lines: dimer; dashed: monomer.

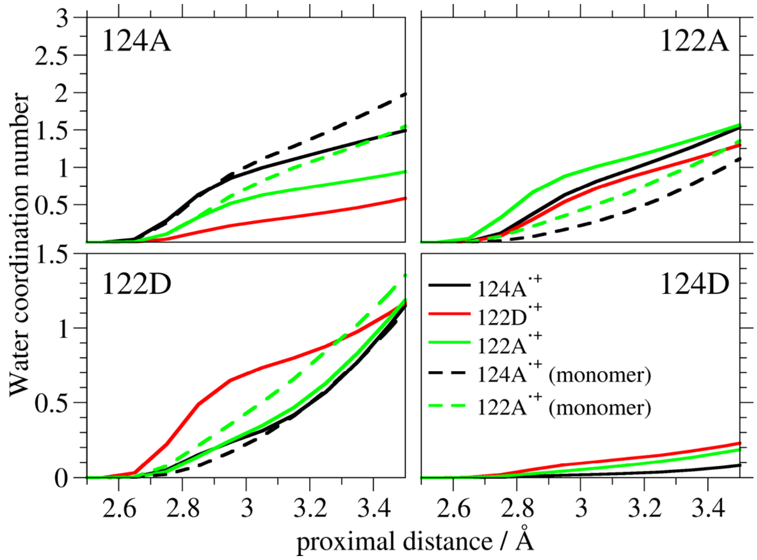

Figure S25. Black: indole-NH water coordination numbers calculated over the first 100 ps of $124^{•+}$ simulations starting from nearly rhombic structures with 124A–122A & 122D–124D distances <4 Å and moderately distant SAL (SAL(A)–122A & SAL(D)–122(D) > 3.5 Å). Green, red: NH water coordination numbers calculated over the first 100 ps after switching the force-field parametrization to $122A^{•+}$ (green) or $122D^{•+}$ (red). Full lines: dimer; dashed: monomer. Note the different scales for the top two and bottom two panels.

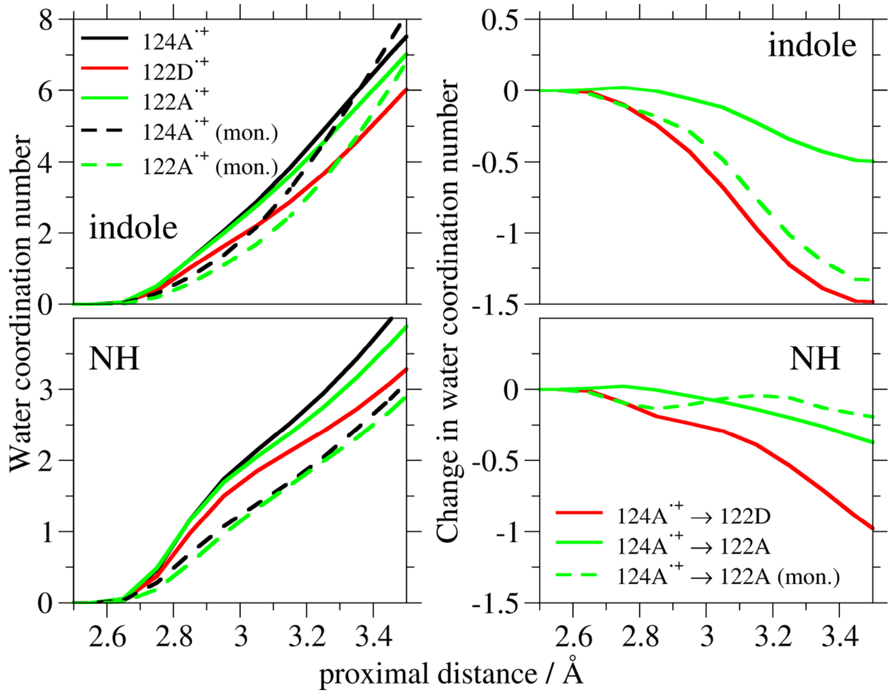

Figure S26. Left column: total solvation of all four indoles (top) and NH groups (bottom) in each state (sums of coordination numbers of individual indoles (Figure S24) and of NH groups (Figure S25), respectively). Right column: differences in total solvation of the indoles (top) and NH groups (bottom) upon HT from $124^{\bullet+}$ to 122A and 122D. (Defined as final state minus $124^{\bullet+}$).

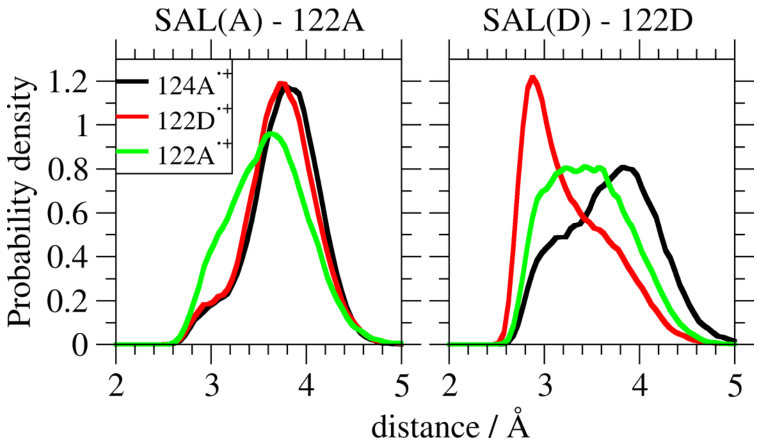

Figure S27. Distributions of SAL(A)-122A (left) and SAL(D)-122D (right) over the 100 ps simulations of the 124A$^{•+}$ state (black) and after switching to 122D$^{•+}$ (red) and 122A$^{•+}$ (green). All starting 124A$^{•+}$ trajectories had SAL(A)-122A and SAL(D)-122D longer than 3.15 Å.

The emergence of a 122D$^{•+}$ subpopulation with short 122D-SAL(D) distances suggested that the 122D$^{•+}$ indole was stabilized by H-bonding to SAL(D) when solvating water molecules were not available. Similar behavior occurred in 122A$^{•+}$ but the subpopulation of short 122A-SAL(A) distances was smaller.

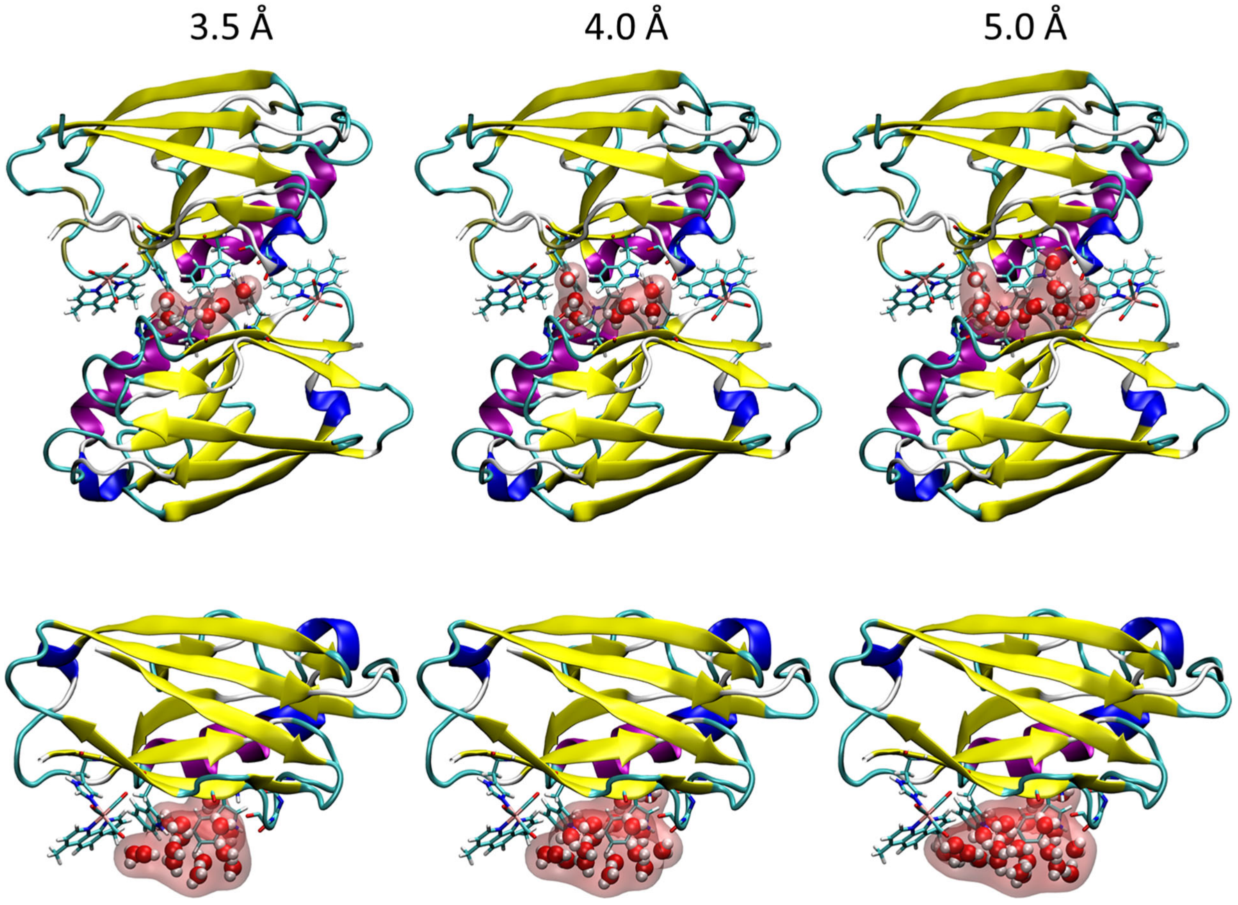

Figure S28a. Structures of the dimer in the $124^{\bullet+}$ state (top row) and monomer in the $122A^{\bullet+}$ state (bottom row) showing water molecules within 3.5, 4.0, and 5.0 Å of indole heavy atoms (measured to water O atoms). Pictured are typical MM/MD snapshots with **ReA**$^{-}$ *in* configurations and short 124-122 distances. (For a detailed view, see Figure S28b below.)
Immediate indole solvation in the dimer and monomer is similar. However, first solvation layer water molecules are connected to bulk water only in the case of the monomer. This is apparent already in the 3.5 Å range that contains 5 water molecules for the dimer and 14 for the monomer.

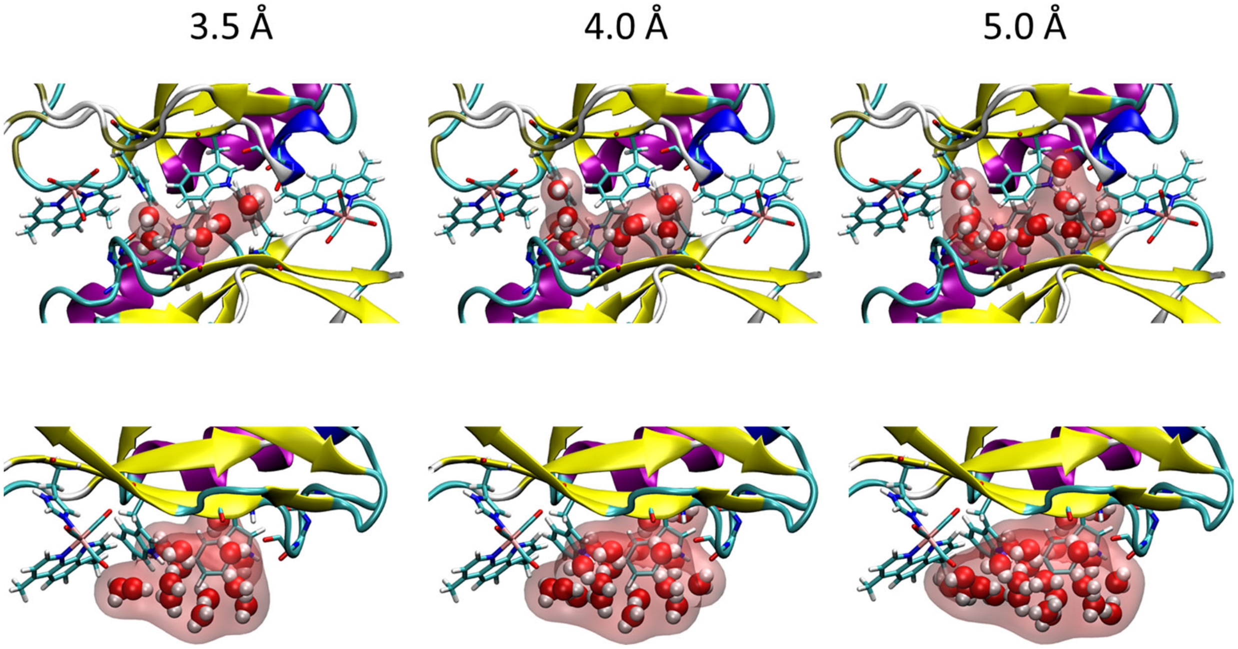

Figure S28b. Detailed view.

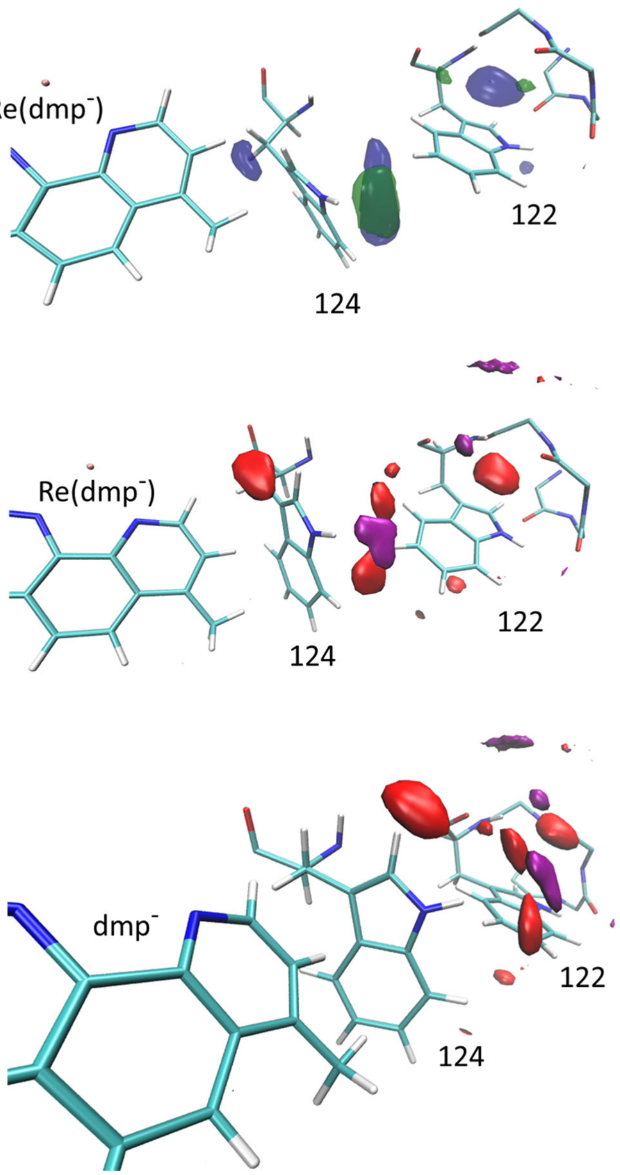

Figure S29. 3D spatial density maps of water oxygen atoms superimposed on the average molecular structure of the monomer. Top: Overlay of 3D spatial densities of water oxygen atoms in the $124A^{\bullet+}$ (blue) and $122A^{\bullet+}$ (green) states, presented at an isocontour of 4× the water bulk density. Middle and bottom: Difference 3D spatial density maps showing regions of excess hydration in the $124^{\bullet+}$ (red) and in $122A^{\bullet+}$ (violet) in two different perspectives. The bottom side view emphasizes the shift of a water molecule away from $124A^{\bullet+}$–NH. (Differential spatial density $\Delta\rho = \rho_{122A\bullet+} - \rho_{124A\bullet+}$ denote the excess water density around $124A^{\bullet+}$ (red) and $122A^{\bullet+}$ (violet). Water oxygen densities are shown within 6 Å from the NH of indoles at a spatial resolution of 0.25 Å. Density differences were calculated from the map at the top of the figure and are presented at +2.5× (violet) or -2.5× (red) the bulk water density isocontour.)

S4. Charge and spin trajectories

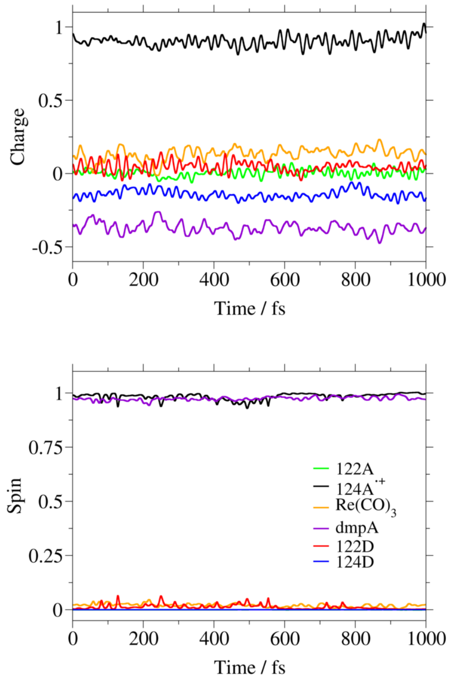

Figure S30. Typical Mulliken charge and spin-density trajectories of the 124A$^{\bullet+}$ state. Spin trajectories indicate occasional small hole delocalization from 124A$^{\bullet+}$ to 122D along the edge. Note the systematic anticorrelation of charge fluctuations at dmpA and Re(CO)$_3$, which was present in the trajectories of all four states. QM/MM/MD simulation employed the CAM-B3LYP functional.

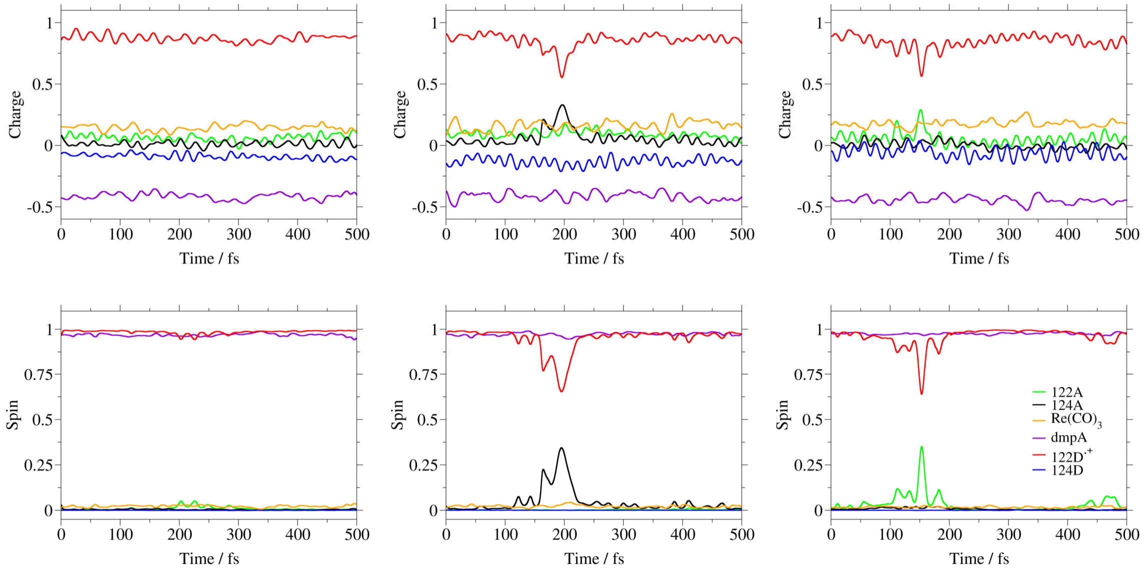

Figure S31. Mulliken charge and spin-density trajectories of the 122D$^{\bullet+}$ state. Left: A typical trajectory of a hole-localized state. Middle: Trajectory showing periods of hole delocalization from 122D$^{\bullet+}$ to 124 along the edge. Right: Simulation showing periods of hole delocalization from 122D$^{\bullet+}$ to 122A along the short diagonal. QM/MM/MD simulation employed the CAM-B3LYP functional.

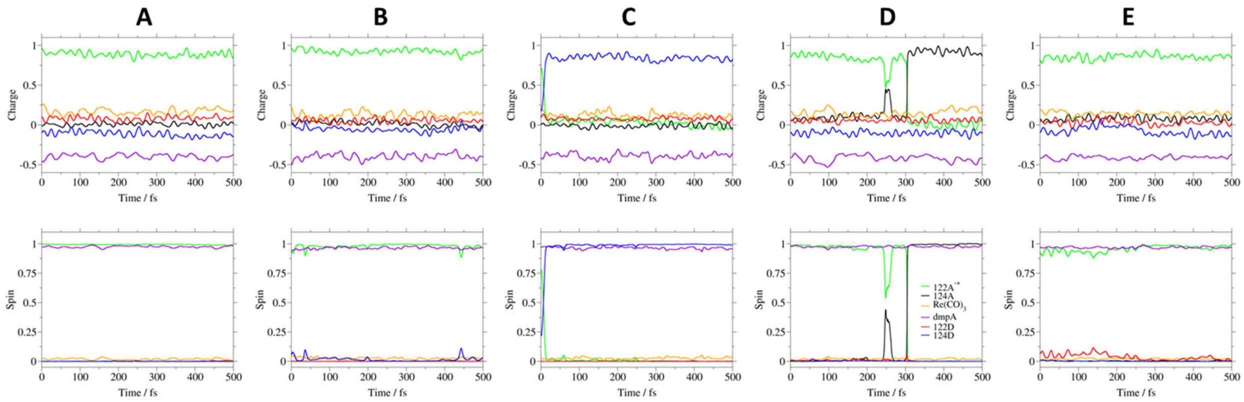

Figure S32. Mulliken charge and spin-density trajectories of the 122A$^{\bullet+}$ states. A, B: Typical trajectories of a hole-localized state. C: The single trajectory showing early conversion to 124D$^{\bullet+}$. D: Trajectory showing a temporary intramolecular hole delocalization from 122A$^{\bullet+}$ to 124A followed by 122A$^{\bullet+}$→124A back HT. E: Trajectory showing minor hole delocalization to 122D along the short diagonal manifested by anticorrelated spin fluctuations. QM/MM/MD simulation employed the CAM-B3LYP functional.

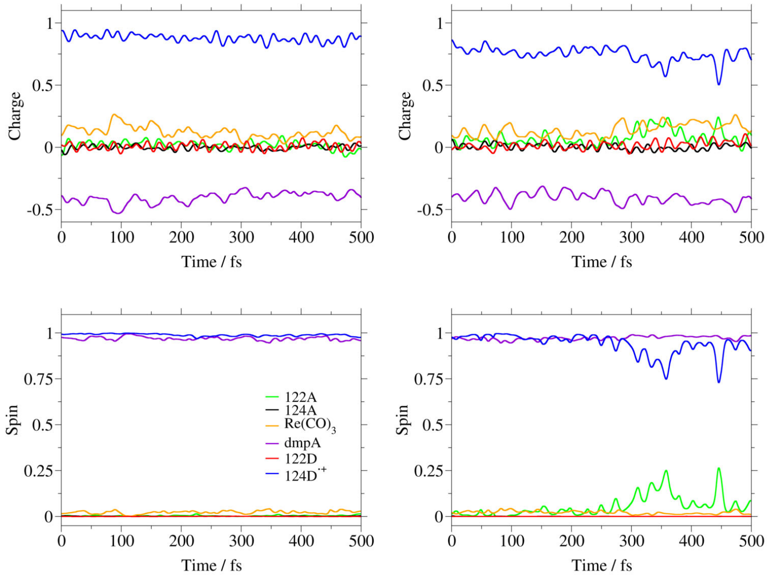

Figure S33. Mulliken charge and spin-density trajectories of the 124D•+ state. Left: A typical trajectory showing hole localized at 124D. Right: Trajectory exhibiting temporary hole delocalization to 122A along the edge. QM/MM/MD simulation employed the CAM-B3LYP functional.

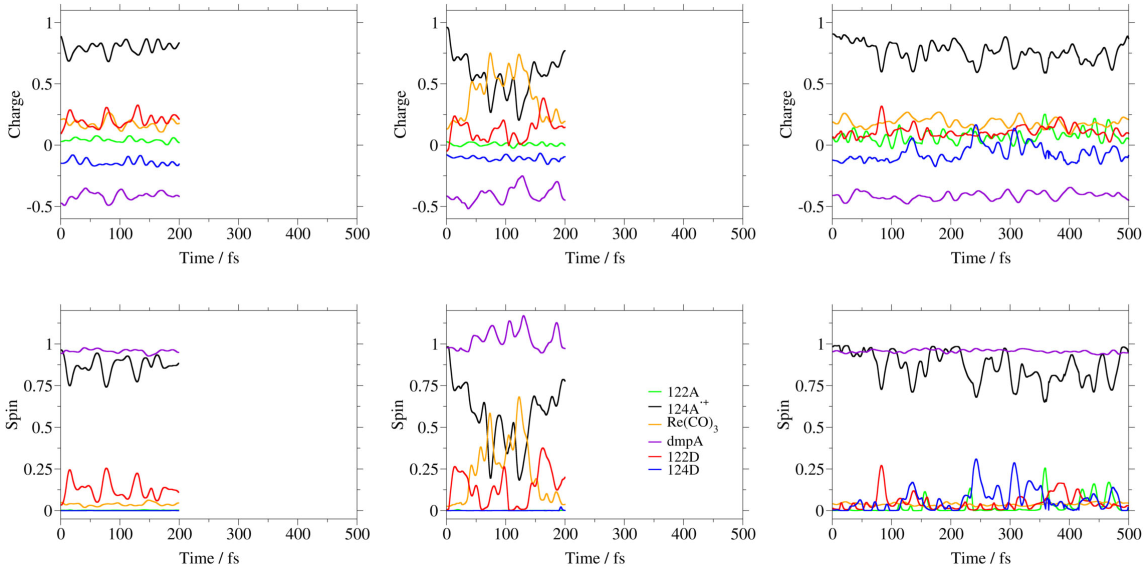

Figure S34. Extensions of 124A•+ QM/MM/MD trajectories using PBE0 functional. Left: A typical trajectory of a mostly hole-localized state showing systematic anticorrelation between Mulliken charge and spin-density fluctuations at 124A•+ and 122D. Middle: Trajectory showing strong 124A•+→Re(CO)$_3$ hole delocalization (yellow). Right: Trajectory showing occasional anticorrelated fluctuations at 124A•+ and 122D (along the edge, red), 122A (along the side, green), and 124D (along the long diagonal, blue; possibly a PBE0 artifact at long distances). Note that delocalization to different indoles occurs in different time periods.

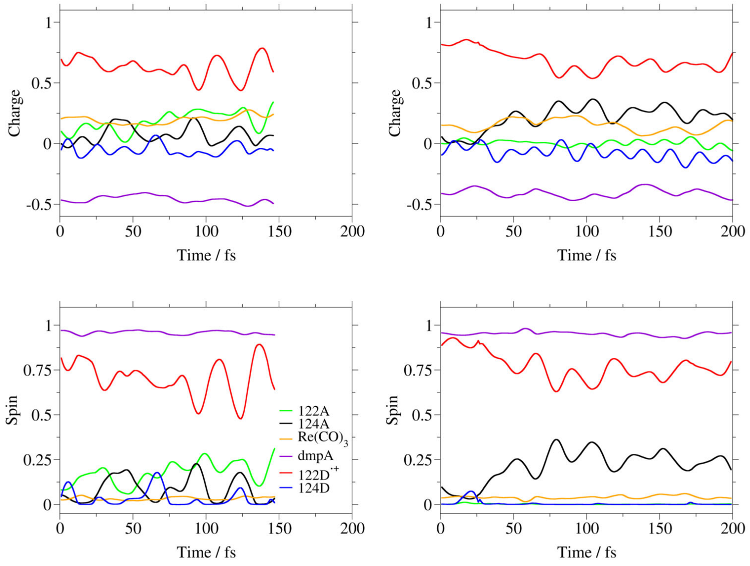

Figure S35. Extensions of 122D$^{\bullet+}$ QM/MM/MD trajectories using the PBE0 functional. Left: Trajectory showing hole delocalization to 124A (along the edge, black), 122A (diagonal, green), and 124D (along the side, blue. It likely occurred in flipped trapezoidal structures with short 122D-124D distances.) Delocalization to different indoles occurred in different time periods. Right: Trajectory showing 122D$^{\bullet+}$→124A hole delocalization (along the edge, black).

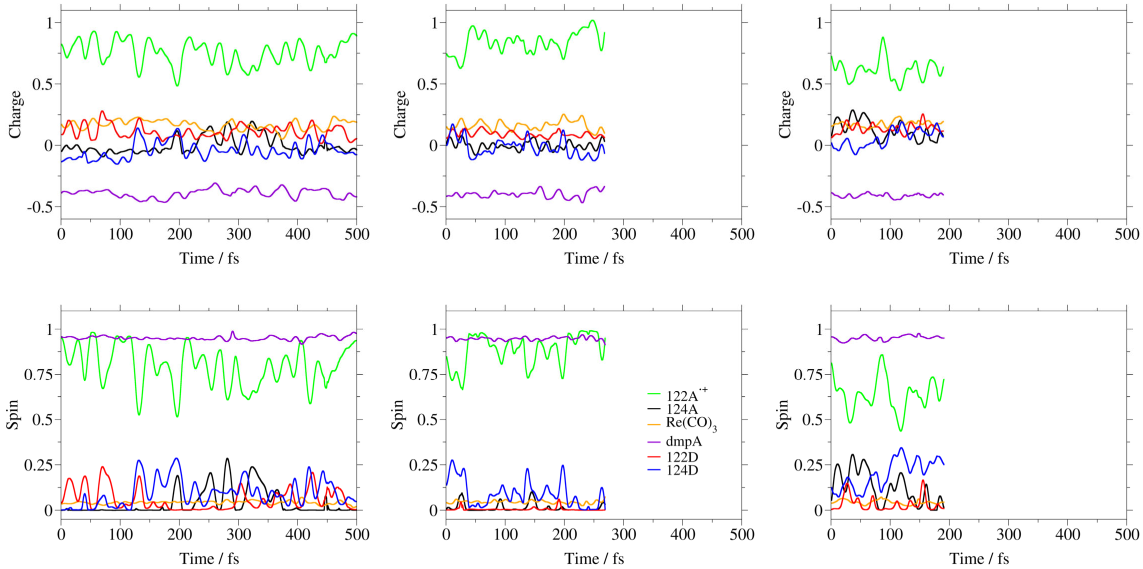

Figure S36. Extensions of 122A$^{\bullet+}$ QM/MM/MD trajectories using the PBE0 functional. All trajectories show hole delocalization from 122A$^{\bullet+}$ to 124D' (along the edge, blue). In addition, there are short periods of delocalization to 122D (diagonal, red) and 124A (side, black). Delocalization to different indoles occurs at different times, probably driven by local structural and solvational fluctuations.

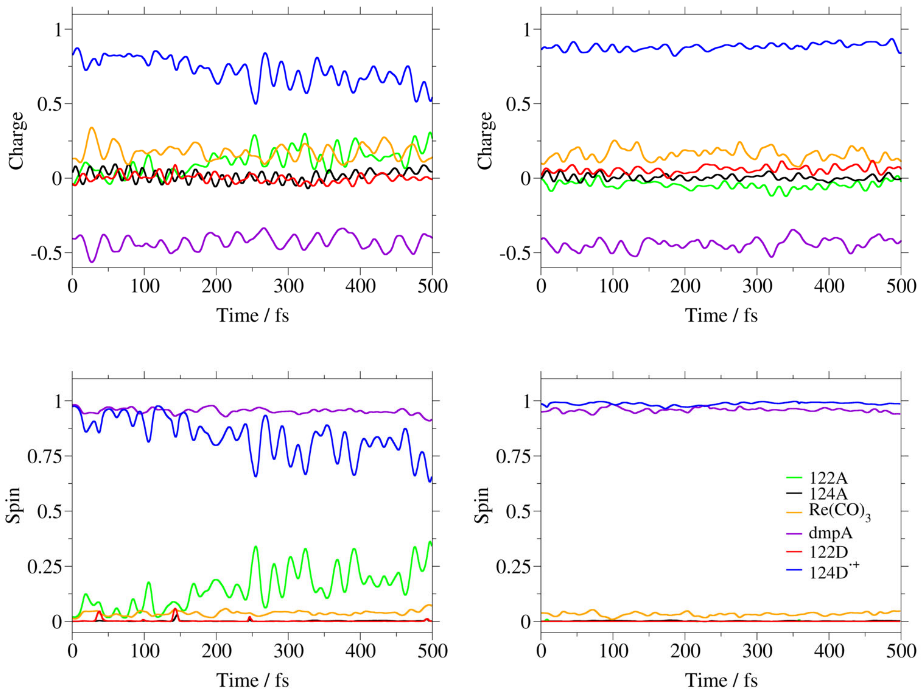

Figure S37. Extensions of 124D•+ QM/MM/MD trajectories using the PBE0 functional. Left: Trajectory showing increasing 124D•+→122A hole delocalization, apparently tending toward conversion to 122A•+. Right: Typical trajectory of a hole-localized state.

S5. Electrostatic potential distributions

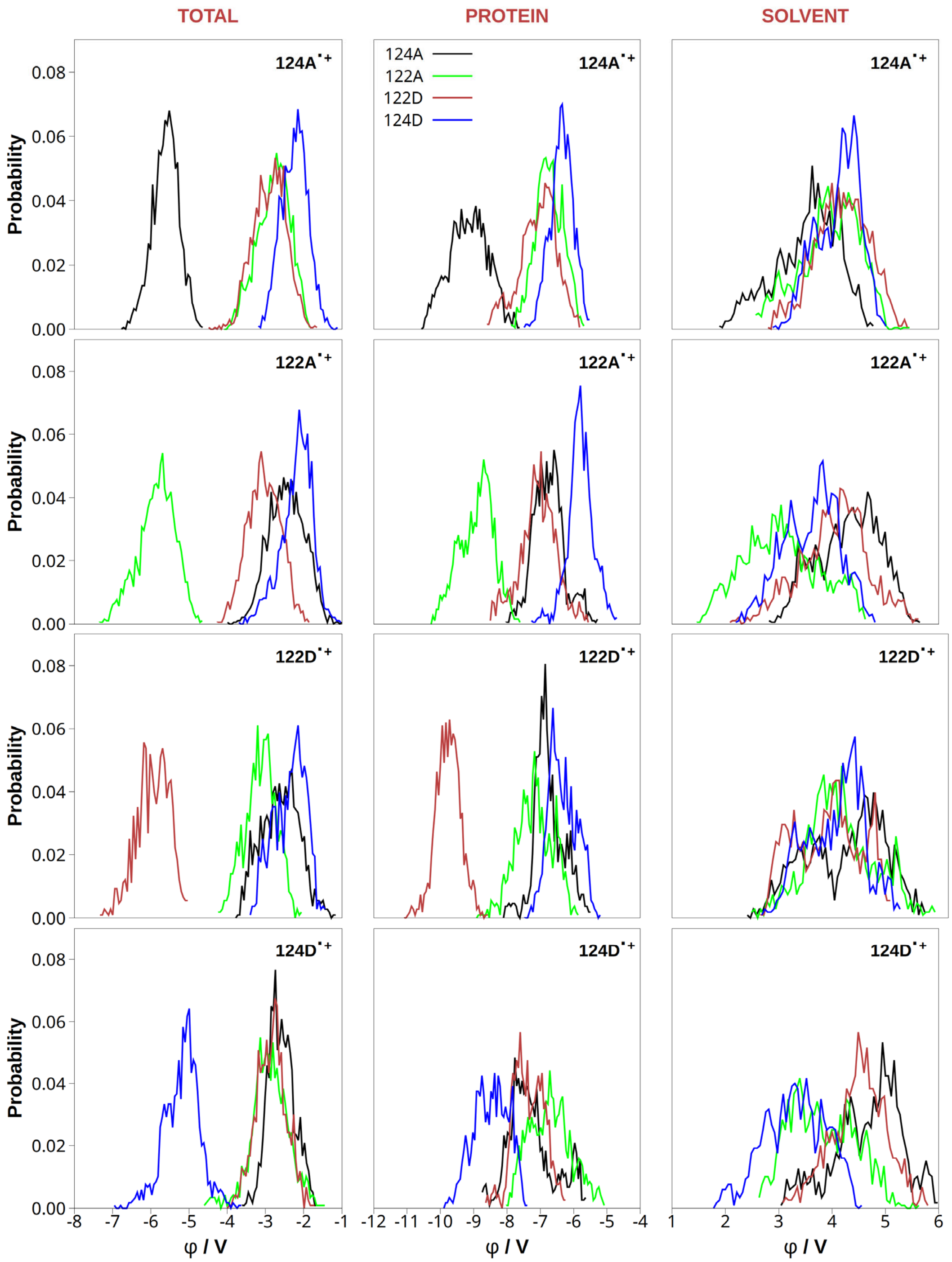

Figure S38. Distributions of electrostatic potentials at the indoles and their protein and solvent contributions. (Oxidized indoles are included in the protein part.)

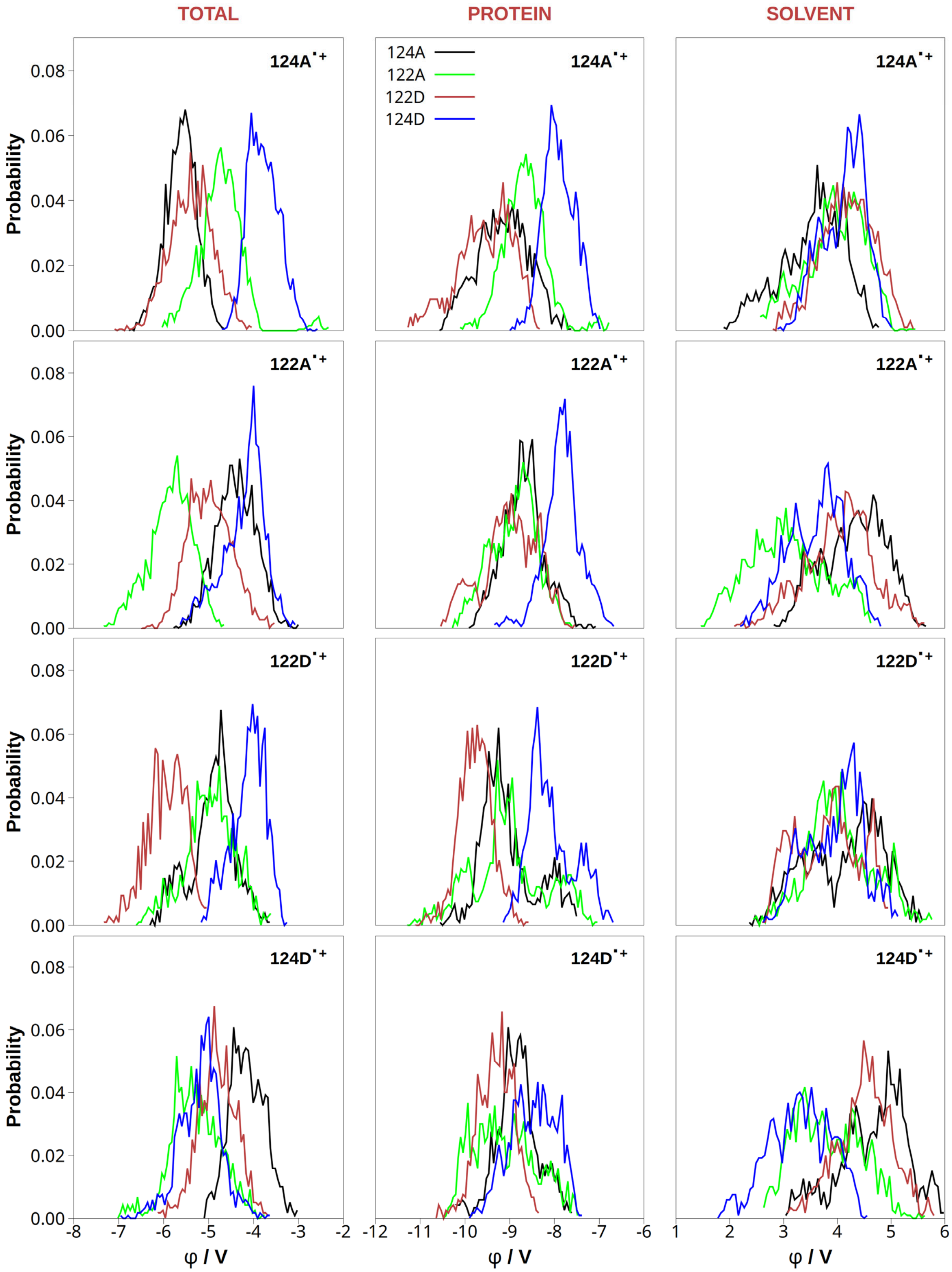

Figure S39. Distributions of electrostatic potentials at the indoles and their protein and solvent contributions. Oxidized indoles were removed from the protein part. Panels in the left column (total potentials) are identical to those in Figure 7 – right.

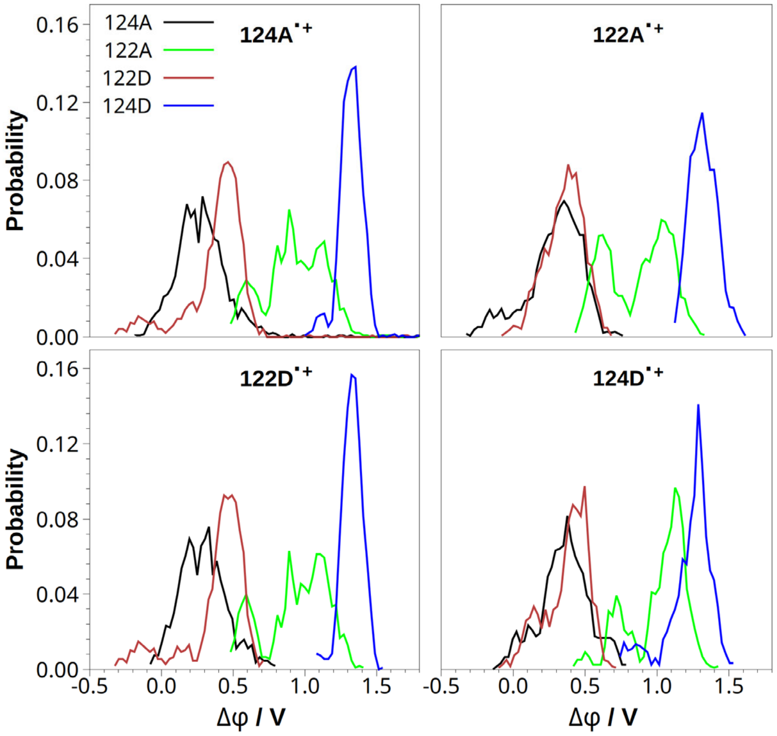

Figure S40. Distributions of electrostatic potentials at the indoles generated by both **ReA**⁻ and **ReD**.

S6. Reaction free energies

Table S1. Reaction free energies (ΔG, meV) of individual HT steps obtained from all MM/MD trajectories.[a] Deviations in parenthesis.[b]

	to 124A	to 122A	to 122D	to 124D
from $124A^{\bullet+}$	-	+67 (80)	-320 (49)	+172 (457)
from $122A^{\bullet+}$	-67 (80)	-	-296 (156)	+18 (81)
from $122D^{\bullet+}$	+320 (49)	+296 (156)	-	+262 (67)
from $124D^{\bullet+}$	-172 (457)	-18 (81)	-262 (67)	-

[a] Calculated according to ref.[1] Reported ΔG values refer to the whole ensemble. b Deviations in parentheses reflect the structural/solvational heterogenity. They were obtained as standard deviations of averages of ΔG values calculated for four subpopulations obtained starting at 5, 10, 15, and 20 ns of the initial GS trajectory.

S7. Electronic coupling distributions

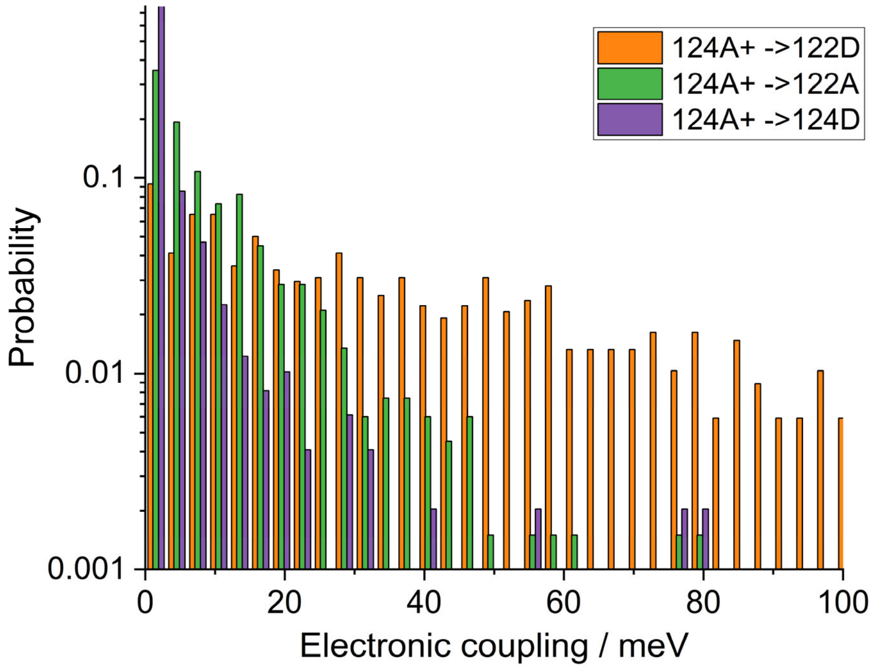

Figure S41. Distribution of H_{ab} values over the 0-100 meV range calculated for ET steps originating from $124A^{•+}$. (Probability is on a log scale.)

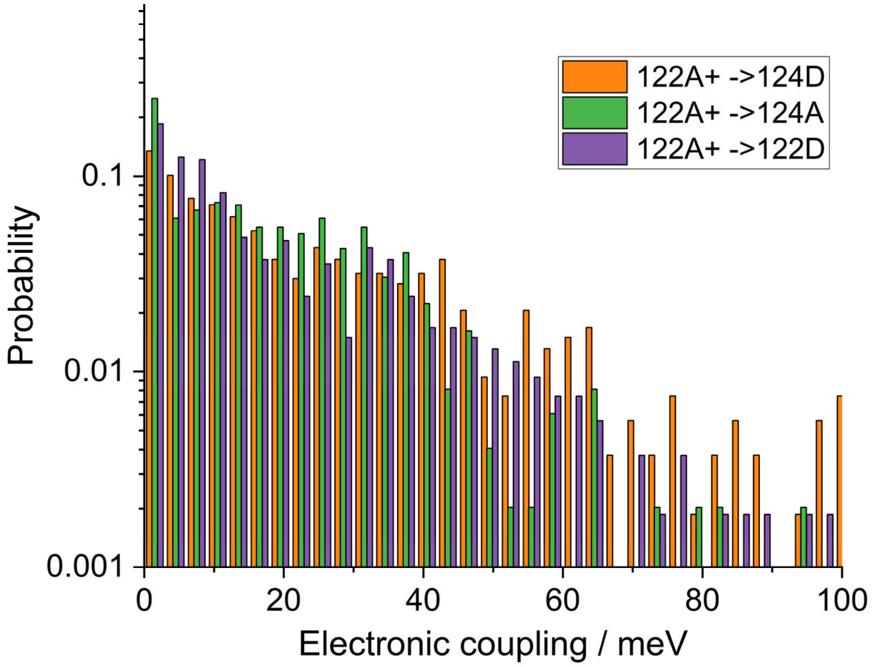

Figure S42. Distribution of H_{ab} values over the 0-100 meV range calculated for ET steps originating from $122A^{•+}$. (Probability is on a log scale.)

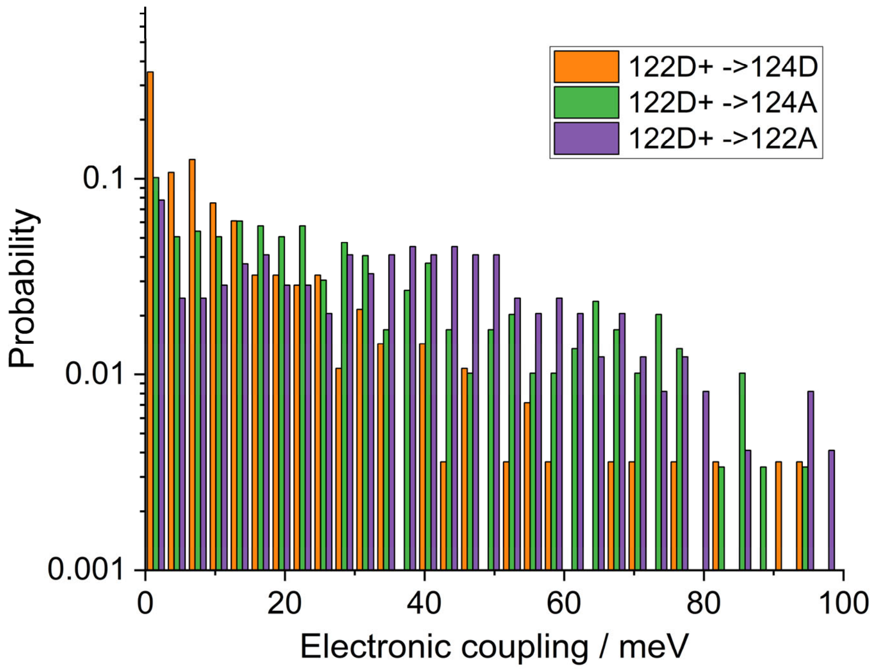

Figure S43. Distribution of H_{ab} values over the 0-100 meV range calculated for ET steps originating from 122D$^{\bullet+}$. (Probability is on a log scale.)

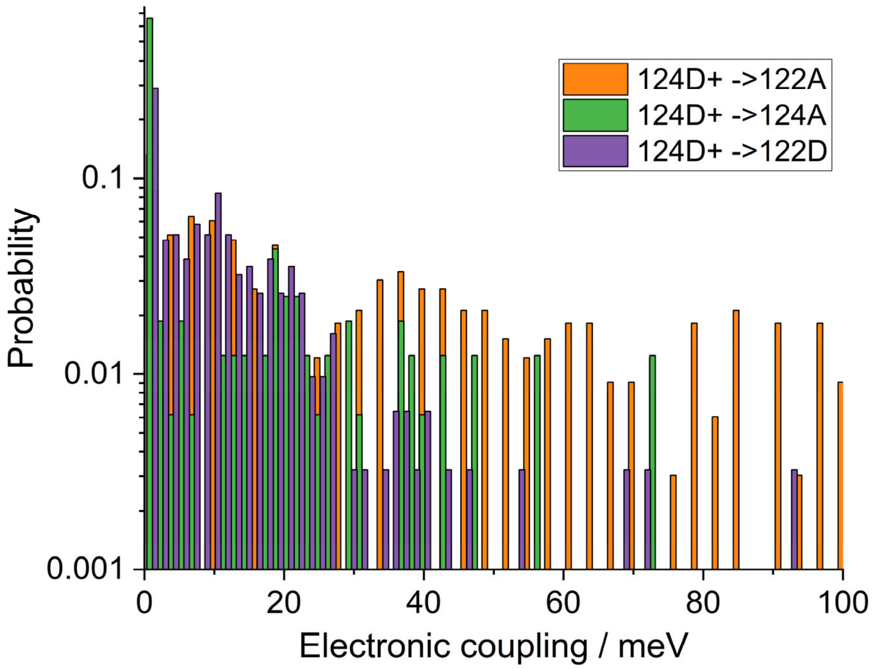

Figure S44. Distribution of H_{ab} values over the 0-100 meV range calculated for ET steps originating from 124D$^{\bullet+}$. (Probability is on a log scale.)

Table S2. Medians, averages, and standard average deviations of electronic coupling H_{ab} for hole transfer from oxidized indoles specified in the first column to neutral indoles in the first row.

	to 124A		to 122A		to 122D		to 124D		to **ReA**$^{-}$	
from	Med.	Ave.	Median	Average	Median	Average	Median	Average	Median	Average
$124A^{\bullet+}$	-	-	5	9±16	31	79±293	0.2	3±9	10	15±18
$122A^{\bullet+}$	14	18±22	-	-	11	36±123	18	42±147	0	-
$122D^{\bullet+}$	25	56±137	41	59±69	-	-	7	18±40	0	11±33
$124D^{\bullet+}$	0.1	8±15	31	51±80	8	11±18	-	-	0	-

S8. ΔG and H_{ab} summary

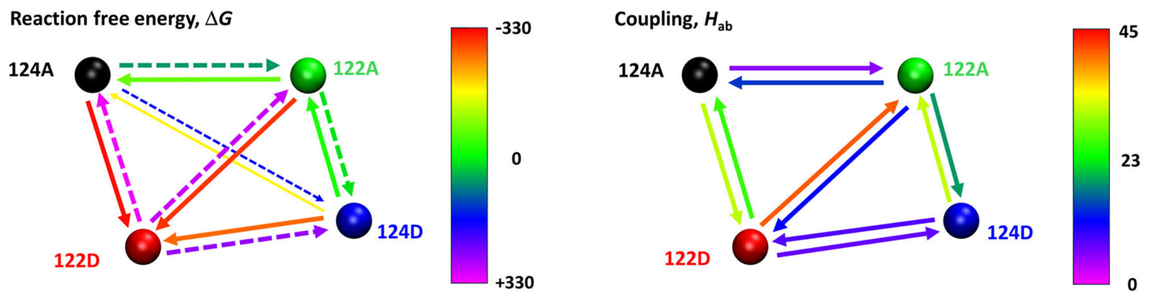

Scheme S1. Hole-transfer pathways within the tryptophan quadruplex, color-graded by the free energy change (left) and the median electronic coupling (right). All values are in meV. Dashed arrows in the left panel denote uphill steps.

S9. Computational details

The simulation protocol is summarized in the main text, Figure 2. In order to realistically simulate the evolution of excited states by QM/MM, we prepared the initial thermalized (300 K) geometries by mimicking the experimentally established mechanism (Scheme 1). We first performed 20 ns MM/MD *NpT* simulations with GS parameters to generate a set of independent geometries of the protein native state, sampling different sidechain conformations and solvent distributions. Next, we performed four 1 ns long MM/MD simulations with MLCT FF parameters starting from GS geometries and velocities at 5, 10, 15 and 20 ns. One 10 ns long MM/MD simulation with $124A^{\bullet+}$ and one with $122D^{\bullet+}$ FF parameters started at the end-structure of each MLCT trajectory (altogether providing four 10-ns trajectories of $124A^{\bullet+}$ and $122D^{\bullet+}$ each). Finally, FF parameters were switched at 1, 4, and 10 ns of each $124A^{\bullet+}$ and $122D^{\bullet+}$ trajectory to those of $122A^{\bullet+}$ and $124D^{\bullet+}$, respectively, and simulations continued for another 10 ns. Selected geometries and velocities of MM/MD simulations of individual states served as inputs for UKS DFT QM/MM/MD runs of the oxidized states. Structural parameters were monitored in all simulations. UKS calculations were used to obtain distributions of Mulliken charge and spin within active fragments of **$\{Re126W124W122Cu^{I}\}_2$**, electronic coupling, and electrostatic potentials. Structural parameters were monitored in all simulations.

S9.1. Classical MM/MD simulations

System preparation. As an initial geometry of the azurin dimer, the arrangement of A and D monomers from 6MJS PDB crystal structure were utilized. Missing amino acid residues on the ends of proteins were completed. Subsequently, hydrogen atoms were added into the structure using the *pdb4amber* module keeping H35 in the neutral protonation state in both monomers. Neutral charge of the system with both reduced Cu^{I} centers was reached by addition of 6 Na^{+} ions into the system. Finally, the dimer was solvated with 38289 water molecules forming a cubic simulation box of 11×11×11 nm dimensions.

Parametrization. The protein ground state was described using the Amber parm10 force field with the ff14SB modifications[2, 3] where the Cu center and the Re photosensitizer ($[Re(imidazole)(CO)_3(dmp)]^+$) were parametrized separately. Charge of the Cu^{I} cation was reduced to the 0.53 e based on the calculated CM5 charges due to a electron strong donation

from Cys112 ligand which also lead to reduction of negative charge of the S atom of Cys112 to 0.41 e. Cu^{I} ion position in the center was kept using five restraints on its distances from ligands. Force constants were 50 and 25 kcal·mol^{-1}·Å^{-2} for ligands in the equatorial plane (His46, Cys112, His117) and axial ligands (Gly45, Met121), respectively along with equilibrium distances: 2.95 Å for Cu-G45(O), 2.03 Å for Cu-H46(N), 2.18 Å for Cu-C112(S), 2.04 Å for Cu-H117(N), and 3.21 Å for Cu-M121(S) based on the crystallographic data.

In our previous study[4] we developed a unique set of bonded and nonbonded parameters for GS and MLCT state $[Re^{I}(imidazole)(CO)_3(dmp)]^+$ and recently, parameters for the reduced Re complex $[Re^{I}(imidazole)(CO)_3(dmp^-)]$ were prepared within the study of charge separated triplet states in the **Re126W124W122Cu$^{\mathbf{I}}$** monomer.[5] These sets of parameters were employed in simulations of individual electronic states of azurin mutant dimer when the Re photosensitizer in the D monomer remained in its GS.

Atomic charges in MM simulations of GS, MLCT, $124A^{\bullet+}$, $122A^{\bullet+}$, $122D^{\bullet+}$, and $124D^{\bullet+}$ states were modelled by changing the charge distribution on corresponding fragments within the QM part of the dimeric system. We started from the previously developed parametrization of the $124A^{\bullet+}$ state of the monomer.[4, 5] In each dimer state, atoms of the oxidized tryptophan and its adjacent tryptophan in the same molecule possessed charges of the oxidized W124 and neutral W122 in the $124A^{\bullet+}$-monomer reference parametrization, respectively. Atomic charges on the tryptophans in the second monomer corresponded to the original Amber parametrization (parm10 with ff14SB). In addition, equilibrium bond lengths in the oxidized tryptophan were modified to match bonds of a QM-optimized positively charged tryptophan. Such a parametrization provided stable electronic states in the subsequent QM/MM calculations.

The SPC/E model was used for explicit water surroundings[6] and six Na^+ cations (also from Amber FF) were added to neutralize the system.

MM/MD simulations. We carried out classical MM/MD simulations using the AMBER 18 software. Initially, the system was thermalized from 10 K to 300 K in 1 ns long *NVT* dynamics using Berendsen thermostat and correct density of the system was reached within 3 ns long NpT dynamics with Berendsen thermostat (300 K) and barostat (1 bar). Propagation runs were performed using Langevin dynamics (collision frequency of 10 ps^{-1}) in an *NpT* ensemble at an

ambient temperature and pressure (300 K, 1 bar) controlled by the Berendsen barostat. In all simulations, a 1 fs time-step was set and periodic boundary conditions were employed.[7] Cut-off distance of 9 Å was used for short-range non-bonding interactions, while long range electrostatics was accounted for by Ewald summation.

S9.2. QM/MM/MD simulations

The **$\{Re126W124W122Cu^I\}_2$** system was divided into QM (quantum) and MM (classical) parts as shown in Figure S45. The QM region was defined as $Re(CO)_3$(dmpA)(H126)L125W124G123W122$^+$ of the first molecule (A in PDB: 6MJS) and W124G123W122 of the second molecule (D in PDB: 6MJS). The QM part was terminated by linking-H-atoms that were attached to corresponding C_α atoms of the protein backbone. The rest of the system including $Re(CO)_3$(dmpD)(H126)$^+$ comprised the MM region. The protein construct was solvated in a water cap of 2500 SPC/E[6] water molecules, resulting in a minimum 15 Å water shell surrounding the QM part. Six Na^+ cations were added to compensate the charge of the protein.[8]

Molecular dynamics (MD) simulations of the **$\{Re126W124W122Cu^I\}_2$** in the four oxidized states were performed at the QM/MM level in Terachem 1.9[9, 10] – Amber 14[11] framework using electronic embedding. Description of the MM part remained unchanged and QM-part calculations utilized LANL2DZ quasi-relativistic effective core pseudopotentials and the corresponding optimized set of basis functions for Re[12] and 6-31g(d) polarized double - ζ basis sets[13] for the remaining atoms. Due to long and variable distances between indole fragments, DFT calculations employed the long-range-corrected functional CAM-B3LYP,[14] together with an empirical dispersion correction (D3).[15] Triplet states were calculated by the unrestricted KS procedure (UKS). Several testing calculations were performed with the hybrid functional PBE0[16, 17]. QM/MD simulations were performed with a 1 fs time step using the SHAKE algorithm.[18] Production runs of 500 fs (30 runs for each state, i.e., 120 in total) were performed at 300 K employing the Berendsen thermostat.[7]

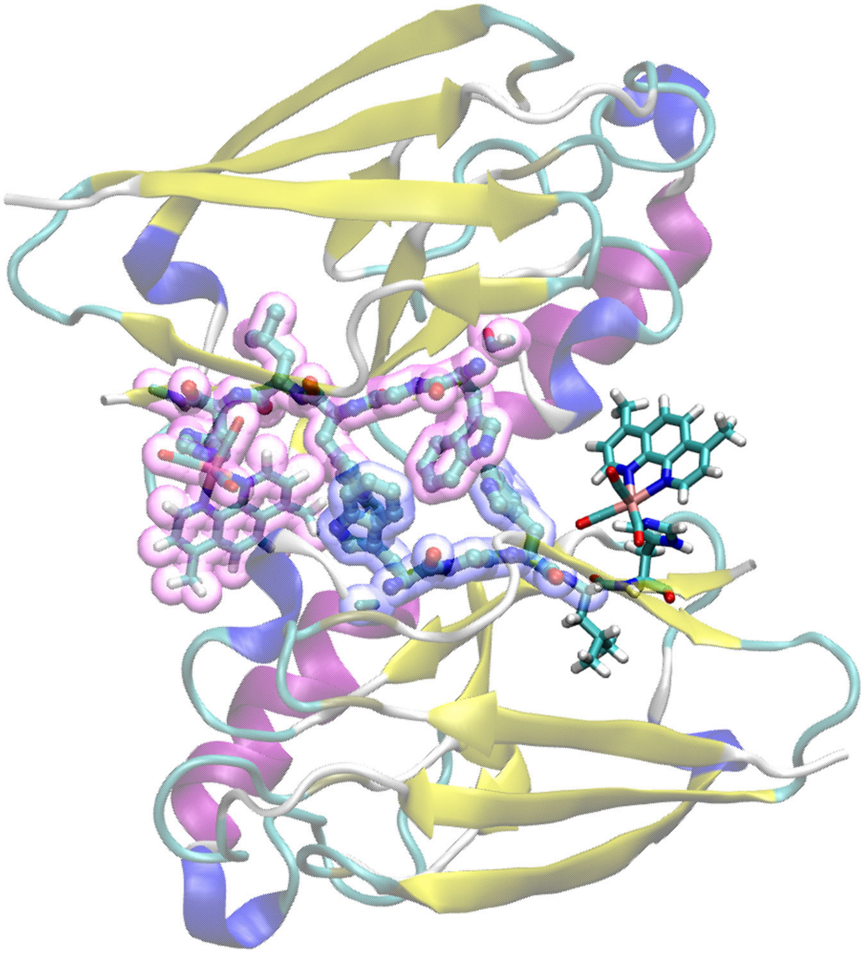

Figure S45. Structure of **{Re126W124W122Cu^{I}}$_2$**. The QM parts of molecules A and D are indicated by pink and light-blue surroundings. The rest of the system, including all water molecules and 6 Na^+ ions (to maintain electroneutrality), was treated classically.

S9.3. Electronic coupling and reaction energy calculations

Electronic couplings H_{ab} were approximated by calculating couplings between individual pairs of methylindole fragments in the Q-Chem 6.0 program package[19] using absolutely localized molecular orbitals (ALMO/DFT).[20, 21] Corresponding DFT calculations were performed at the CAM-B3LYP-D3(BJ)/6-31G(d) level. Effects of the rest of protein and solvent surroundings were included by adding electrostatic interactions with their atomic charges into the QM Hamiltonian. The target states were determined by fixing both the +1 e charge and the ½ spin on the oxidized methylindole, while the second methylindole was considered as a neutral singlet. Calculations were performed at 11 selected snapshots from the last 101 fs of each QM/MD trajectory (10 fs step between snapshots). First 400 fs of the QM/MD were not analyzed due to relaxation of the system.

Reaction energies were calculated based the Marcus theory. Assumed Gaussian statistics for the energy difference between the final and initial state $\Delta E = E_{fin} - E_{ini}$ leads the fact, that they can be determined as the average of mean values of ΔE for the system in its initial and final state $\Delta G_r = \frac{1}{2}\left(\langle \Delta E \rangle_{ini} + \langle \Delta E \rangle_{fin}\right)$.[1] (Angle brackets correspond to thermodynamic averaging in the initial and final state, respectively.)

S9.4. Electrostatic potentials

Electrostatic potentials *φ(r)* were determined based on atomic charges q_i from UKS DFT QM/MM dynamics and calculated at each given point as $\varphi_A(\boldsymbol{r}) = \sum_i \frac{q_i}{|\boldsymbol{r}_i - \boldsymbol{r}|}$, where the sum runs over contributing set of atoms *A* (can be solvent, protein, etc.). If such atoms belonged to the QM part, their actual Mulliken charges from UKS were employed, otherwise charges from the MM force field were used. For the investigated fragment, potentials were evaluated on a surface obtained as an envelope of scaled van der Waals spheres around its atoms.[5]. The scaling factor 0.5 was used. Points for potential calculations were generated based on Fibonacci lattices for the spheres. Finally, average potentials were determined as weighed averages of the potentials over the surface with weights corresponding to areas assigned to them based on the Delaunay triangulation. Atoms of the examined fragment never contributed to the potential. Calculations were performed using our Fortran code.

S9.5. Proximal volume *V*(*r*), coordination number *N*(*r*), and distribution function *g*(*r*). Spatial distribution function.

The proximal approach provides a quantitative and easy way to describe and interpret a solution structure (*e.g*. hydration) near non-spherical molecules.[22, 23] The calculation and interpretation of proximal distribution function (pRDF) is analogous to that of a radial distribution function and follows from $g_{\mathrm{prox}}(r)=\rho(r)/\rho_{\mathrm{bulk}}$. Knowledge of the proximal coordination number $N_{\mathrm{prox}}(r)$ of solvent molecules and proximal volume $V_{\mathrm{prox}}(r)$ is needed to determine the local density of the solvent $\rho(r)=\Delta N_{\mathrm{prox}}(r)/\Delta V_{\mathrm{prox}}(r)$, and thus its deviation from bulk density ρ_{bulk}. Calculation of $N_{\mathrm{prox}}(r)$ is straightforward, while $\Delta V_{\mathrm{prox}}(r)$ is computationally demanding, especially, as it requires that all the protein reminder (especially protein interior) is taken into account. For these reasons we use $V_{\mathrm{prox}}(r)$ only to quantify and illustrate the differences in accessibility of individual residues of the quantum domain between monomer and dimer (Figure 9).

Since this work aims at elucidating changes in hydration of tryptophan indols (or indol NH-groups), we focus on $N_{\mathrm{prox}}(r)$, which provides its robust measure.

The proximal distance is the closest distance measured between a water molecule and the solute (or a solute functional group). In the proximal sense, the hydration layer of a thickness *r* faithfully follows the shape ('molecular surface') of the molecule. The proximity criteria can be used for the entire protein molecule or for selected functional groups (e.g., amino acid residues, sidechains, …). This makes pRDF particularly convenient for proteins, flexible polymers, or complex molecules that consist of numerous functional groups. [22, 23] So called exclusive approach is employed in this work, where each water molecule is accounted only to the closest group. This avoids double-counting of water molecules, which is otherwise present in conventional RDF calculations. Importantly, it allows to evaluate hydration of larger segments (e.g., all TRP indols) by straightforward summation.

Spatial distribution function (SDF): Due to slow dynamics of the residues in the QM-domain and owing to confinements and sterical constraints, it was possible to calculate SDF of water in the proximity of the QM-domain. Trajectories from ensembles (for individual states) were best aligned (in root-mean-square-deviation) to a universal reference configuration, their SDFs

calculated and then ensemble-averaged SDF evaluated. This approach allowed us to extract the locations of most tightly coordinated water molecules, as well as to filter out the random hydration (noise). For clarity reasons (and purpose of this work), SDFs are visualized only in the vicinity (<6 Å), of the QM-domain (or the indoles). Using very fine spatial resolution (up to 0.1 Å), even small spatial shifts in hydration associated with a change of electronic state can be captured (see Figure S22).

For practical reasons, we applied a Gaussian smoothening in the SDF calculation, where each water oxygen atom contributed, not as a point, but as a Gaussian of a width (sigma), which was set to the bin size. Each oxygen atom thus dominantly contributes to the bin (e.g., 0.1 Å x 0.1 Å x 0.1Å), where it is positioned, but to a lesser extend also to neighboring bins (up to ca 3×sigma ~ ca 6^3=216 bins), keeping the proper normalization. This allowed us to keep very fine spatial resolution in SDF (0.1 Å) and simultaneously low noise in SDF, which are both necessary to capture shifts in hydration upon the change of electronic state.

We note that this approach was earlier established for determination of smooth intrinsic interfaces from limited and coarse data.[24] In our case, this method can be viewed/justified as an effective approach, which accounts for random motions of mapped particles on short (fs) time scales (short time diffusion, thermal fluctuations), without the need of storing the excessive trajectory data.

Analyses were performed using our in-house Python implementation of SDF and pRDF on system configurations from MM/MD simulation, which were saved every 0.1 ps, yielding approximately 2000 samples for analysis over a 200 ps simulation. The 200 ps length was long enough to sample water (re)distribution upon change of the electronic state (force-field), yet significant conformational changes in QM domain were limited (dmp-indole and indole-indole distances).

In order to receive meaningful and interpretable results the ensemble averages of QM-domain hydration (SDF, N_{prox}, g_{prox}) were always performed over structurally similar subsets of conformational ensembles of QM-domain (see e.g., Figure S21). It should be noted that simple averaging over whole (unbiased) ensembles does not provide meaningful, interpretable, or comparable results on changes in hydration of QM-domain between electronic states (see 1D representation in Figures S19, S20).

In case when differences in hydration between electronic states were compared, trajectories (in the investigated electronic states) started from the same initial condition (taken from the 124A$^{•+}$ state). Moreover, in line with the above text, only structurally similar configurations of QM-domain were used as initial conditions. The initial conditions were uncorrelated, as they were taken from very different simulation times in the 124A$^{•+}$ trajectory (separated by at least 100 ps), and thus the ensemble of initial conditions differed mainly in the initial distribution (positions) of water molecules. Such constructed trajectory ensembles were used to calculate average spatially-resolved indole hydration in individual states and changes in hydration between the examined states.

On a practical side, ensemble for each electronic state consisted of 27 uncorrelated trajectories (each 200 ps long, providing 2000 frames for analysis), thus in total 54 000 frames were used to construct 3D-spatial and 1D-proximal distribution of water in the proximity of QM-domain.

References

1. Matyushov, D. V., Reorganization energy of electron transfer. *Phys. Chem. Chem. Phys.* **2023,** *25*, 7589–7610.
2. Maier, J. A.; Martinez, C.; Kasavajhala, K.; Wickstrom, L.; Hauser, K. E.; Simmerling, C., ff14SB: Improving the Accuracy of Protein Side Chain and Backbone Parameters from ff99SB. *J. Chem. Theory Comput.* **2015,** *11*, 3696–3713.
3. Ponder, J. W.; Case, D. A., Force Fields for Protein Simulations. *Adv. Prot. Chem. 66,* **2003,** *66*, 27-85.
4. Záliš, S.; Heyda, J.; Šebesta, F.; Winkler, J. R.; Gray, H. B.; Vlček, A., Photoinduced hole hopping through tryptophans in proteins. *Proc. Natl. Acad. Sci. U.S.A.* **2021,** *118*, 5775−5785.
5. Melčák, M.; Šebesta, F.; Heyda, J.; Gray, H. B.; Záliš, S.; Vlček, A., Tryptophan to Tryptophan Hole Hopping in an Azurin Construct. *J. Phys. Chem. B* **2024,** *128*, 96−108.
6. Berendsen, H. J. C.; Grigera, J. R.; Straatsma, T. P., The Missing Term in Effective Pair Potentials. *J. Phys. Chem.* **1987,** *91*, 6269-6271.
7. Berendsen, H. J. C.; Postma, J. P. M.; van Gunsteren, W. F.; DiNola, A.; Haak, J. R., Molecular dynamics with coupling to an external bath. *J. Chem. Phys. 81,* **1984,** *81*, 3684-3690.
8. Heyda, J.; Pokorna, J.; Vrbka, L.; Vacha, R.; Jagoda-Cwiklik, B.; Konvalinka, J.; Jungwirth, P.; Vondrasek, J., Ion Specific Effects of Sodium and Potassium on the Catalytic Activity of HIV-1 Protease. *Phys. Chem. Chem. Phys.* **2009,** *11*, 7599−7604.
9. Ufimtsev, I. S.; Martínez, T. J., Quantum Chemistry on Graphical Processing Units. 3. Analytical Energy Gradients and First Principles Molecular Dynamics. *J. Chem. Theor. Comp.* **2009,** *5*, 2619-2628.
10. Titov, A. V.; Ufimtsev, I. S.; Luehr, N.; Martínez, T. J., Generating Efficient Quantum Chemistry Codes for Novel Architectures. *J. Chem. Theor. Comp.* **2013,** *9*, 213-221.

11. Case, D. A.; Berryman, J. T.; Betz, R. M.; Cerutti, D. S.; III, T. E. C.; Darden, T. A.; Duke, R. E.; Giese, T. J.; Gohlke, H.; Goetz, A. W.; Homeyer, N.; Izadi, S.; Janowski, P.; Kaus, J.; Kovalenko, A.; Lee, T. S.; LeGrand, S.; Li, P.; Luchko, T.; Luo, R.; Madej, B.; Merz, K. M.; Monard, G.; Needham, P.; Nguyen, H.; Nguyen, H. T.; Omelyan, I.; Onufriev, A.; Roe, D. R.; Roitberg, A.; Salomon-Ferrer, R.; Simmerling, C. L.; Smith, W.; Swails, J.; Walker, R. C.; Wang, J.; Wolf, R. M.; Wu, X.; York, D. M.; Kollman, P. A. *AMBER 2014*, University of California: San Francisco, 2015.

12. Hay, P. J.; Wadt, W. R., Ab initio effective core potentials for molecular calculations – potentials for K to Au including the outermost core orbitals. *J. Chem. Phys.,* **1985,** *82*, 299-310.

13. Hehre, W. J.; Ditchfield, R.; Pople, J. A., Self—Consistent Molecular Orbital Methods. XII. Further Extensions of Gaussian—Type Basis Sets for Use in Molecular Orbital Studies of Organic Molecules. *J. Chem. Phys.* **1972,** *56*, 2257-2261.

14. Yanai, T.; Tew, D. P.; Handy, N. C., A new hybrid exchange-correlation functional using the Coulomb-attenuating method (CAM-B3LYP). *Chem. Phys. Lett.* **2004,** *393*, 51-57.

15. Grimme, S.; Antony, J.; Ehrlich, S.; Krieg, H., A consistent and accurate ab initio parametrization of density functional dispersion correction (DFT-D) for the 94 elements H-Pu. *J. Chem. Phys.* **2010,** *132*, 154104.

16. Adamo, C.; Barone, V., Toward reliable density functional methods without adjustable parameters: The PBE0 model. *J. Chem. Phys.* **1999,** *110*, 6158-6170.

17. Adamo, C.; Scuseria, G. E.; Barone, V., Accurate excitation energies from time-dependent density functionl theory: Assessing the PBE0 model. *J. Chem. Phys.* **1999,** *111*, 2889-2899.

18. Ryckaert, J. P.; Ciccotti, G.; Berendsen, H. J. C., Numerical-Integration of Cartesian Equations of Motion of a System with Constraints - Molecular-Dynamics of N-Alkanes. *J. Comput. Phys.* **1977,** *23*, 327−341.

19. Epifanovsky, E.; Gilbert, A. T. B.; Feng, X.; Lee, J.; Mao, Y.; Mardirossian, N.; Pokhilko, P.; White, A. F.; Coons, M. P.; Dempwolff, A. L., et al., Software for the frontiers of quantum chemistry: An overview of developments in the Q-Chem 5 package. *J. Chem. Phys.* **2021,** *155*, 084801.

20. Mao, Y.; Montoya-Castillo, A.; Markland, T. E., Excited state diabatization on the cheap using DFT: Photoinduced electron and hole transfer. *J. Chem. Phys.* **2020,** *153*, 244111.

21. Voityuk, A. A.; Rösch, N., Fragment charge difference method for estimating donor–acceptor electronic coupling: Application to DNA π-stacks. *J. Chem. Phys.* **2002,** *117*, 5607-5516.

22. Polák, J.; Ondo, D.; Heyda, J., Thermodynamics of N-Isopropylacrylamide in Water: Insight from Experiments, Simulations, and Kirkwood−Buff Analysis Teamwork. *J. Phys. Chem. B* **2020,** *124*, 2495−2504.

23. Lin, B.; Pettitt, B. M., On the universality of proximal radial distribution functions of proteins. *J. Chem. Phys.* **2011,** *134*, 106101

24. Willard, A. P.; Chandler, D., Instantaneous Liquid Interfaces. *J. Phys. Chem. B* **2010,** *114*, 1954–1958.
