# Supplementary material for: Electron Transport through a Tryptophan Quadruplex in a Dimeric Azurin Construct
Source: J Phys Chem B. 2026 Jan 21;130(5):1503–14. doi: 10.1021/acs.jpcb.5c06932 (PMC12884451; doi:10.1021/acs.jpcb.5c06932)
Supplement: Supplementary file 2 [file jp5c06932_si_002.pdf]

Supporting Information 2

Electron Transport through a Tryptophan Quadruplex in a Dimeric Azurin Construct

Martin Melčák,[a,b] Jan Heyda,[a,b] Filip Šebesta,[c,a] Harry B. Gray,*[,d] Stanislav Záliš,*[,a] Antonín Vlček*[,a,e]

[a] J. Heyrovský Institute of Physical Chemistry, Czech Academy of Sciences, Dolejškova 3, CZ-182 23 Prague, Czech Republic

[b] Department of Physical Chemistry, University of Chemistry and Technology Prague, Technická 5, CZ-166 28 Prague, Czech Republic

[c] Department of Chemical Physics and Optics, Faculty of Mathematics and Physics, Charles University, Ke Karlovu 3, CZ-121 16 Prague, Czech Republic

[d] Beckman Institute, California Institute of Technology, Pasadena, California 91125, United States

[e] Department of Chemistry, Queen Mary University of London, E1 4NS London, U.K.

Figure S2.1.

Selected crystal structures of interfacial tryptophan quadruplexes in EC-1 oxidoreductases.

Criteria for structures shown on pages 2-6:
- 2 tryptophan residues in each monomeric unit
- One shortest indole-indole distance is longer than 5.1 Å, all other distances are shorter.

Structures are identified by their PDB codes.
Interfacial tryptophans are shown in protein structures in red and blue.
Quadruplex structures are also shown separately together with selected distances.
Color coding corresponds to tryptophan numbering in Table S2:
1 - Blue
2 - Red
3 - Yellow
4 – Green

3O0K

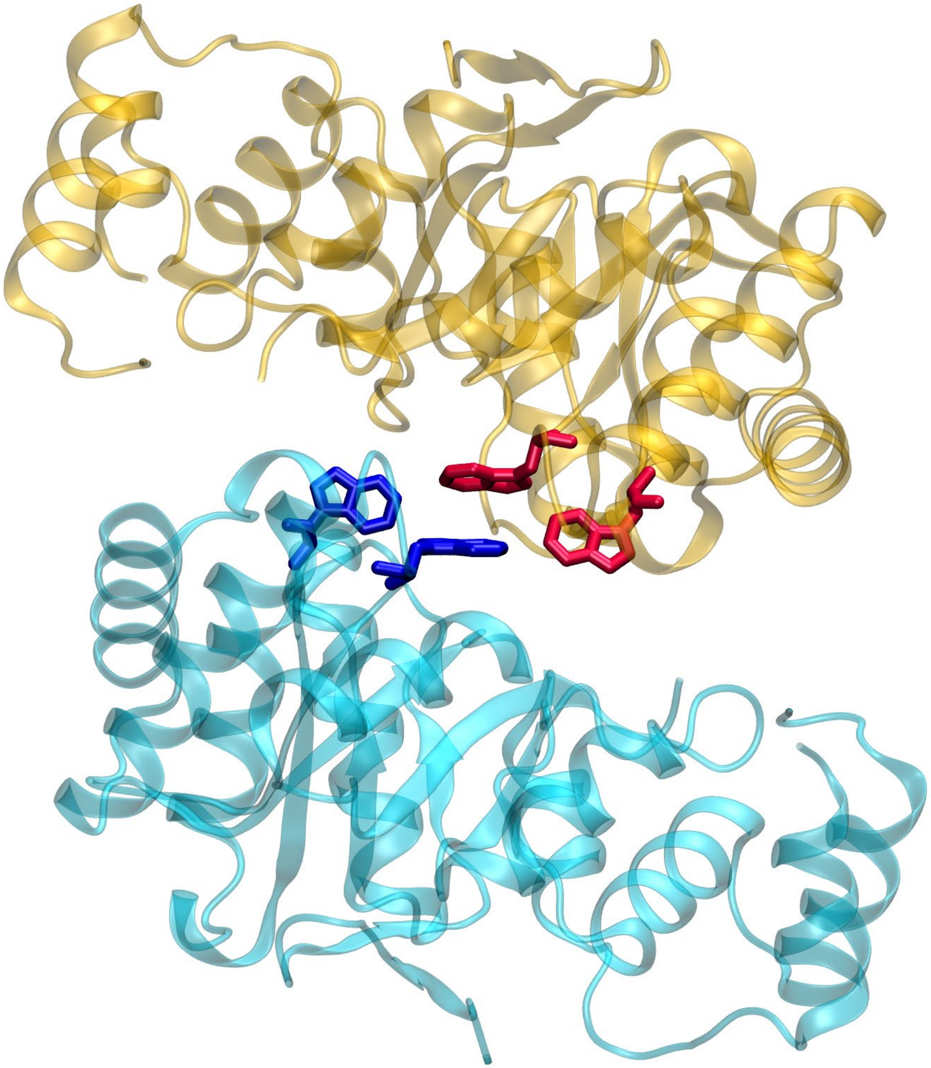

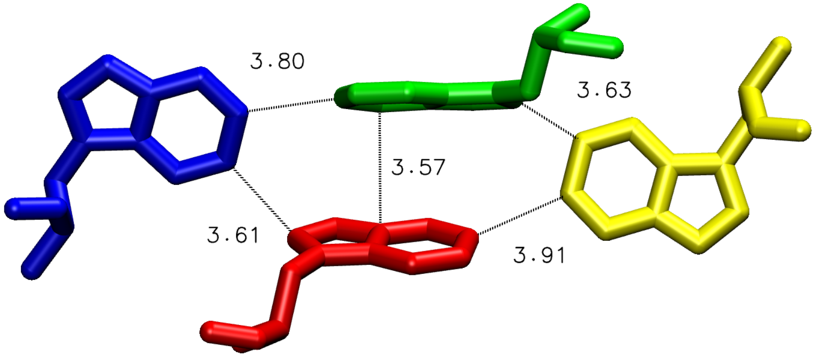

5YBL

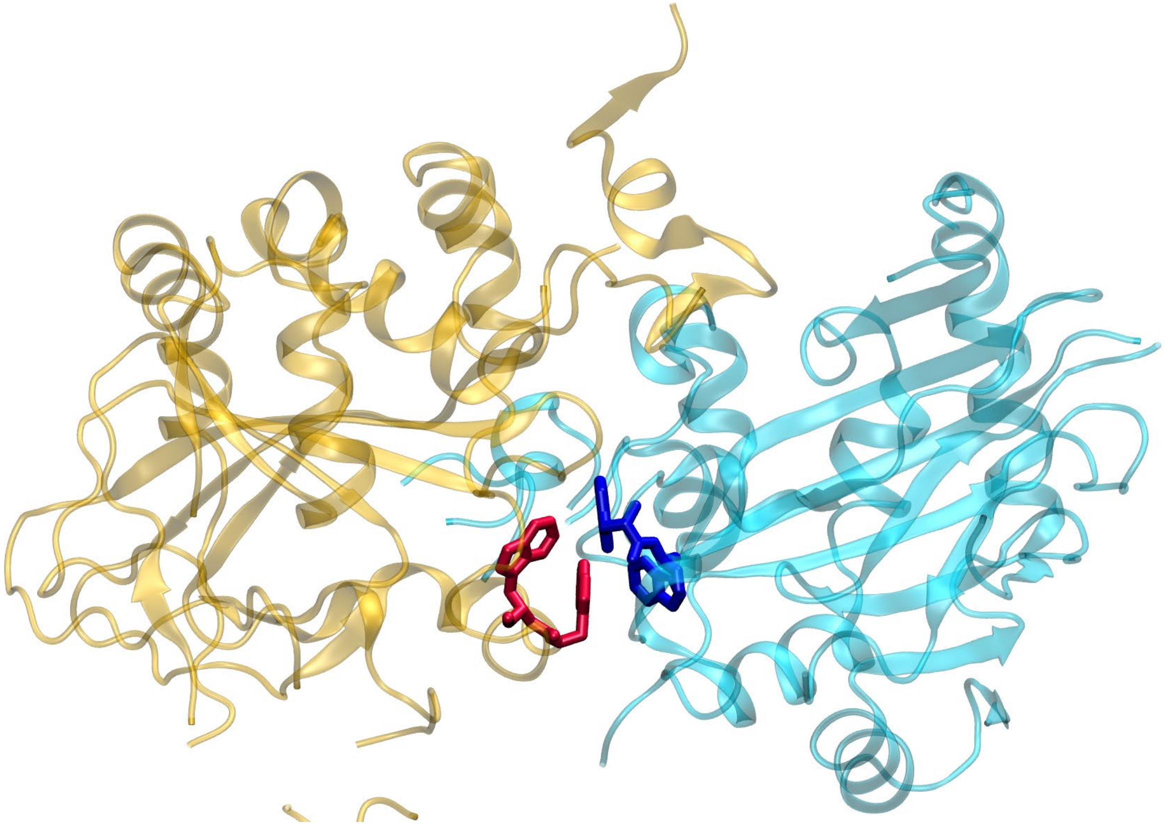

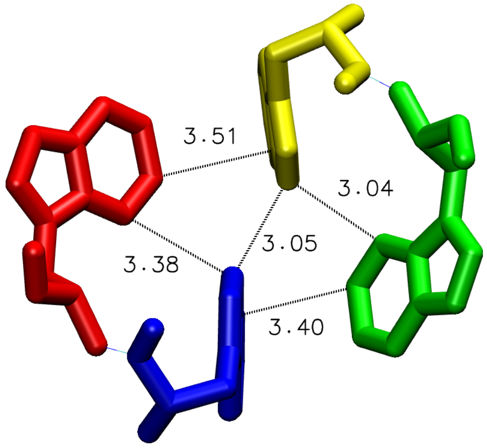

5YBN

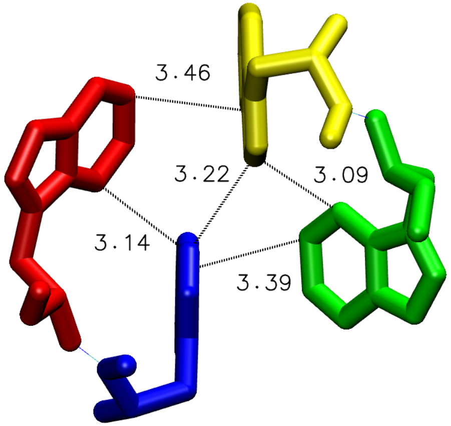

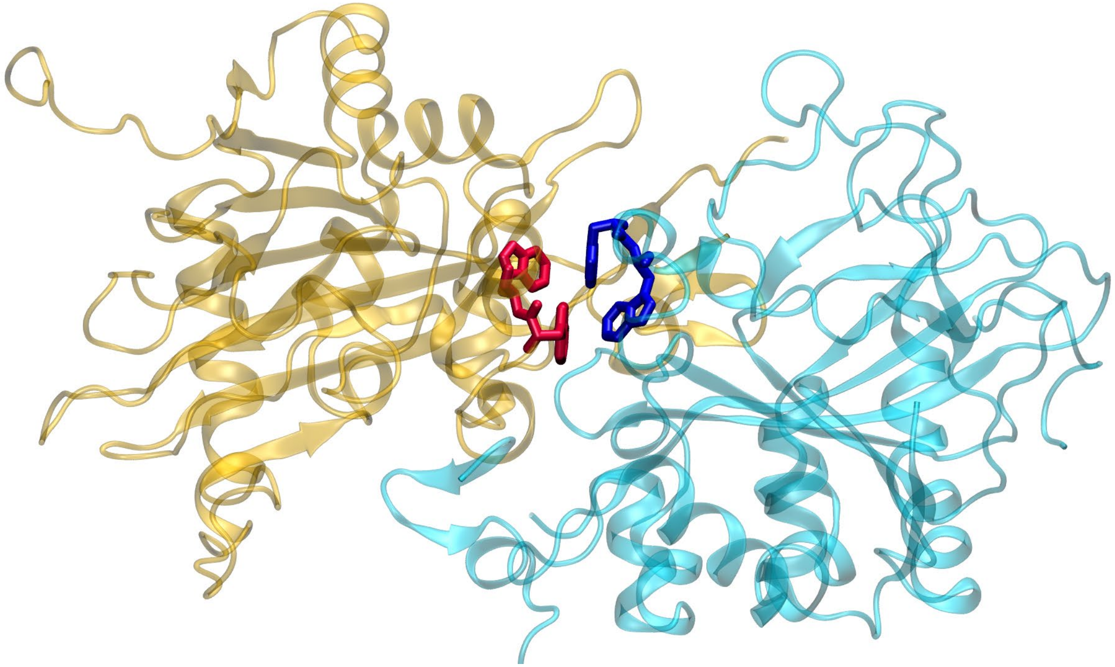

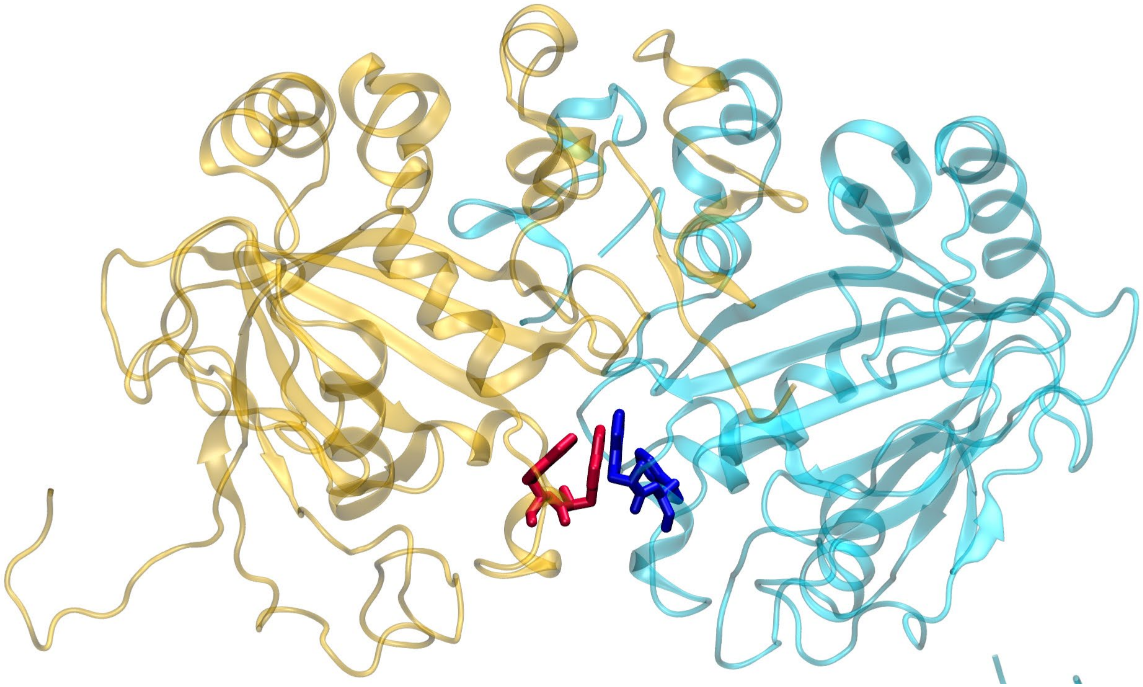

6LKC

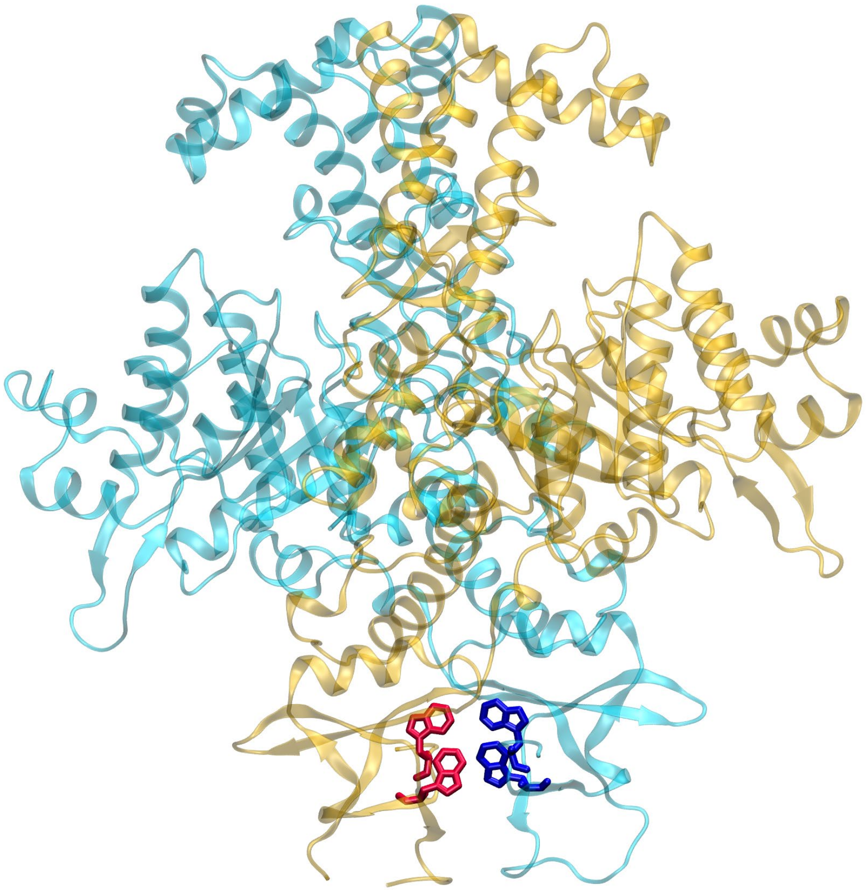

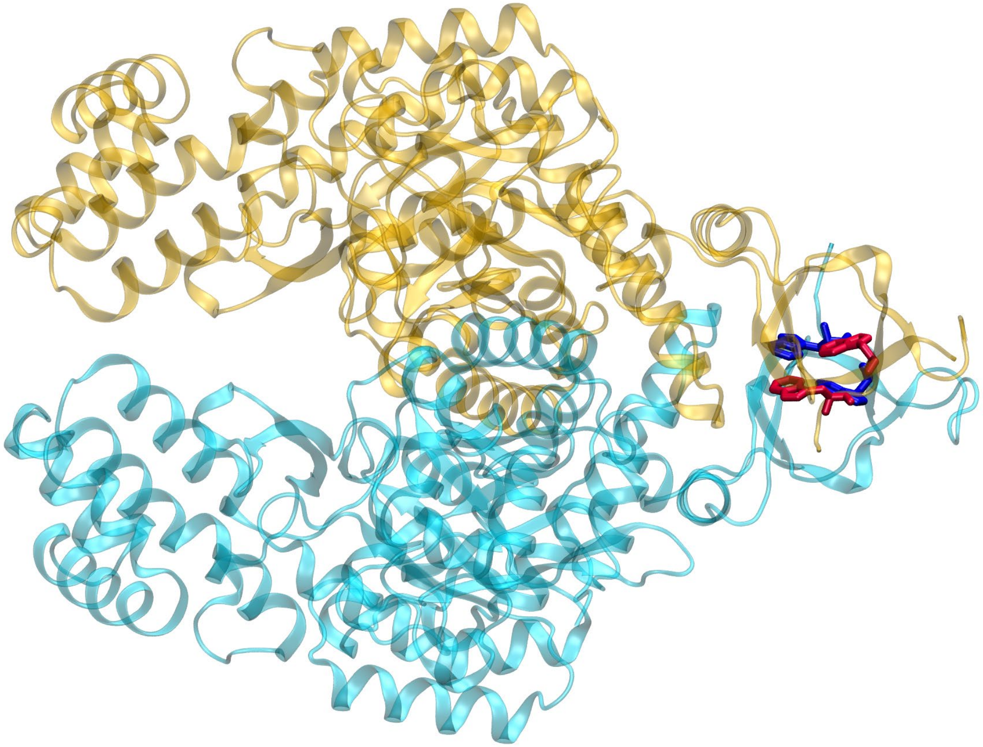

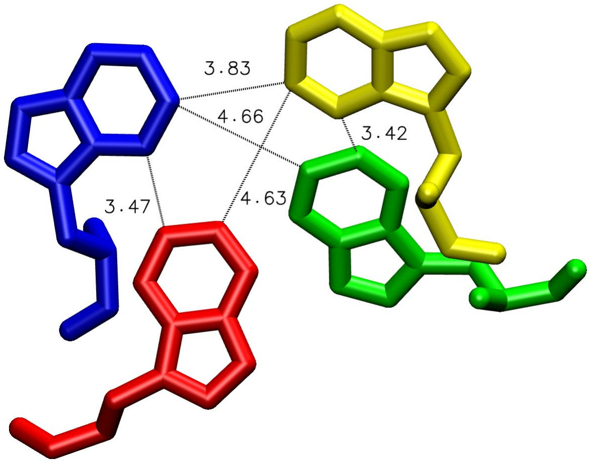

7EUU

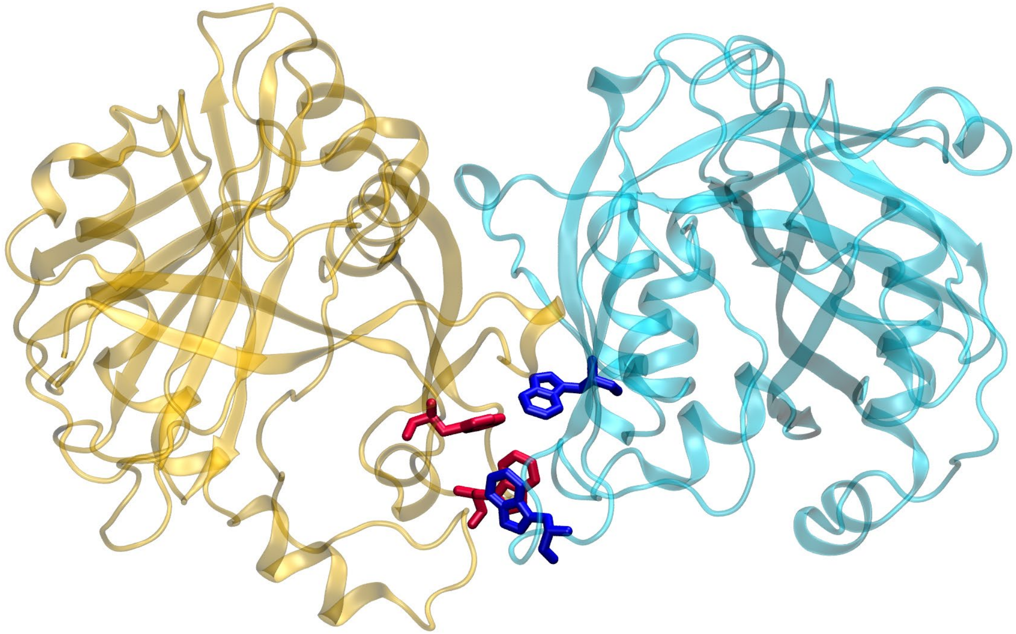

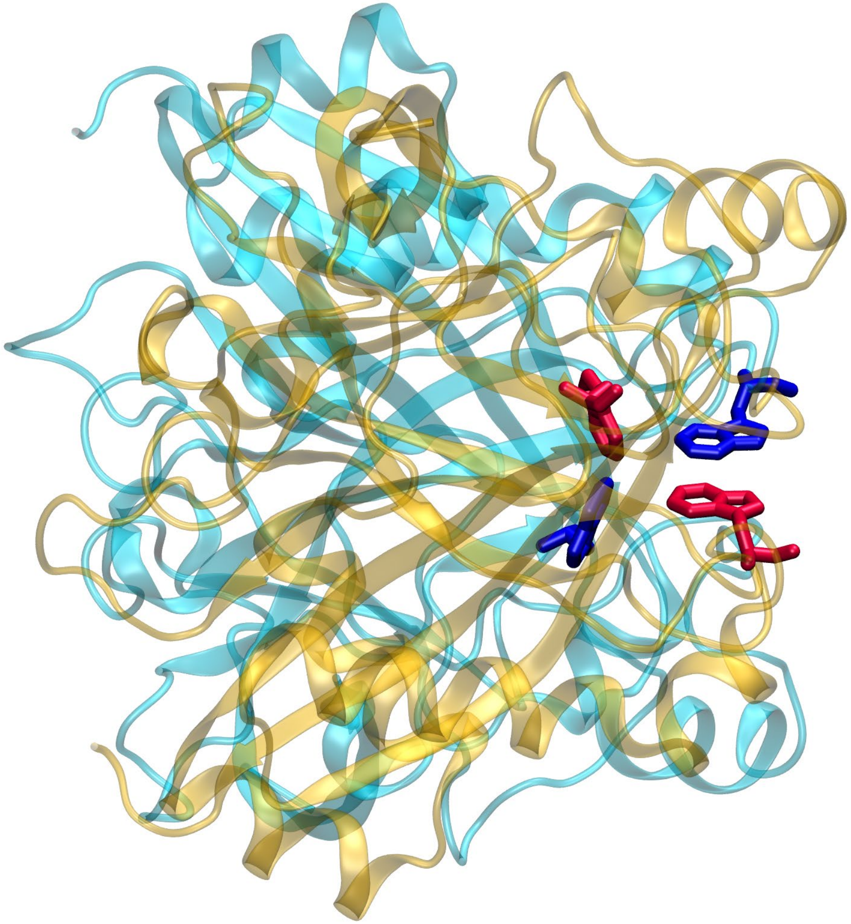

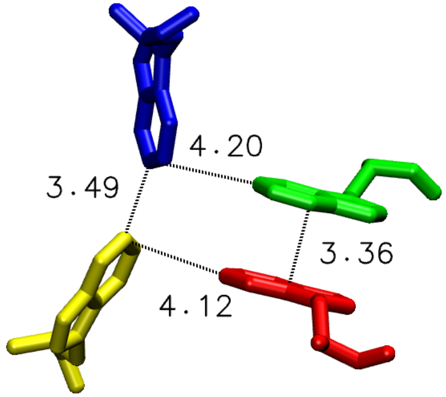

Figure S2.2.

Selected crystal structures of interfacial tryptophan quadruplexes in EC-1 oxidoreductases.

Criteria for structures shown on pages 8-12:
- 2 tryptophan residues in each monomeric unit
- Two shortest indole-indole distance are longer than 6.0 Å, all other distances are shorter.

Structures are identified by their PDB codes.
Interfacial tryptophans are shown in protein structures in red and blue.
Quadruplex structures are also shown separately together with selected distances.
Color coding corresponds to tryptophan numbering in Table S2:
1 - Blue
2 - Red
3 - Yellow
4 – Green

4I8P

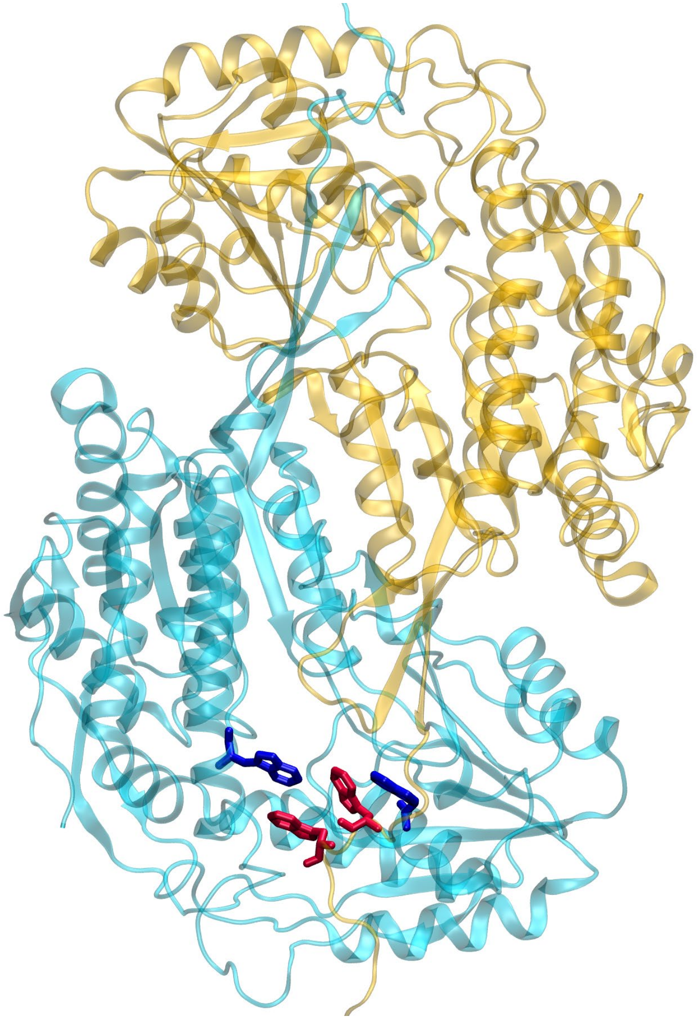

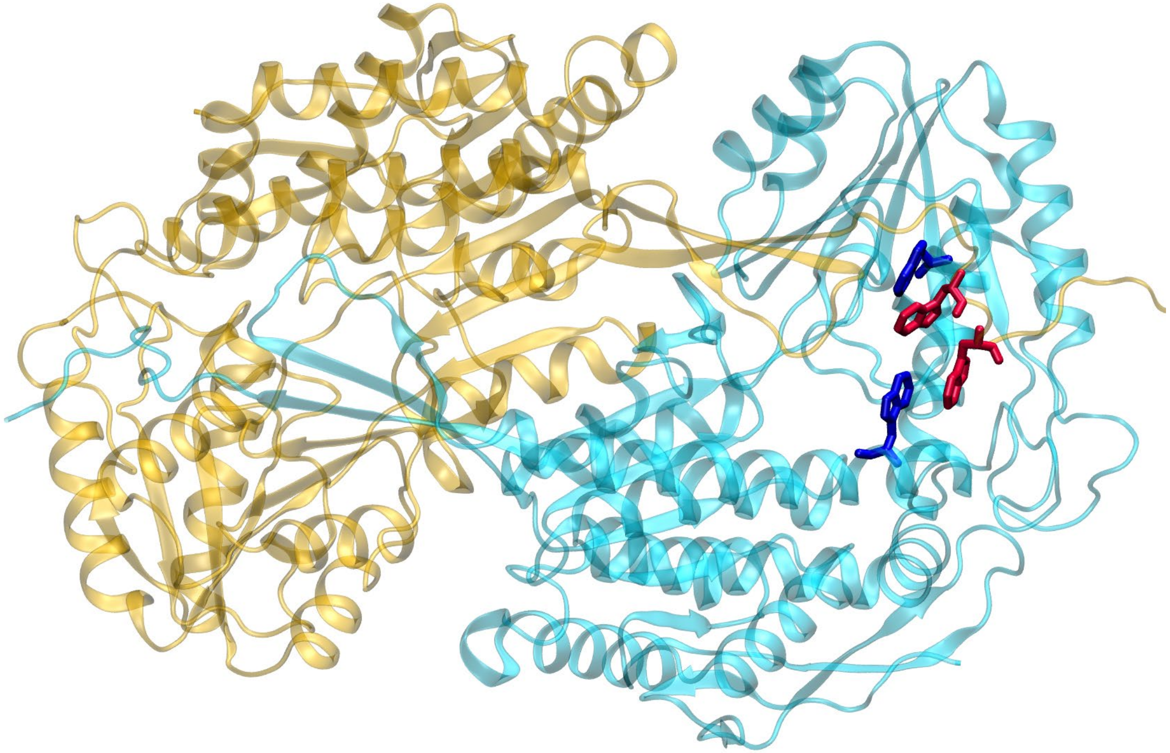

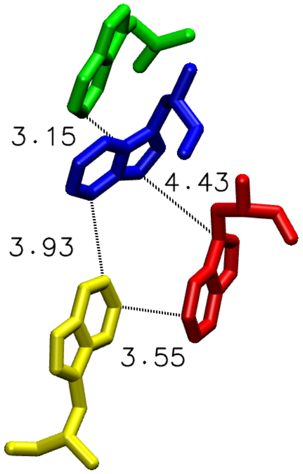

5A2D

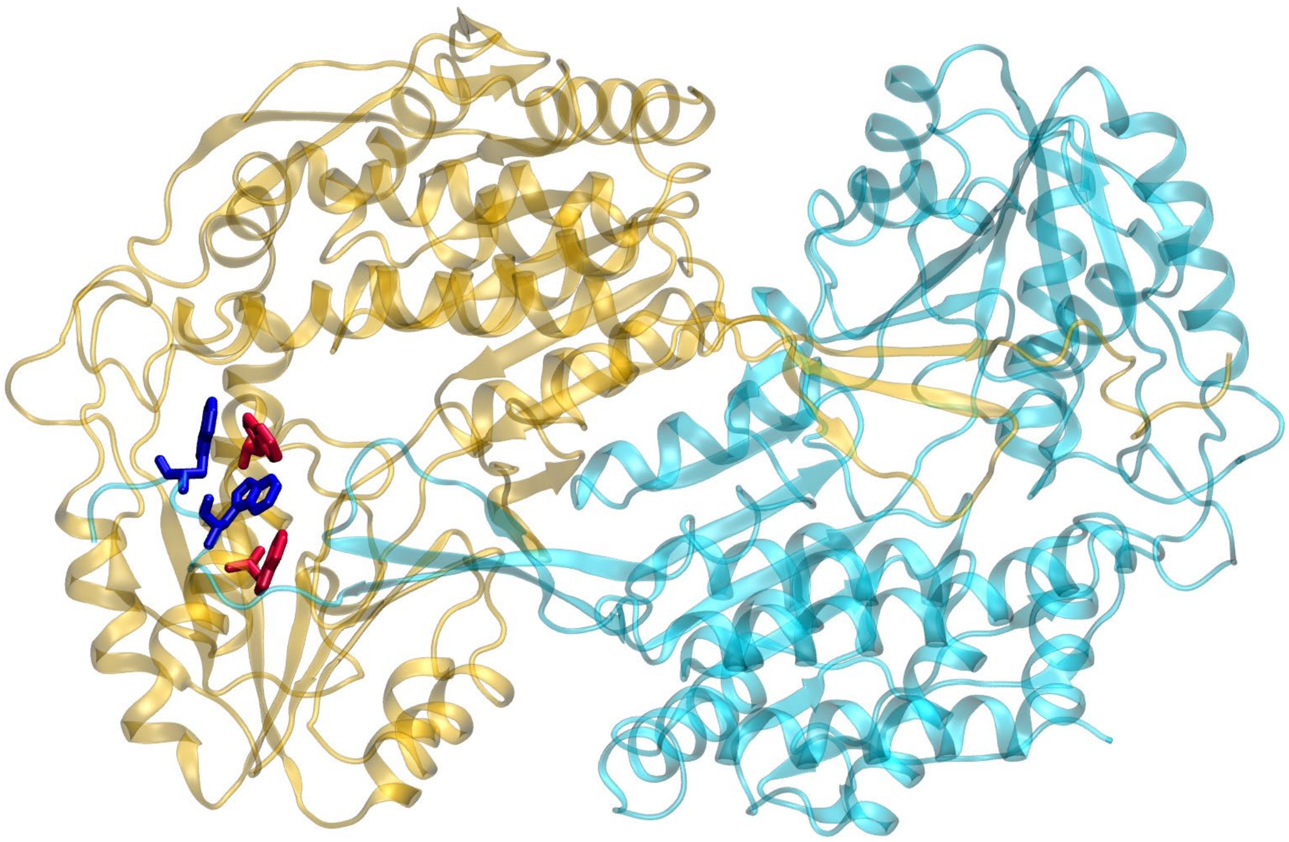

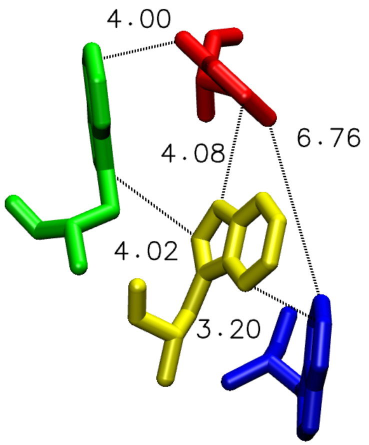

6IXJ

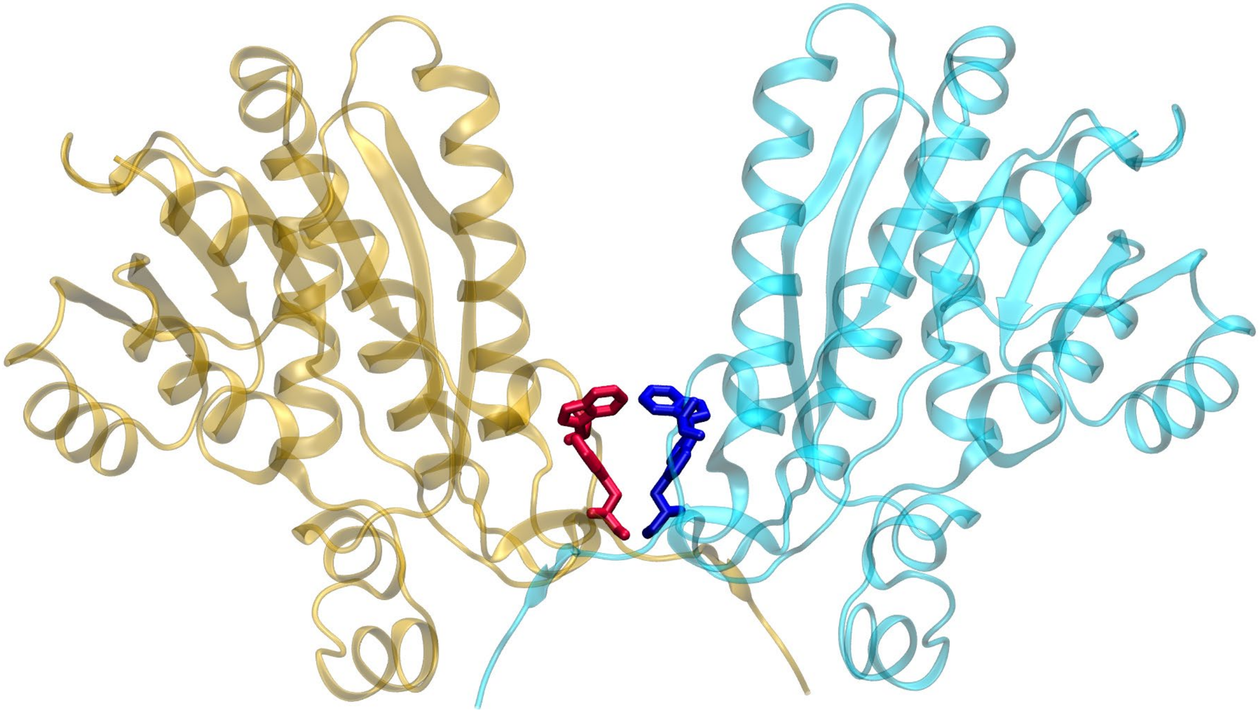

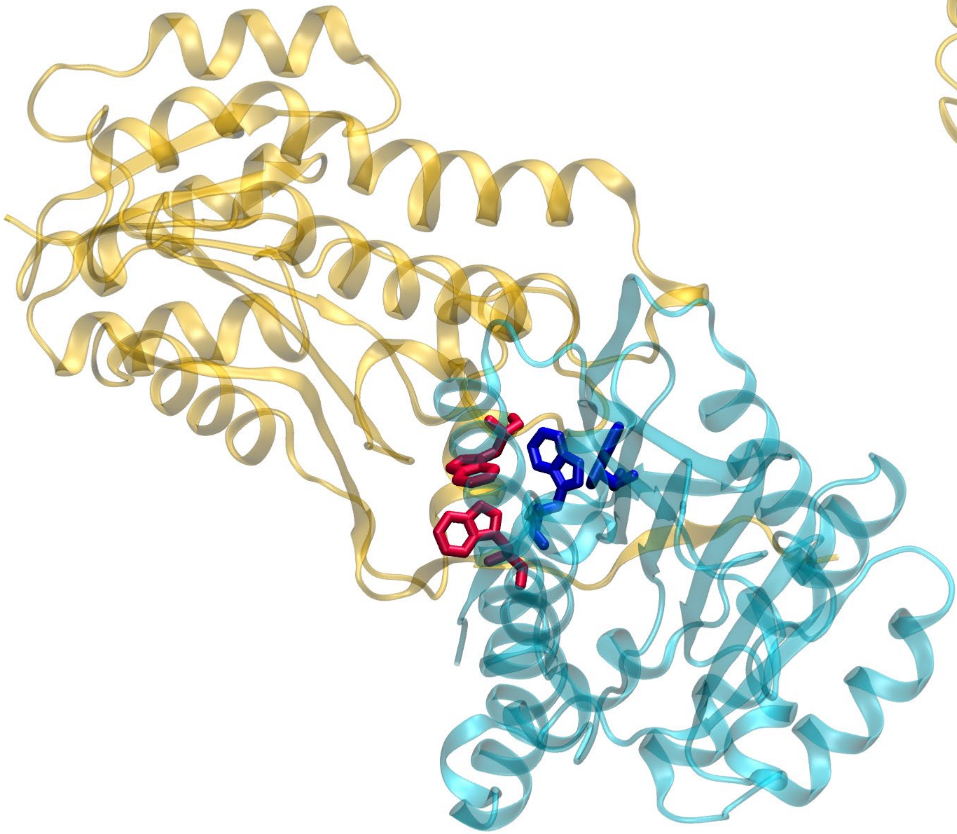

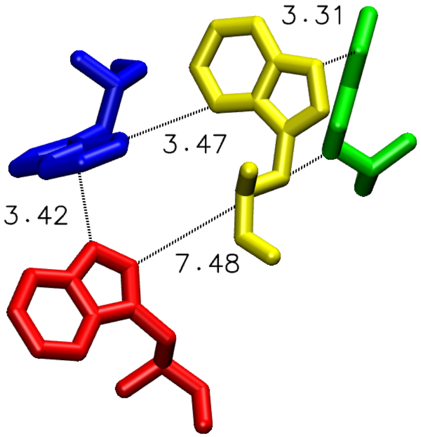

7CL2

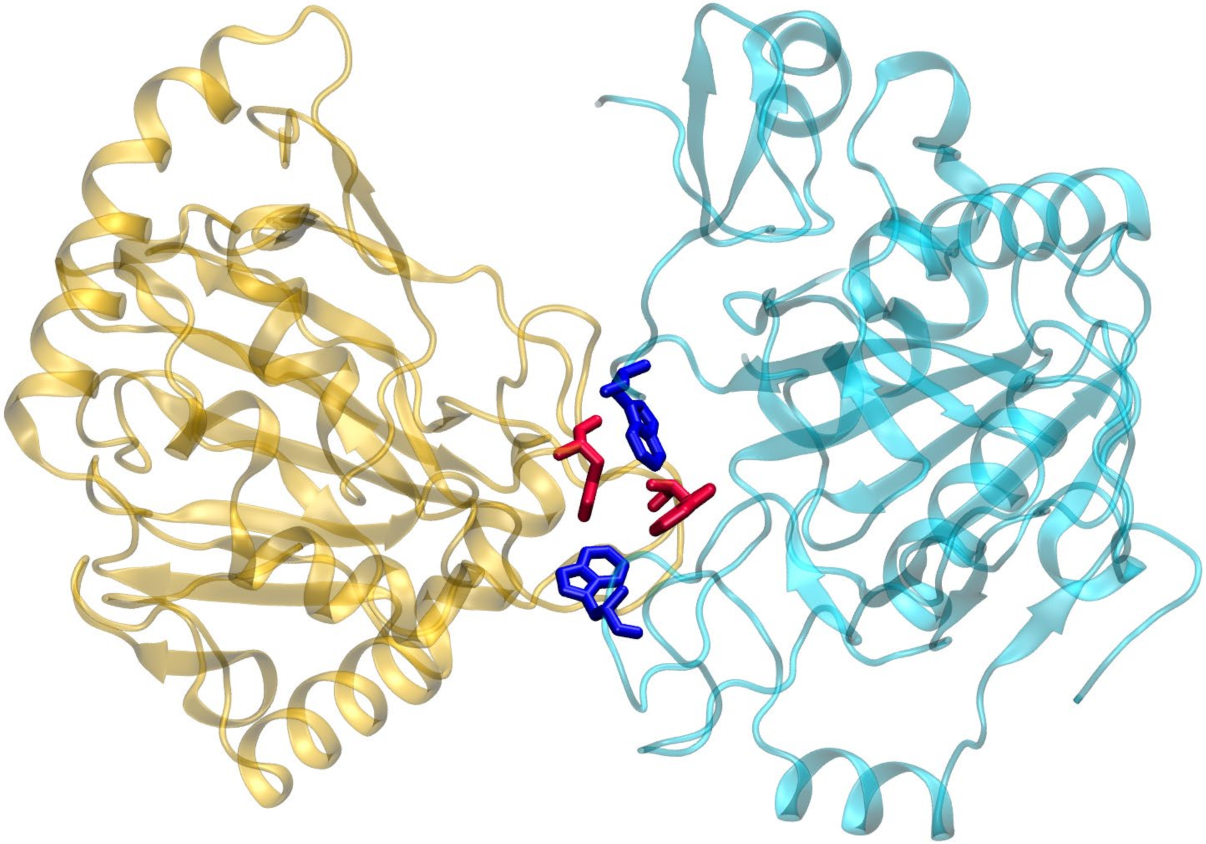

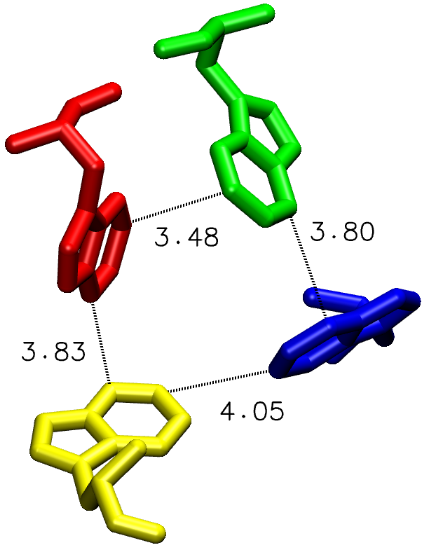

8JFJ

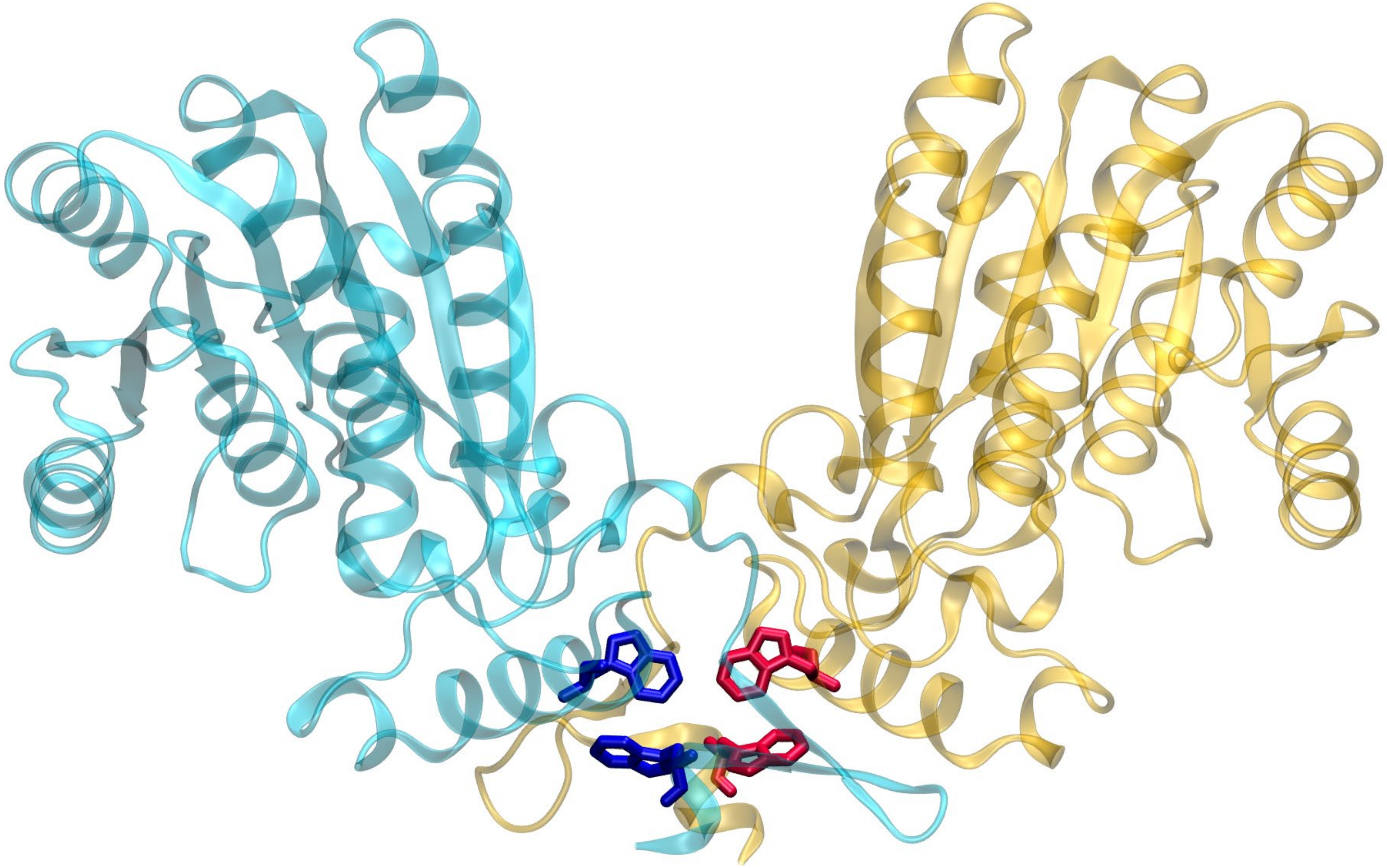

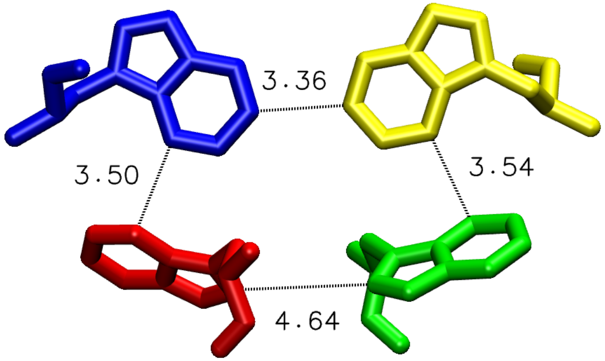

Table S2.1. List of tryptophan quadruplexes in EC-1 oxidoreductases.
Left column: Volume of the ellipsoid surrounding the quadruplex.
Right column: PDB code followed by a list of tryptophans forming the quadruplex.
(The first two entries 6MJS correspond to two pairs present in the $\{Re126W124W122Cu^{II}\}_2$ unit cell.)

VOLUME	P DB-ID	RESIDUES			
	6MJS	A 122	A 124	D 122	D 124
	6MJS	B 122	B 124	C 122	C 124
388.3	8DQ8	A 111	A 117	B 171	B 240
389.2	8JFJ	A 208	A 269	C 208	C 269
397.0	5SXW	A 139	A 202	A 208	B 42
397.9	8U3P	A 38	D 135	D 198	D 204
408.0	5WHS	A 118	A 182	A 188	B 21
438.3	4PHZ	K 47	K 132	I 132	J 116
447.5	6AQZ	A 94	A 163	B 94	B 163
453.7	4I8P	A 496	A 498	B 111	B 283
453.7	2WEU	B 205	B 377	C 205	C 377
481.1	1YQ9	B 84	I 327	I 345	I 356
494.0	5Z76	A 92	A 154	C 92	C 154
498.3	4DGQ	A 185	B 184	B 185	C 185
499.6	6FCX	A 421	A 455	A 591	A 595
506.5	3IWJ	A 494	A 496	B 109	B 317
523.4	8AEP	B 884	B 889	B 915	B 1046
523.6	3O0K	B 96	B 128	C 96	C 128
530.6	6IXJ	B 146	B 246	H 146	H 246
538.6	2XTS	A 208	A 258	A 298	A 300
538.7	5L6G	A 243	A 253	A 364	A 432
557.7	2QJY	D 313	D 391	G 313	G 391
560.8	2YY7	A 91	A 153	B 91	B 153
567.0	5A2D	C 278	C 285	D 491	D 493
574.5	1H0H	A 458	A 459	A 693	A 775
577.7	1A88	A 184	B 183	B 184	C 184
584.6	3L9W	A 1061	A 1077	A 1102	A 1139
588.3	2OQ2	C 20	C 36	D 20	D 36
593.5	7CL2	A 139	A 241	B 139	B 241
594.6	4J6V	A 41	A 269	B 41	B 269
598.4	2Q17	A 114	A 196	C 114	C 115
599.9	2IVF	A 72	A 74	A 104	B 42
617.0	7U9U	B 52	B 107	B 132	B 320
623.2	6QGR	A 252	A 257	A 373	A 396
624.9	5YBN	A 138	A 139	B 138	B 139
629.5	8QDQ	A 635	A 710	A 726	A 727
634.8	4XYB	A 178	B 100	B 311	B 322
635.3	5XBR	A 208	A 209	B 81	B 84
635.5	2BS2	C 28	C 29	C 194	C 196
638.5	3PF7	A 27	A 35	A 74	A 155
645.0	7EUU	A 57	A 240	B 57	B 240
649.9	4YET	A 72	A 123	A 125	A 198
654.4	3WGT	A 52	A 107	A 132	A 320
654.5	3GE3	A 263	A 266	A 315	A 435
658.7	6ZCW	A 263	A 285	A 418	A 493
660.0	4WFO	A 574	A 649	A 665	A 666
664.3	1IK3	A 593	A 668	A 684	A 685
665.5	3TH1	A 184	A 212	B 184	B 212
666.7	2IUK	A 600	A 675	A 691	A 692
667.1	5YBL	B 138	B 139	C 138	C 139
667.2	4WWJ	A 62	A 108	A 111	A 190
667.8	3RNF	A 263	A 266	A 315	A 435

668.3	1FLG	B 248	B 270	B 414	B 489
671.2	5VN6	B 127	B 241	B 243	B 251
674.5	2IUJ	A 588	A 663	A 679	A 680
683.0	8I6Y	B 595	B 670	B 686	B 687
685.3	3GYX	G 293	G 379	G 393	L 157
686.7	1KB0	A 245	A 267	A 395	A 479
689.8	1KV9	A 232	A 254	A 383	A 460
693.8	3AWU	A 86	A 88	A 99	A 196
708.4	2VZ8	A 893	A 896	A 1024	A 1028
708.9	4W82	A 1634	A 1640	A 1807	A 1811
709.2	1MJT	A 314	A 316	B 314	B 316
725.4	8YP2	A 55	A 89	A 100	A 121
728.0	5JY1	A 117	A 120	A 121	C 117
731.6	3VCA	A 192	A 196	A 305	A 395
748.8	2FLQ	A 332	A 334	B 332	B 334
749.7	4L6V	A 2591	A 2593	A 2599	A 2616
753.8	1OOE	A 53	A 106	B 53	B 106
754.3	5IPY	A 217	A 237	A 241	A 433
762.7	1MZR	B 24	B 79	B 109	B 187
764.9	4CFS	B 36	B 127	B 160	B 185
766.3	5OY0	B 591	B 593	B 599	B 616
771.2	4IE5	A 378	A 395	A 396	A 452
773.2	8B74	A 36	A 55	A 111	A 235
786.0	4KT0	B 591	B 593	B 599	B 616
789.0	1RHC	A 43	A 119	A 229	A 246
794.2	5JHX	A 71	A 72	B 168	B 232
798.2	3VLI	A 26	A 27	B 123	B 187
799.5	1JB0	B 600	B 602	B 608	B 625
802.2	1YCG	C 149	D 263	D 347	D 376
806.4	4M7U	A 6	A 22	A 133	A 136
807.6	7DKZ	B 594	B 596	B 602	B 619
811.7	8UIV	A 75	A 205	A 206	A 356
815.0	2CIW	A 209	A 210	A 213	A 225
820.3	1X31	A 702	A 706	A 711	D 39
821.4	8ZEV	A 896	A 961	A 1022	A 1026
827.1	3BUV	B 89	B 140	B 230	B 314
834.3	2GAG	A 703	A 707	A 712	D 39
835.0	2Z5L	A 6	A 375	A 409	A 461
839.1	1ZR6	A 233	A 255	A 354	A 381
843.6	1YIQ	A 237	A 259	A 386	A 391
845.2	6JNJ	B 145	B 152	B 223	B 231
851.3	4IWK	F 28	F 34	F 80	F 87
860.9	4F40	A 25	A 81	A 113	A 197
862.7	3IWK	I 494	I 496	J 281	J 317
874.0	4HHR	A 157	A 165	A 219	A 322
875.3	1MTY	C 62	C 72	C 83	H 55
875.3	3O5U	A 47	A 50	B 47	B 50
878.2	3RKO	B 534	B 535	B 539	B 543
883.7	1C0P	A 1055	A 1078	A 1107	A 1342
884.5	6JU8	B 266	B 273	B 321	B 334
887.4	4EW6	A 144	A 151	A 222	A 230
890.3	4KVL	A 151	A 159	A 213	A 315
891.2	6FUL	A 1021	A 1193	A 1194	A 1227

893.9	6F6D	A 1266	A 1437	A 1438	A 1471
896.5	6FPO	T205	M69	M490	M495
906.6	2XXZ	B 1263	B 1434	B 1435	B 1468
911.7	3WXO	A 77	A 78	A 136	A 148
918.8	1MHY	B 65	B 75	B 86	B 98
920.0	1V9L	E 180	E 364	F 358	F 420
921.9	6VK6	B 65	B 75	B 86	B 98
922.8	3W4Y	C 95	C 157	C 179	C 183
937.6	4N4N	A 216	A 221	A 372	A 380
941.5	8VIY	A 232	A 351	A 353	A 566
943.6	2PSD	A 121	A 153	A 156	A 219
949.4	6M0Q	C 216	C 221	C 372	C 380
952.7	3HI7	A 543	B 200	B 237	B 644
962.7	4GIE	A 24	A 80	A 112	A 188
964.8	6CXH	B 48	B 51	B 54	B 60
976.9	6QPH	B 595	B 597	B 603	B 620
988.6	2AD6	A 237	A 239	A 467	A 531
989.7	5XM3	C 274	C 276	C 507	C 571
990.3	3TJT	A 159	A 161	A 195	A 214
990.6	3RGB	B 48	B 51	B 54	B 60
992.6	4BJZ	A 358	A 362	A 389	A 391
995.4	2D0V	I 243	I 245	I 476	I 540
1002.0	1LRW	C 243	C 245	C 476	C 540
1002.4	4D7E	B 91	B 104	B 117	D 104
1004.1	6FKW	A 238	A 240	A 467	A 532
1011.8	4X8E	B 233	B 234	B 239	B 288
1017.2	7CE5	C 271	C 273	C 497	C 561
1020.3	5OCS	A 37	A 114	B 335	B 352
1029.4	6SSS	D 72	E 68	F 68	F 72
1034.8	3RFT	B 204	B 213	B 214	B 224
1035.3	6UJ7	A 204	A 217	A 244	B 115
1038.9	6LKC	A 13	A 15	B 13	B 15
1039.7	3L4D	A 216	B 216	C 216	D 216
1046.2	2DE6	B 171	B 285	B 350	B 365
1047.5	6DAM	A 40	A 43	A 269	A 553
1048.2	1VBJ	A 23	A 79	A 111	A 187
1049.6	4C3O	E 69	E 232	E 493	F 205
1052.3	6OC5	A 258	A 260	A 494	A 558
1067.1	6UJ8	D 204	D 217	D 244	E 115
1069.8	2VPZ	E 151	E 519	E 520	E 537
1071.1	6RA2	F 29	F 98	F 153	F 178
1072.7	2B1X	C 203	C 326	C 376	C 431
1072.7	7O6Z	B 272	B 274	B 512	B 576
1074.1	6FOS	B 592	B 594	B 600	B 617
1075.3	1W6S	C 2243	C 2245	C 2476	C 2540
1078.4	6J55	J 128	J 130	J 163	J 182
1087.7	2Y9W	B 68	B 93	B 101	B 293
1092.2	2YIU	D 43	D 44	D 45	E 277
1098.5	6KJG	C 615	C 616	C 843	C 844
1099.0	3IBT	A 30	A 55	A 99	A 121
1100.3	4ZKY	B 89	B 90	B 137	B 140
1100.8	2AWP	A 123	A 125	A 160	A 179
1106.1	3LLK	C 408	C 476	C 478	C 503

1112.6	6ZKX	A 204	A 217	A 244	B 97
1112.8	9JP5	G 190	G 317	G 367	G 428
1113.8	2E1M	A 195	B 446	B 447	B 454
1121.2	2C91	D 77	D 224	E 77	E 224
1131.3	1ULI	C 205	C 332	C 343	C 382
1134.9	8JPW	A 203	A 454	A 455	A 462
1135.8	5U97	D 278	D 280	D 339	D 340
1136.1	6JU5	A 294	A 382	A 390	A 428
1143.6	3Q18	A 179	A 181	A 203	A 209
1146.4	1UNF	X 93	X 144	X 146	X 185
1147.2	2H30	A 64	A 67	A 112	A 143
1151.8	5AEW	G 214	G 342	G 353	G 392
1157.3	7E0C	A 195	A 446	A 447	A 454
1159.6	3F7J	A 113	A 189	B 81	B 113
1162.1	4OO2	A 349	A 384	C 349	C 384
1163.9	3QP9	B 7	B 420	B 423	B 472
1176.6	3GZX	A 214	A 340	A 351	A 390
1185.3	3FKF	A 38	A 41	A 79	A 90
1185.8	8CM6	A 491	A 492	A 728	A 765
1186.4	6KSE	B 149	B 158	B 188	B 281
1189.7	3EN1	A 203	A 330	A 341	A 380
1190.2	1KQF	A 635	A 637	A 743	A 769
1198.5	3DFR	A 5	A 21	A 133	A 158
1204.9	8ZMU	D 30	D 63	D 92	D 416
1205.6	3KRB	A 12	A 73	A 105	A 307
1212.5	7B9P	A 117	A 120	A 163	A 515
1227.9	4L4X	A 63	A 452	A 455	A 504
1235.5	6KOB	E 166	E 271	E 276	E 278
1260.6	1JMX	A 488	G 15	G 57	G 68
1264.5	6KLI	A 60	A 102	A 103	A 510
1277.9	3WBW	A 30	A 81	A 113	A 191
1290.1	6JW6	B 274	B 291	B 302	B 304
1325.2	5TX7	A 129	A 135	A 138	B 288
1348.1	5OBP	A 56	A 96	A 376	B 125
1351.3	3VDX	A 187	B 187	B 261	C 187
1352.6	4ZCD	A 81	A 103	A 212	A 229
1355.3	4ZZ7	I 133	I 481	J 133	J 481
1362.0	5T5M	A 188	A 190	A 335	B 282
1377.7	3D3L	A 222	A 340	A 550	A 553
1382.8	5T5I	A 188	A 190	A 335	B 282
1386.4	2W3W	A 10	A 26	A 100	A 143
1389.8	1D7B	B 56	B 74	B 117	B 143
1392.7	1FFT	F 170	F 190	F 280	F 282
1401.9	3O0R	B 202	B 203	B 209	C 56
1406.4	3TJR	A 120	B 111	B 113	B 120
1407.2	5B66	A 142	A 284	C 36	C 443
1415.7	2IPI	C 189	C 190	C 207	C 486
1422.6	5C2V	A 455	A 458	A 607	A 608
1423.3	4AOY	A 124	A 206	A 265	C 124
1427.4	4PJ0	A 142	A 284	C 36	C 443
1429.1	6DQW	C 22	C 23	C 75	C 294
1437.2	4XYD	A 189	A 190	A 196	B 57
1439.7	6KRI	B 149	B 158	B 188	B 281

1440.5	2AMJ	D 81	D 82	D 87	D 136
1445.7	7K62	B 5	B 21	B 92	B 135
1448.0	2RDZ	D 79	D 250	D 379	D 381
1448.0	3HLX	D 46	D 97	D 161	D 167
1451.1	1GPE	A 126	A 135	A 137	A 406
1463.5	3SDP	A 72	A 78	A 123	A 125
1468.8	6IE3	A 112	A 114	A 144	A 327
1473.6	6P73	B 76	B 243	B 372	B 374
1476.3	3WFD	B 202	B 203	B 204	B 209
1478.4	1US0	A 20	A 79	A 111	A 219
1480.3	1ZUA	X 21	X 80	X 112	X 220
1481.0	2YEV	D 405	D 406	D 499	D 506
1485.5	5SSX	A 231	A 246	A 299	A 301
1495.0	2VVM	A 97	A 103	A 463	B 463
1495.0	3AYX	A 69	A 504	B 214	B 267
1501.1	5SD5	A 6	A 22	A 92	A 135
1512.0	5APA	A 575	A 576	A 711	A 728
1513.2	7K6C	D 9	D 25	D 97	D 137
1519.5	4JO0	A 17	A 20	A 78	A 157
1530.5	2NAP	A 521	A 524	A 536	A 551
1532.8	1LOX	A 223	A 341	A 551	A 554
1538.4	1ZOR	A 124	A 204	A 262	B 124
1538.6	1WQL	A 59	A 177	A 270	A 454
1538.6	6S07	A 160	A 175	A 228	A 230
1538.7	2CW3	A 110	A 137	A 191	A 196
1544.8	3QVP	A 122	A 131	A 133	A 402
1557.3	5ZSX	A 45	A 48	A 136	A 147
1562.2	2BOY	D 37	D 47	D 184	H 47
1562.3	6UWW	A 16	A 32	A 106	A 149
1568.8	4LGV	C 307	C 309	D 196	D 309
1572.3	5A7E	A 64	A 74	A 106	A 445
1578.3	3S8F	A 157	A 190	A 193	A 230
1579.7	3H7U	A 24	A 83	A 115	A 216
1582.1	7ODH	L 68	L 511	S205	S258
1589.6	5Z1X	A 65	A 75	A 107	A 445
1589.9	3PXL	A 65	A 75	A 107	A 449
1592.8	3W36	B 99	B 146	B 350	B 353
1595.5	6YYX	A 575	A 576	A 711	A 728
1600.2	5EHF	A 65	A 75	A 107	A 447
1601.3	3KW7	B 65	B 75	B 107	B 452
1601.9	2A9D	A 392	A 413	A 457	B 447
1603.6	7L9T	A 7	A 23	A 90	A 133
1608.2	6RI6	A 66	A 76	A 108	A 449
1610.4	2QT6	B 65	B 75	B 107	B 448
1610.5	3HHD	C 493	C 712	C 722	C 750
1611.3	3X1B	A 86	A 96	A 128	A 471
1611.8	1GYC	A 65	A 75	A 107	A 449
1616.0	1Q16	A 1095	A 1118	B 40	B 144
1616.3	1KYA	D 65	D 75	D 107	D 449
1620.4	1HFU	A 65	A 75	A 107	A 448
1625.4	2HZH	A 65	A 75	A 107	A 449
1633.3	1SOX	A 392	A 413	A 457	B 447
1638.2	2BII	A 350	A 389	A 432	A 474

1641.1	2XYB	A 65	A 75	A 107	A 447
1652.9	7U6L	C 69	C 176	C 196	C 390
1659.6	3T6V	A 66	A 76	A 108	A 449
1670.9	2EBA	F 293	F 327	I 293	I 327
1684.4	2Z5Y	A 144	A 193	A 196	A 472
1691.1	2J7A	G 148	G 158	G 167	G 513
1691.8	2NYA	F 115	F 118	F 441	F 509
1692.5	2VR0	B 148	B 158	B 167	B 513
1705.5	3EAU	A 57	A 121	A 243	A 272
1705.8	1OAH	B 147	B 157	B 166	B 513
1710.2	2JFK	D 493	D 712	D 722	D 750
1729.8	4HA7	A 228	A 229	B 228	B 229
1732.8	3KIJ	A 164	A 167	B 164	C 164
1736.6	1OGY	M124	M127	M451	M519
1743.5	2NW8	A 61	A 236	B 61	B 102
1756.2	8FW1	A 66	A 100	A 232	A 342
1780.4	4UDQ	A 117	A 125	A 127	A 305
1814.3	8J83	A 775	A 776	A 789	A 813
1816.0	3GR7	A 290	A 316	B 290	B 316
1827.7	2NOX	I 78	I 253	J 78	J 119
1856.3	3ML1	A 124	A 127	A 451	A 519
1922.4	6JDK	A 56	A 58	A 86	A 93
1954.5	3PUA	A 217	A 222	A 371	A 409
1957.8	6IQX	B 200	B 361	B 396	B 433
1988.3	4U3E	B 268	B 270	B 312	B 317
1999.8	1Z01	B 185	B 265	B 271	B 307
2019.1	5M8S	D 90	D 190	D 223	D 444
2036.8	3JS8	A 225	A 266	A 270	A 336
2103.7	3K3O	A 215	A 220	A 369	A 407
2130.4	4USQ	A 108	A 128	A 140	F 108
2146.1	1WVF	A 285	A 321	A 386	A 394
2146.7	3SQR	A 177	A 367	A 378	A 493
2150.5	3KV9	A 250	A 255	A 404	A 442
2158.3	6HF1	F 36	F 47	F 50	F 106
2165.6	1WVE	A 285	A 321	A 386	A 394
2176.4	2Q9O	B 94	B 127	B 136	B 499
2222.1	3US8	A 124	A 259	A 265	B 124
2452.1	1AFR	B 46	B 132	B 135	B 308
2471.8	1KF6	O 86	O 113	P 14	P 23
2489.2	2UW1	A 41	A 127	A 130	A 303
2496.5	3CIR	O 86	O 113	P 14	P 23
2527.5	5NLT	C 86	C 102	C 129	C 222
2578.5	2XSJ	D 185	D 240	E 41	E 43
2618.8	7PP7	A 41	A 127	A 130	A 303
2624.8	3K6H	A 115	B 53	B 115	B 141
2632.2	8RPG	A 95	A 116	A 129	A 195
2647.3	4OUA	A 64	A 90	A 280	A 337
2740.1	6PYZ	C 82	C 88	C 324	D 82
2893.4	8BBQ	B 94	B 101	B 291	B 315

Table S2.2. Characterization of tryptophan quadruplexes in EC-1 oxidoreductases.
First column: PDB code
Second column: Number tryptophan residues at protein-protein interfaces.
(For example, 1:1:1:1 denote a tetramer of monomers bearing one tryptophan each.
2:2:0:0 denotes a dimer linked by two tryptophans from each unit.
4:0:0:0 denotes purely intramolecular quadruplexes.)
Centers: indole center-center distances in Å. Tryptophans are numbered 1-4 in the order they are listed in Table S2.1.
Closest: shortest distances between indole heavy atoms in Å.
Tryptophans are numbered 1-4 in the order they are listed in Table S2.1.

PDB		Centers:	1-2	1-3	1-4	2-3	2-4	3-4	Closest:	1-2	1-3	1-4	2-3	2-4	3-4
3l4d	1:1:1:1	:	10.91	8.03	6.56	6.9	13.97	10.03	:	7.83	7.79	3.82	3.79	10.02	6.63
6sss	1:1:2:0	:	10.15	14.78	11.95	10.16	12.5	6.72	:	7.98	12.43	8.77	8.6	10.3	3.4
4dgq	1:2:1:0	:	13.17	9.4	9.43	9.43	18.22	9.51	:	9.68	5.79	5.87	5.88	14.96	5.96
1a88	1:2:1:0	:	13.23	9.58	9.58	9.41	18.25	9.56	:	9.76	6.04	6.06	5.91	15.07	6.06
3vdx	1:2:1:0	:	12.31	12.35	11.92	16.51	12.25	23.1	:	8.95	9.68	8.56	14.25	8.81	20.47
8u3p	1:3:0:0	:	8.58	9.88	4.04	5.23	7.05	10.44	:	6.19	6.16	3.39	3.49	3.39	6.88
1yq9	1:3:0:0	:	10.23	7.85	6.23	5.1	5.04	6.09	:	8.05	6.73	3.4	3.61	3.63	3.65
4xyb	1:3:0:0	:	7	6.4	12.27	13.2	18.42	6.42	:	3.57	3.43	8.6	10.43	15.56	4.44
1ycg	1:3:0:0	:	8.17	6.25	6.43	13.13	10.38	7.28	:	5.15	3.41	3.43	9.24	6.65	4.09
6fpo	1:3:0:0	:	9.82	5.25	13.25	10.65	14.34	8.21	:	7.63	3.32	9.98	6.71	10.72	6.45
3hi7	1:3:0:0	:	4.98	11.1	11.39	8.22	14.29	21.19	:	3.26	7.48	7.92	5.52	10.3	18.55
2e1m	1:3:0:0	:	10.93	12.67	11.47	5.17	6.81	10.42	:	6.87	9.03	8.75	3.58	4.52	8.18
1jmx	1:3:0:0	:	7.07	5.56	9.77	10.1	13.64	12.55	:	3.54	3.71	7.01	6.77	11.05	9.99
3tjr	1:3:0:0	:	7.28	12.87	8.32	12.59	14	13.17	:	4.51	9.6	4.76	9.57	10.65	10.33
3k6h	1:3:0:0	:	13.29	10.82	9.58	15.4	13.2	15.71	:	9.27	7.67	7.25	13.01	10.71	12.62
4phz	2:1:1:0	:	6.76	7.49	5.83	12.78	5.36	13.31	:	4.47	3.6	2.86	8.97	3.38	10.32
3kij	2:1:1:0	:	9.59	9.95	9.74	18.3	17.88	9.82	:	5.78	7.33	7.08	15.66	14.62	7.04
6mjs	2:2:0:0	:	7.23	7.64	4.9	5.09	6.84	7.19	:	4.05	3.69	3.27	3.35	4.8	4.05
6mjs	2:2:0:0	:	6.5	7.84	5.28	5.25	6.03	6.88	:	3.64	3.66	3.25	3.13	3.47	3.76
8dq8	2:2:0:0	:	6.02	9.47	6.8	15.45	12.51	3.66	:	3.99	6.42	3.87	13.04	10.32	3.25
8jfj	2:2:0:0	:	5.6	7.22	9.75	9.81	8.95	5.57	:	3.5	3.36	6.48	6.54	4.64	3.54
6aqz	2:2:0:0	:	13.27	7.73	6.37	6.3	18.11	13.37	:	9.02	4.03	3.67	3.57	13.86	9.04
4i8p	2:2:0:0	:	6.92	7.14	4.67	5.74	10.42	11.21	:	4.43	3.93	3.15	3.55	7.83	7.27
2weu	2:2:0:0	:	5.74	6.52	11.09	11.1	16.38	5.71	:	3.57	3.93	8.15	8.13	15.03	3.57
5z76	2:2:0:0	:	10.06	6.41	4.82	4.81	13.8	10.05	:	6.68	3.46	3.5	3.52	10.09	6.67
3iwj	2:2:0:0	:	6.85	7.24	11.74	6.69	12.19	17.53	:	4.44	3.56	7.98	3.77	8.27	13.36
3o0k	2:2:0:0	:	7.45	13.53	7.86	7.96	4.16	7.46	:	3.61	9.24	3.8	3.91	3.57	3.63
6ixj	2:2:0:0	:	4.78	6.82	9.57	9.68	11.38	4.6	:	3.42	3.47	7.1	6.84	7.48	3.31
2qjy	2:2:0:0	:	8.31	3.84	12.03	12.09	20	8.27	:	5.84	3.43	9.56	9.48	16.42	5.87
2yy7	2:2:0:0	:	10.32	6.82	5.02	4.97	14.1	10.43	:	6.99	3.76	3.46	3.5	10.3	7.07
5a2d	2:2:0:0	:	10.48	5.31	11.16	7.33	4.95	6.85	:	6.76	3.2	8.51	4.08	4	4.02
2oq2	2:2:0:0	:	5.88	9.43	9.65	9.56	6.78	5.97	:	3.67	6.65	7.79	7.76	3.44	3.7
7cl2	2:2:0:0	:	9.49	8.22	5.84	5.8	7.01	9.43	:	6.17	4.05	3.8	3.83	3.48	6.18
4j6v	2:2:0:0	:	5.12	9.81	9.79	9.75	11.66	5.12	:	3.66	6.58	6.98	6.93	7.31	3.62
2q17	2:2:0:0	:	9.76	7.81	12.31	15.54	21.5	6.79	:	8.05	3.97	8.79	13.3	19.05	3.56
5ybn	2:2:0:0	:	5.73	6.13	5.08	5.15	8.66	5.79	:	3.14	3.22	3.39	3.46	5.68	3.09
5xbr	2:2:0:0	:	5.94	12.32	9.55	7.27	5.14	7.94	:	3.55	9.92	7.37	4.01	3.63	5.03
7euu	2:2:0:0	:	8.44	7.75	7.2	7.13	3.55	8.84	:	5.04	3.49	4.2	4.12	3.36	5.38
3th1	2:2:0:0	:	4.86	12.21	10.42	10.31	7.29	4.86	:	3.39	7.75	6.35	6.51	3.37	3.48
5ybl	2:2:0:0	:	6.02	6.08	5.12	5.48	9.05	5.94	:	3.38	3.05	3.4	3.51	6.06	3.04
1mjt	2:2:0:0	:	11.54	7.97	9.83	9.7	19.8	11.71	:	8.27	3.91	7.27	7.33	15.35	8.38
2flq	2:2:0:0	:	12.05	8.41	9.5	9.79	19.71	11.64	:	8.93	4.4	7.14	7.28	15.44	8.38
1ooe	2:2:0:0	:	4.48	12.67	10.58	11.08	9.51	4.36	:	3.41	9.76	7.9	8.33	6.74	3.35
5jhx	2:2:0:0	:	6.08	8.06	9.91	9.58	12.31	5.41	:	3.68	5.27	6.46	5.21	8.89	3.64
3vli	2:2:0:0	:	6.29	8.66	9.94	10.09	12.62	5.31	:	4.13	6.41	6.47	5.76	8.99	3.53
3iwk	2:2:0:0	:	7.29	4.72	11.68	10.94	11.95	10.77	:	4.62	3.16	7.98	8.31	8.04	9.13
3o5u	2:2:0:0	:	6.35	6.31	11.37	11.35	15.31	6.54	:	3.96	4.36	9.99	9.99	12.88	4.12
1v9l	2:2:0:0	:	6.11	11.81	10.66	6.24	14.77	18.73	:	3.38	8.59	7.71	3.38	11.2	15.23
5ocs	2:2:0:0	:	9.44	11.85	11.59	14.9	7.17	10.14	:	7.43	8.05	9.43	12.02	4.69	7.11
6lkc	2:2:0:0	:	6.75	8.25	8.22	8.18	7.26	6.7	:	3.47	3.83	4.66	4.63	5.14	3.42
2c91	2:2:0:0	:	6.33	10.55	12.06	12.05	14.36	6.23	:	4.06	7.09	9.5	9.56	11.16	4.03
3f7j	2:2:0:0	:	8.05	10.59	13.77	13.4	19.42	8.06	:	6.3	7.96	12.54	10.39	16.72	4.17
4oo2	2:2:0:0	:	10.7	9.36	7.29	7.31	15.31	10.66	:	7.62	5.63	4.51	4.57	12.64	7.63
4zz7	2:2:0:0	:	10.52	6.73	11.65	11.72	7.94	10.5	:	7.15	6.4	8.37	8.53	5.6	7.22
5b66	2:2:0:0	:	12.27	11.43	3.76	16.39	10.05	9.96	:	8.99	7.96	3.34	13.02	6.46	6.42
4pj0	2:2:0:0	:	12.41	11.33	3.76	16.32	10.18	9.92	:	8.99	7.99	3.29	12.95	6.64	6.4
3ayx	2:2:0:0	:	10.69	9.95	10.23	5.34	18.09	18.2	:	6.73	7.64	7.73	3.46	14.15	14.89
4lgv	2:2:0:0	:	12.64	10.42	12.33	21.91	20.63	7.24	:	9.43	7.1	9.09	19.21	18.76	3.67
7odh	2:2:0:0	:	10.76	10.01	10.26	5.42	17.98	17.99	:	6.77	7.91	7.7	3.36	14.04	14.76
1q16	2:2:0:0	:	9.59	11.08	11.09	18.9	10.77	13.29	:	6.48	8.46	9.3	16.87	9.7	10.92
2eba	2:2:0:0	:	8.27	10.57	11.57	11.45	15.03	8.33	:	6.66	7.32	8.1	7.97	11.68	6.93
4ha7	2:2:0:0	:	10.56	8.78	10.4	10.2	14.79	10.68	:	7.93	6.34	8.17	8.12	13.31	8.15
2nw8	2:2:0:0	:	12.21	8.32	11.35	14.08	22.42	12.74	:	8.57	4.99	9.08	10.62	19.27	9.93
3gr7	2:2:0:0	:	12.63	12.63	11.17	11.42	19.71	12.64	:	9.6	8.35	8.87	9.15	17.23	9.6
2nox	2:2:0:0	:	12.54	8.89	11.22	13.6	22.48	13.45	:	8.96	5.28	9.05	10.32	19.51	10.5
1kf6	2:2:0:0	:	12.09	9.66	12.28	21.13	16.26	18.25	:	9.76	7.28	9.79	19.44	14.39	16.4
3cir	2:2:0:0	:	12.05	9.75	12.14	21.15	16.32	18.04	:	9.52	6.97	9.64	19.3	14.29	16.09
2xsj	2:2:0:0	:	9.76	11.88	11.32	18.26	18.4	10.25	:	6.61	9.63	8.74	14.97	15.49	8.17
5sxw	3:1:0:0	:	5.32	7.1	8.68	10.54	10.15	3.82	:	3.62	3.49	6	7.16	6.42	3.31
5whs	3:1:0:0	:	5.37	7.05	8.72	10.46	10.1	3.89	:	3.55	3.79	6.33	7.35	6.63	3.28
2ivf	3:1:0:0	:	5.66	7.54	7.34	7.16	12.17	9.09	:	3.42	4	5.05	3.74	8.74	7.02
3gyx	3:1:0:0	:	6.34	5.87	12.16	6.25	18.05	13.85	:	3.49	3.44	9.05	3.85	16.88	12.04
5jy1	3:1:0:0	:	6.37	5.36	10.27	7.45	15.35	15.05	:	3.88	3.62	6.81	3.67	13.26	12.32

1x31	3:1:0:0	:	4.99	10.13	13.08	5.79	11.13	13.58	:	3.14	7.94	9.45	3.5	6.87	9.83
2gag	3:1:0:0	:	5.07	9.88	13.42	5.5	11.48	13.6	:	3.29	7.68	9.77	3.34	7.24	9.84
1mty	3:1:0:0	:	6.05	10.72	9.89	6.26	15.39	18.98	:	3.52	8.08	5.94	3.65	12.53	15.57
4d7e	3:1:0:0	:	5.21	6.73	13.79	11.21	11.24	17.44	:	3.59	3.9	9.51	8.58	7.42	13.66
6uj7	3:1:0:0	:	10.99	5.92	11.3	10.58	21.72	13.05	:	7.79	3.67	7.74	7.36	19.01	9.64
4c3o	3:1:0:0	:	6.96	11.42	10.23	16.04	13.49	5.44	:	3.99	7.96	8.09	12.41	11.47	3.27
6uj8	3:1:0:0	:	10.94	5.81	11.15	10.6	21.49	12.83	:	7.84	3.45	7.72	7.39	18.86	9.47
2yiu	3:1:0:0	:	9.26	10.93	9.35	8.76	16.82	13.09	:	6.88	8.39	7.86	5.29	14.42	10.33
6zkx	3:1:0:0	:	11.11	5.86	12.09	10.6	22.65	13.88	:	8.08	3.47	8.56	7.34	19.8	10.41
5tx7	3:1:0:0	:	8.95	11.92	11.82	9.54	6.06	7.08	:	6.47	9.54	8.92	6.75	3.42	4.13
5obp	3:1:0:0	:	10.14	6.41	11.1	10.56	6.65	13.24	:	6.99	3.64	8.6	7.04	3.73	10.43
5t5m	3:1:0:0	:	10.81	6.75	12.74	16.51	12.86	12.86	:	8.62	3.65	9.87	13.43	10.38	8.98
5t5i	3:1:0:0	:	10.92	6.8	12.82	16.67	12.98	12.95	:	8.75	3.77	9.87	13.65	10.49	9.07
3o0r	3:1:0:0	:	5.04	10.38	12.49	12.04	15.44	21.91	:	3.27	9.27	9.93	9.71	13.04	18.9
4aoy	3:1:0:0	:	10.98	9.58	12.13	6.85	20.9	17.11	:	9.09	6.34	9.91	5.19	17.58	13.02
4xyd	3:1:0:0	:	5.35	10.38	12.29	12.07	15.44	21.83	:	3.57	9.43	9.82	9.78	12.99	18.96
2vvm	3:1:0:0	:	5.67	11.9	8.85	11.98	10.05	14.02	:	3.84	9.91	5.6	9.77	6.71	11.4
1zor	3:1:0:0	:	11.2	10.27	11.18	7.24	19.34	16.46	:	8.69	7.26	9.39	5.37	15.46	12.55
2boy	3:1:0:0	:	8.27	11.58	9.57	17.1	6.07	19.47	:	5.55	8.92	7.78	13.22	3.31	16.2
2a9d	3:1:0:0	:	11.9	6.08	12.07	13.17	22.26	15.37	:	9.15	3.88	8.77	10.91	20.48	12.1
1sox	3:1:0:0	:	11.85	6.09	12.42	13.29	22.55	15.51	:	9.19	3.96	9.14	11	20.84	12.22
4usq	3:1:0:0	:	13.17	11.66	7.66	22.54	15.13	18.23	:	9.87	7.89	4.01	19.49	11.99	14.53
3us8	3:1:0:0	:	10.44	9.89	11.72	9.69	18.12	16.9	:	7.29	6.65	9.71	7.92	15.56	12.76
6pyz	3:1:0:0	:	13.03	12.78	9.44	20.91	21.51	14.39	:	9.87	9.35	5.7	18.29	18.57	11.17
6fcx	4:0:0:0	:	9	10.03	4.98	5.17	8.44	6.73	:	7.18	8.34	3.79	3.3	4.89	3.85
8aep	4:0:0:0	:	7.3	6.09	5.69	6.93	12.01	7.23	:	5.54	3.68	3.75	4.4	9.97	3.7
2xts	4:0:0:0	:	11.12	5.9	7.47	10.85	5.19	5.68	:	7.27	3.74	3.85	8.29	3.38	3.59
5l6g	4:0:0:0	:	6.84	6.56	11.74	7.95	9.66	5.82	:	4.22	3.73	8.52	4.11	6.46	3.69
1h0h	4:0:0:0	:	8.42	7.4	7.3	5.89	14.66	14.5	:	5.89	3.67	3.86	3.59	11.47	10.34
3l9w	4:0:0:0	:	6.46	8.15	8.46	13.95	14.81	6.92	:	3.36	5.1	4.88	10.72	10.66	4.11
7u9u	4:0:0:0	:	6.92	13.27	13.49	6.71	20.11	26.7	:	4.33	9.64	9.68	3.68	16.86	22.92
6qgr	4:0:0:0	:	6.81	5.59	12.86	10.21	9.04	12.16	:	4.19	4.02	8.55	8.2	5.16	8.02
8qdq	4:0:0:0	:	11.6	7.09	7.77	10.62	6.31	5.19	:	7.58	3.93	4.39	7.33	3.79	3.31
2bs2	4:0:0:0	:	5.28	8.16	10.13	12.29	14.66	6.13	:	3.57	5.55	7.66	10.07	12.44	3.81
3pf7	4:0:0:0	:	12.43	10.14	5.76	5.53	10.29	5.84	:	9.64	7.61	3.63	3.78	7.56	3.55
4yet	4:0:0:0	:	10	8.04	7.45	5.99	13.85	14.55	:	8.11	4.06	4.06	4.24	10.81	10.15
3wgt	4:0:0:0	:	7.27	13.49	12.88	6.65	19.82	26.31	:	4.7	9.99	9.19	3.82	16.79	22.77
3ge3	4:0:0:0	:	6.26	8.25	9.38	13.68	15.33	4.66	:	3.83	7.21	6.8	11.08	11.51	3.49
6zcw	4:0:0:0	:	11.81	9.03	10.49	18.01	14.77	6.23	:	9.52	5.11	7.14	14.27	11.02	3.27
4wfo	4:0:0:0	:	11.66	7.2	7.45	10.87	6.56	5.12	:	7.65	4.01	3.75	7.54	3.9	3.27
1ik3	4:0:0:0	:	12.03	7.17	7.77	10.76	6.59	5.07	:	8.19	4.03	4.3	7.5	3.99	3.36
2iuk	4:0:0:0	:	11.57	6.96	7.25	10.72	6.69	4.92	:	7.56	3.84	3.57	7.44	4.07	3.3
4wwj	4:0:0:0	:	6.86	11.91	10.72	5.71	5.22	6.3	:	3.59	9.09	7.86	3.48	3.52	3.67
3rnf	4:0:0:0	:	6.43	8.02	9.42	13.54	15.52	4.67	:	3.86	6.92	6.75	10.81	11.66	3.51
1flg	4:0:0:0	:	11.72	8.46	11.06	17.32	15.09	6.31	:	9.44	4.59	7.71	13.56	11.5	3.41
5vn6	4:0:0:0	:	8.28	7.79	5.09	5.71	11.71	9.75	:	4.72	4.23	3.19	3.65	8.86	6.1
2iuj	4:0:0:0	:	12.03	7.13	7.7	10.71	6.69	4.89	:	8.38	3.89	3.92	7.79	4.24	3.41
8i6y	4:0:0:0	:	11.75	7.25	7.81	10.67	6.33	5.24	:	8.14	3.97	4.52	7.42	3.78	3.35
1kb0	4:0:0:0	:	11.62	8.69	11.62	17.19	15.17	6.46	:	9.39	4.67	7.5	13.45	11.59	3.9
1kv9	4:0:0:0	:	11.68	8.54	11.62	16.89	14.88	6.46	:	9.44	4.61	7.58	13.29	11.46	3.88
3awu	4:0:0:0	:	6.23	5.71	7.25	11.65	9.67	7.1	:	3.76	3.71	3.95	8.03	7.59	4.03
2vz8	4:0:0:0	:	7.86	11.58	8.24	11.81	5.91	6	:	4.44	8.46	4.56	7.6	3.76	3.94
4w82	4:0:0:0	:	10.81	6.82	4.85	17.33	13.97	7.23	:	7.99	3.66	3.24	13.6	11.67	4.26
8yp2	4:0:0:0	:	7.3	8.11	13.74	14.34	6.78	20.26	:	4.19	4.39	9.84	10.44	3.39	16.05
3vca	4:0:0:0	:	7.52	12.52	9.08	6.13	8.57	14.39	:	3.64	8.86	4.66	3.94	6.74	11.71
4l6v	4:0:0:0	:	12.66	7.08	13.3	12.91	5.1	15.95	:	9.71	3.73	9.84	9.66	3.45	13.3
5ipy	4:0:0:0	:	9.87	8.3	8.09	3.87	12.85	12.18	:	7.77	5.37	4.15	3.38	9.17	7.99
1mzr	4:0:0:0	:	11.39	9.69	6.19	7.68	12.74	7.03	:	8.72	6.64	3.52	3.78	9.89	4.22
4cfs	4:0:0:0	:	11.69	12.92	9.35	13.08	16.82	8.63	:	8.41	9.13	7.2	9.8	13	4.42
5oy0	4:0:0:0	:	12.56	6.86	13.25	12.9	5.1	15.9	:	9.58	3.58	9.71	9.68	3.37	13.07
4ie5	4:0:0:0	:	12.01	8.01	12.24	5.22	21.37	16.23	:	7.68	4.02	9.9	3.62	18.08	14.33
8b74	4:0:0:0	:	10.3	12.13	6.48	21.96	12.6	10.89	:	7.09	8.37	3.58	18.36	9.39	7.67
4kt0	4:0:0:0	:	12.63	7.02	13.51	12.88	5.1	16.08	:	9.68	3.7	9.97	9.76	3.37	13.27
1rhc	4:0:0:0	:	12.29	8.29	7.39	19.59	19.34	3.93	:	9.91	5.85	3.92	15.82	15.85	3.18
1jb0	4:0:0:0	:	12.58	7.16	13.32	12.77	5.16	15.96	:	9.56	3.91	9.74	9.56	3.42	13.25
4m7u	4:0:0:0	:	11.21	7.95	9.93	17.35	21.05	7.33	:	7.2	4.44	7.71	13.83	19.26	4.69
7dkz	4:0:0:0	:	12.53	7.19	13.44	12.53	5.31	15.94	:	9.6	3.83	9.87	9.43	3.5	13.27
8uiv	4:0:0:0	:	11.36	7.64	7.1	10.29	17.76	10.02	:	9.56	5.18	4.65	6.86	14.45	6.6
2ciw	4:0:0:0	:	11.54	10.68	6.33	7.63	13.09	8.17	:	8.11	8.23	3.7	4.04	9.87	7.16
8zev	4:0:0:0	:	11.6	11.5	5.86	17.92	13.75	5.67	:	9.36	7.3	3.74	14.74	10.48	3.6
3buv	4:0:0:0	:	10.21	9.31	7	10.09	5.16	7.33	:	7.07	6.12	3.71	6.4	3.37	3.68
2z5l	4:0:0:0	:	12.59	10.23	11.53	12.78	6.91	16.71	:	9.17	6.66	8.13	11.43	3.79	14.46
1zr6	4:0:0:0	:	8.12	12.72	10.79	10.79	12.28	4.91	:	6.1	9.56	7.04	8.98	10.31	3.17
1yiq	4:0:0:0	:	12.09	11.53	8.59	10.49	17.58	10.78	:	9.91	7.76	4.67	7.89	13.77	7.26

6jnj	4:0:0:0	:	10.32	10.76	6.13	9.74	5.52	10.87	:	9	6.86	3.57	6.65	3.64	7.23
4iwk	4:0:0:0	:	7.7	6.13	10.68	12.42	9.66	10	:	4.08	3.76	7.69	10.05	7.31	8.88
4f40	4:0:0:0	:	12.43	10.03	6.22	7.65	12.73	6.88	:	8.84	7.76	4.27	3.81	9.95	4.24
4hhr	4:0:0:0	:	9.32	8.52	6.23	5.1	9.32	10.15	:	6.7	6.08	3.27	3.13	6.24	7.64
3rko	4:0:0:0	:	7.72	6.65	10.31	5.52	16.96	14.02	:	4.67	3.71	8.94	3.31	13.78	11.68
1c0p	4:0:0:0	:	7.63	7.48	13.52	14.7	9.25	18.15	:	4.52	4.34	9.86	12.15	6.65	13.83
6ju8	4:0:0:0	:	8.19	12.07	5.7	9.43	13.65	16.49	:	6.62	10.76	3.53	6.7	10.42	14.32
4ew6	4:0:0:0	:	11.42	11.17	6.24	9.68	6.58	11.23	:	9.58	7.54	3.73	5.96	4.25	7.64
4kvl	4:0:0:0	:	9.31	8.33	6.24	5.07	9.64	10.24	:	6.77	5.94	3.3	3.22	6.61	7.72
6ful	4:0:0:0	:	6.93	9.48	8.55	9.41	7.53	5.73	:	3.95	7.5	6.74	7.15	4.79	3.6
6f6d	4:0:0:0	:	6.77	9.69	8.81	9.06	7.63	5.71	:	3.81	7.58	6.9	6.87	5.05	3.54
2xxz	4:0:0:0	:	6.73	9.56	8.61	9.48	8.12	5.76	:	3.82	7.56	6.78	7.21	5.37	3.56
3wxo	4:0:0:0	:	7.69	11.42	13.87	17.38	17.09	6.75	:	4.88	7.78	9.77	13.9	13.87	3.85
1mhy	4:0:0:0	:	6.09	10.97	12.6	6.41	13.61	11.32	:	3.32	8.25	9.46	3.61	10.25	9.24
6vk6	4:0:0:0	:	6.06	11	12.75	6.45	13.7	11.35	:	3.34	8.29	9.61	3.73	10.35	9.29
3w4y	4:0:0:0	:	10.63	11.32	8.79	11.1	14.4	6.54	:	6.74	8.1	6.34	8.96	11.99	3.62
4n4n	4:0:0:0	:	5.99	6.14	13.26	12.1	15.98	12.49	:	3.45	3.56	9.75	7.89	13.23	9.57
8viy	4:0:0:0	:	8.58	11.95	5.28	11.92	12.26	17.07	:	4.82	9.63	3.17	9.42	8.69	14.08
2psd	4:0:0:0	:	13.39	11.35	5	5.94	17.65	15.97	:	10	8.49	3.32	3.81	14.32	12.87
6m0q	4:0:0:0	:	6.09	5.97	13.3	12.03	16.13	12.51	:	3.53	3.47	9.79	7.81	13.31	9.53
4gie	4:0:0:0	:	9.99	10.31	9.58	7.96	13.08	6.77	:	7.89	8.53	7.14	4.02	10.24	3.99
6cxh	4:0:0:0	:	11.09	6.13	8.25	6.29	12.86	10.55	:	7.75	3.86	4.58	3.61	10.06	8.03
6qph	4:0:0:0	:	12.43	8.1	13.47	12.56	5.25	16.27	:	9.89	5.52	9.74	10.18	3.84	14.37
2ad6	4:0:0:0	:	9.31	10.13	9.97	11.64	16.41	7.43	:	6.97	6.84	6.88	9.3	13.38	4.56
5xm3	4:0:0:0	:	9.41	10.15	9.65	11.84	16.28	7.28	:	7.28	6.89	6.54	9.47	13.33	4.49
3tjt	4:0:0:0	:	5.39	6.94	10.06	11.55	10.64	15.17	:	3.66	3.78	7.46	8.5	8.21	12.58
3rgb	4:0:0:0	:	11.09	6.33	8.06	6.46	12.53	10.47	:	7.56	3.92	4.35	3.68	9.69	7.73
4bjz	4:0:0:0	:	4.54	9.27	9.83	13.48	12.65	8.51	:	3.24	7.2	7.64	11.07	9.62	7.93
2d0v	4:0:0:0	:	9.66	10.23	9.55	11.74	16.16	7.16	:	7.42	6.84	6.43	9.34	13.1	4.23
1lrw	4:0:0:0	:	9.27	10.24	9.94	11.55	16.24	7.41	:	7.22	6.89	6.92	8.92	13.54	4.32
6fkw	4:0:0:0	:	9.25	10.51	9.62	11.64	16.14	7.86	:	7.07	7.2	6.85	9.06	13.39	5.01
4x8e	4:0:0:0	:	8.55	10.7	6.27	6.02	11.02	14.88	:	4.8	7.18	3.65	3.65	8.29	11.15
7ce5	4:0:0:0	:	9.4	10.16	9.67	11.9	16.42	7.49	:	7.18	6.94	6.63	9.42	13.43	4.69
3rft	4:0:0:0	:	9.99	4.69	12.11	5.81	17.26	12.79	:	7.57	3.43	7.96	3.78	14.26	8.98
2de6	4:0:0:0	:	12.62	9.95	7.74	21.54	13.04	11.25	:	9.03	8.02	4.14	17.61	9.79	7.49
6dam	4:0:0:0	:	8.81	11.34	8.59	4.74	16.49	17.82	:	6.71	8.55	5.99	3.41	13.89	14.44
1vbj	4:0:0:0	:	10.86	10.28	8.3	7.51	12.72	7.17	:	8.52	8.45	5.81	3.58	9.91	4.43
6oc5	4:0:0:0	:	9.59	10.66	9.06	11.88	16.02	7.97	:	7.68	7.43	6.05	9.15	13.4	5.32
2vpz	4:0:0:0	:	7.48	11.86	6.88	6.93	7.12	11.41	:	5.02	9.97	3.68	3.78	3.62	7.53
6ra2	4:0:0:0	:	10.33	10.21	9.37	14.77	17.96	7.57	:	6.35	6.68	7.45	11.42	14.44	3.65
2b1x	4:0:0:0	:	8.62	11.55	7.38	6.5	14.95	16.2	:	5.26	9.95	4.23	3.71	11.4	15.13
7o6z	4:0:0:0	:	9.54	10.86	9.8	11.82	16.38	8.02	:	7.42	7.37	6.89	9.47	13.58	5.28
6fos	4:0:0:0	:	11.95	9.06	13.51	11.09	6.23	16.15	:	9.24	6.78	9.76	9.53	3.4	13.86
1w6s	4:0:0:0	:	9.75	10.5	10	11.93	16.72	7.65	:	7.54	7.13	6.9	9.5	13.8	4.7
6j55	4:0:0:0	:	5.75	6.45	10.72	11.47	11.18	15.3	:	3.81	3.55	8.28	8.36	9.28	12.39
2y9w	4:0:0:0	:	10.78	11.65	11.59	14	11.13	3.96	:	8.66	9.31	9.56	11.06	7.35	3.59
6kjg	4:0:0:0	:	9.09	9.21	7.22	16.47	12.28	6.13	:	7.28	5.85	4	12.93	9.62	3.52
3ibt	4:0:0:0	:	7.29	9.83	12.29	12.72	13.08	5	:	6.12	5.89	8.95	9.87	11.53	3.49
4zky	4:0:0:0	:	8.95	12.89	9.63	12.05	5.87	16.59	:	6.09	9.15	6.56	8.75	3.67	13.5
2awp	4:0:0:0	:	6.05	6.44	11.25	11.88	11.35	15.82	:	4.17	3.59	8.52	8.88	9.46	12.64
3llk	4:0:0:0	:	10.69	11.66	10.51	10.99	16.26	8.07	:	6.87	8.83	9.53	8.68	13.82	4.42
9jp5	4:0:0:0	:	9.02	11.85	7.39	6.6	15.27	16.28	:	5.75	9.99	4.1	3.67	11.65	15.17
1uli	4:0:0:0	:	8.84	10.15	9.71	11.31	6.97	16.36	:	5.83	6.92	6.77	8.31	3.82	11.92
8jpw	4:0:0:0	:	10.92	12.94	11.37	5.2	6.78	10.23	:	6.82	9.38	8.69	3.53	4.26	7.81
5u97	4:0:0:0	:	10.66	10.84	6.77	11.29	10.76	4.88	:	8.38	7.28	3.96	9.94	7.91	3.31
6ju5	4:0:0:0	:	7.06	5.27	12.51	10.04	12.36	10.3	:	3.77	3.55	8.79	6.41	9.07	7.13
3q18	4:0:0:0	:	10	6.75	11.64	12.2	21.44	11.9	:	7.63	3.84	9.64	8.51	19.41	9.86
1unf	4:0:0:0	:	9.42	8.08	12.11	5.92	12.21	8	:	7.54	3.97	9.65	3.84	8.88	5.05
2h30	4:0:0:0	:	9.93	8.22	10.62	10.84	16.03	18.48	:	8.42	4.04	8.26	8.74	13.85	15.45
5aew	4:0:0:0	:	8.96	10.31	9.97	11.63	6.97	16.81	:	5.9	7.26	6.84	8.6	4	12.42
7e0c	4:0:0:0	:	10.98	12.91	11.57	5.28	6.73	10.23	:	6.89	9.25	8.96	3.64	4.29	7.93
3qp9	4:0:0:0	:	9.57	9.82	11.78	4.78	16.19	18.27	:	5.89	7.2	8.5	3.14	13.52	15.23
3gzx	4:0:0:0	:	8.78	10.16	9.92	11.57	7.14	16.81	:	5.77	7.17	6.7	8.59	4.04	12.39
3fkf	4:0:0:0	:	9.44	6.83	8.19	12.06	17.46	11.56	:	7.99	3.49	6.29	8.47	15.9	9.27
8cm6	4:0:0:0	:	8.58	7.56	12.95	5.97	11.01	8.31	:	6	3.84	9.78	3.59	8.23	6.76
6kse	4:0:0:0	:	9.59	13.88	5.59	20.64	12.21	9.67	:	6.32	9.51	3.54	16.46	8.84	6.1
3en1	4:0:0:0	:	9.01	10.1	9.57	11.29	7.23	16.26	:	6.07	6.86	6.63	8.19	4.06	11.85
1kqf	4:0:0:0	:	6.09	6.56	12.6	10.71	16.14	11.19	:	3.58	3.76	9.98	8.08	12.99	9.38
3dfr	4:0:0:0	:	11.4	7.19	9.27	16.65	18.04	5.35	:	7.44	3.61	6.23	13.16	15.03	3.55
8zmu	4:0:0:0	:	8.73	13.02	5.28	14.56	6.57	14.59	:	7.18	8.69	3.3	12.57	3.65	10.71
3krb	4:0:0:0	:	10.62	9.53	10.48	7.74	7.18	5.78	:	8.1	7.47	8.06	3.83	3.83	3.54
7b9p	4:0:0:0	:	10.95	10.55	6.85	9.28	17.25	16.71	:	6.78	9.16	3.75	5.89	13.91	14.52
4l4x	4:0:0:0	:	10.16	9.97	11.73	4.76	16.54	18.12	:	6.86	8.23	8.28	3.43	14.06	15.78
6kob	4:0:0:0	:	10.54	6.73	8.2	9.34	10.07	9.06	:	8.18	3.93	4	6.87	7.84	6.66

6kli	4:0:0:0	:	10.92	6.88	13.72	8.41	17.31	19.41	:	8.97	3.91	9.12	6.52	14.9	15.58
3wbw	4:0:0:0	:	12.71	12.12	9.08	7.67	12.67	7.1	:	9.46	9.54	6.9	3.66	9.77	4.39
6jw6	4:0:0:0	:	7.32	11.39	6.66	14.07	5.7	11.51	:	3.7	8.91	3.59	11.08	3.6	8.68
4zcd	4:0:0:0	:	11.23	7.61	11.25	16.08	22.44	10.48	:	8.77	3.79	8.59	13.07	20.22	8.48
3d3l	4:0:0:0	:	12.4	12.36	5.57	18.08	17.86	12.01	:	9.96	8.95	3.29	15.17	14.62	8.49
2w3w	4:0:0:0	:	11.44	11.26	9.54	18.32	20.79	12.43	:	7.39	7.93	7.15	15.69	18.98	8.74
1d7b	4:0:0:0	:	7.85	12.9	11.15	9.97	17.06	15.19	:	5.08	9.93	8.15	8.93	14.05	11.8
1fft	4:0:0:0	:	8.99	7.13	8.95	13.13	11.47	9.54	:	6.97	4.26	5.71	11.04	7.96	6.95
2ipi	4:0:0:0	:	7.76	12.84	12.14	19.82	11.69	17.23	:	5.25	8.48	9.56	15.85	7.58	14.75
5c2v	4:0:0:0	:	13.44	11.7	11.77	9.94	17.59	8.49	:	9.99	7.85	8.9	8.06	15.04	6.42
6dqw	4:0:0:0	:	5.96	9.91	12.45	11.55	14.41	6.34	:	3.57	6.21	9.06	8.12	11.04	3.62
6kri	4:0:0:0	:	10.3	14.4	6.01	21.35	12.61	10.1	:	7.07	9.97	3.42	16.97	9.46	6.66
2amj	4:0:0:0	:	8.45	7.86	10.95	12.83	10.32	17.48	:	5.35	3.98	8.56	8.21	8.73	14.69
7k62	4:0:0:0	:	11.16	11.29	9.57	18.39	20.47	12.18	:	7.05	8.06	7.23	15.66	18.71	8.56
2rdz	4:0:0:0	:	7.61	12.1	6.58	11.65	11.42	10.45	:	3.79	8.88	3.68	7.88	7.08	8.17
3hlx	4:0:0:0	:	6.72	6.98	13.14	10.31	15.34	9.21	:	3.77	3.7	9.8	7.04	12.83	7.56
1gpe	4:0:0:0	:	6.58	10.6	8.89	12.82	15.12	12.2	:	3.74	9	5.36	10.09	12.42	10.39
3sdp	4:0:0:0	:	10.42	11.29	9.4	14.24	14.57	5.17	:	9.12	8.39	5.22	11.31	10.64	3.45
6ie3	4:0:0:0	:	11.75	12.39	5.27	15.43	15.98	11.83	:	8.51	8.85	3.5	13.13	12.19	8.66
6p73	4:0:0:0	:	7.58	12.18	6.48	11.7	11.38	10.68	:	3.74	8.98	3.59	8.02	7.08	8.4
3wfd	4:0:0:0	:	5.09	11.65	10.38	10.64	12.16	12.46	:	3.28	8.53	9.47	6.31	9.91	9.44
1us0	4:0:0:0	:	10.94	9.92	7.13	7.52	13.67	10.75	:	8.86	8.09	3.59	3.6	9.61	7.64
1zua	4:0:0:0	:	10.52	9.68	7.29	7.55	13.39	10.89	:	7.99	7.95	3.74	3.9	9.19	7.98
2yev	4:0:0:0	:	11.83	9.9	6.78	11	13.4	7.03	:	8.64	6.58	3.67	7.4	9.36	3.83
5ssx	4:0:0:0	:	13.43	9.43	11.77	16.03	23.24	9.49	:	9.93	7.22	9.17	13.88	20.14	8.33
5sd5	4:0:0:0	:	11.45	11.31	9.69	18.63	21.03	12.58	:	7.33	8.07	7.42	15.8	19.1	8.88
5apa	4:0:0:0	:	10.25	9.35	12.7	17.54	22.16	10.8	:	6.79	6.51	8.94	14.01	17.66	7.34
7k6c	4:0:0:0	:	11.58	11.22	9.7	18.34	21.01	12.75	:	7.49	7.98	7.38	15.65	19.35	9.09
4jo0	4:0:0:0	:	10.23	6.32	12.59	15.94	16.28	11.44	:	7.06	3.69	9.3	12.49	13.53	9.18
2nap	4:0:0:0	:	7.03	10.6	11.59	11.66	9.77	15.05	:	3.91	8.46	7.82	9.36	8.14	12.09
1lox	4:0:0:0	:	12.42	12.48	5.51	18.09	17.75	11.96	:	9.87	9.15	3.14	15.42	14.66	8.55
1wql	4:0:0:0	:	10.99	11.49	9.55	19.38	13.89	7.79	:	9.62	7.21	6.81	15.25	10.52	3.88
6s07	4:0:0:0	:	13.16	9.5	12.07	15.76	23.08	9.62	:	9.61	7.36	9.33	13.35	19.99	8.29
2cw3	4:0:0:0	:	11.98	10.35	11.25	18.29	14.99	5.86	:	9.2	8.19	7.88	15.29	11.52	3.58
3qvp	4:0:0:0	:	6.87	11.04	8.84	13.04	15.27	12.57	:	4	9.5	5.37	10.58	12.51	10.56
5zsx	4:0:0:0	:	11.7	8.64	8.67	19.12	9.22	15.77	:	9.22	5.94	6.92	15.59	6.44	12.17
6uww	4:0:0:0	:	11.55	11.6	9.55	18.43	20.98	13.05	:	7.42	8.4	7.36	15.77	19.18	9.35
5a7e	4:0:0:0	:	10.69	10.89	14.11	20.21	12.04	16.91	:	7.87	9	9.6	18.94	9.02	14.53
3s8f	4:0:0:0	:	11.16	8.2	11.78	7.84	17.7	11.2	:	7.5	5.02	9.45	4.02	14.28	8.01
3h7u	4:0:0:0	:	11	9.71	7.27	7.71	14.2	11.54	:	8.5	7.97	3.96	3.71	10.7	7.95
5z1x	4:0:0:0	:	10.85	10.89	14.03	20.31	11.83	16.86	:	7.9	8.92	9.45	19.05	8.8	14.46
3pxl	4:0:0:0	:	10.69	10.85	14.06	20.17	11.93	16.95	:	7.76	8.91	9.5	18.85	8.94	14.56
3w36	4:0:0:0	:	10.27	9.79	10.36	17.32	19.76	8.36	:	8.02	6.56	8.81	14.74	17.38	5.33
6yyx	4:0:0:0	:	10.18	9.63	12.84	17.42	22.21	10.89	:	6.71	6.88	9.08	14.01	17.77	7.45
5ehf	4:0:0:0	:	10.61	11.03	13.84	20.34	11.72	17.02	:	7.68	9.12	9.23	19.02	8.74	14.61
3kw7	4:0:0:0	:	10.31	10.96	13.46	20.2	11.85	16.89	:	7.55	9.04	8.89	19.04	8.75	14.64
7l9t	4:0:0:0	:	11.68	11.58	9.69	18.32	21.05	13.22	:	7.71	8.6	7.33	15.51	19.38	9.95
6ri6	4:0:0:0	:	10.81	10.94	13.86	20.52	12.02	17	:	8.01	9.01	9.28	19.25	8.93	14.61
2qt6	4:0:0:0	:	10.7	10.95	13.92	20.31	11.84	17.02	:	7.81	9.04	9.32	19.03	8.81	14.57
3hhd	4:0:0:0	:	11.76	11.95	11.92	9.85	18.3	10.88	:	7.81	8.06	9.4	8.06	14.46	7.37
3x1b	4:0:0:0	:	10.8	10.83	14.06	20.3	12.05	16.92	:	7.91	8.9	9.47	19.06	8.96	14.48
1gyc	4:0:0:0	:	10.76	10.99	13.99	20.42	11.95	17.02	:	7.87	9.07	9.37	19.14	8.99	14.63
1kya	4:0:0:0	:	10.67	10.78	13.91	20.22	11.91	17.04	:	7.8	8.8	9.31	18.91	8.94	14.66
1hfu	4:0:0:0	:	10.7	11.03	13.89	20.43	11.91	16.95	:	7.97	9.14	9.29	19.23	8.76	14.52
2hzh	4:0:0:0	:	10.53	10.82	13.9	20.16	11.92	17.08	:	7.65	8.81	9.3	18.89	8.89	14.66
2bii	4:0:0:0	:	12.12	5.83	10.77	13.28	16.67	7.48	:	9.38	3.57	7.44	11.17	14.4	4.42
2xyb	4:0:0:0	:	10.57	10.93	14.01	20.18	11.93	17.04	:	7.68	8.97	9.38	18.86	8.9	14.62
7u6l	4:0:0:0	:	12.59	12.34	10.48	8.24	11.67	7.35	:	9.99	9.09	7.63	5.84	10.25	4.14
3t6v	4:0:0:0	:	10.73	11.07	13.92	20.54	11.98	17.12	:	7.93	9.16	9.3	19.34	8.81	14.75
2z5y	4:0:0:0	:	12.64	9.19	12.82	6.85	18.82	13.15	:	8.6	7.28	9.28	3.7	14.84	9.81
2j7a	4:0:0:0	:	6.12	12.09	10.82	10.93	14.26	20.9	:	3.71	8.65	9.02	8.51	11.86	19.09
2nya	4:0:0:0	:	11.48	9.14	13.19	17.73	8.2	19.98	:	8.73	7.51	9.29	14.24	7.06	17.45
2vr0	4:0:0:0	:	6.15	11.9	10.89	10.91	14.37	20.8	:	3.82	8.33	9.08	8.34	12.11	18.97
3eau	4:0:0:0	:	11.19	8.23	7.29	12.51	12.91	11.51	:	8.88	5.89	3.97	9.51	11.57	7.66
1oah	4:0:0:0	:	6.14	12.17	10.65	10.79	14.26	20.73	:	3.75	8.66	8.57	8.25	11.69	18.7
2jfk	4:0:0:0	:	12.1	12.24	11.95	10.01	18.31	10.75	:	8.11	8.28	9.12	8.46	14.59	7.26
1ogy	4:0:0:0	:	11.52	8.74	13.6	17.41	7.96	19.75	:	8.33	7.14	9.73	14.04	6.81	17.47
8fw1	4:0:0:0	:	7.68	9.1	11.12	10.33	15.25	12.39	:	4.56	6.27	8.35	7.26	12.69	8.6
4udq	4:0:0:0	:	6.77	11.08	9.6	12.6	15.58	18.75	:	3.75	9.08	7.45	9.89	13.59	17.15
8j83	4:0:0:0	:	7.17	11.12	11.18	12.69	18.25	17.9	:	4.4	7.9	8.82	10.33	14.91	15.57
3ml1	4:0:0:0	:	11.66	9.1	13.66	17.58	7.88	19.76	:	8.74	7.67	9.82	14.27	6.66	17.44
6jdk	4:0:0:0	:	12.08	10.32	8.5	21.2	12.52	14.13	:	9.02	7.86	5.15	17.34	10.37	10.94
3pua	4:0:0:0	:	11.36	12.43	12.3	11.79	14.24	6.02	:	8.51	9.74	7.94	8.38	10.52	3.77
6iqx	4:0:0:0	:	10.52	11.84	11.33	5.77	14.21	13.07	:	8.01	8.34	8	3.36	11.37	9.16

4u3e	4:0:0:0	:	11.03	13.32	10.67	12.53	20.58	16.01	:	8.74	9.16	9.13	10.35	18.84	12.81
1z01	4:0:0:0	:	11.01	13.9	11.47	9.5	19.15	16.35	:	7.89	9.99	9.81	5.62	16.8	14.83
5m8s	4:0:0:0	:	10.57	10.6	7.21	11.52	13.52	14.76	:	6.81	8.41	4.7	8.53	10.43	12.36
3js8	4:0:0:0	:	11.25	12.2	9.19	9.95	11.91	15.5	:	7.46	9.64	6.2	8.14	8.88	13.5
3k3o	4:0:0:0	:	11.25	12.43	12.33	13.16	15.36	6.17	:	8.51	9.78	8	9.12	11.42	3.87
1wvf	4:0:0:0	:	11.2	11.03	7.32	15.34	16.84	14.18	:	8.37	9.36	3.44	14.22	12.57	11.02
3sqr	4:0:0:0	:	11.99	12.04	7.04	19.32	11.18	16.61	:	8.09	9.54	3.76	15.84	7.71	13.52
3kv9	4:0:0:0	:	10.81	12.41	12.24	12.98	15.32	6.73	:	7.98	9.67	7.9	9.38	11.37	4.17
6hf1	4:0:0:0	:	10.82	12.05	11.04	7.48	11.93	15.49	:	7.64	8.13	9.2	3.74	8.09	12.94
1wve	4:0:0:0	:	11.01	11.2	7.49	15.4	16.92	14.46	:	8.18	9.54	3.56	14.29	12.66	11.28
2q9o	4:0:0:0	:	5.09	10.99	13.77	13.23	13.54	16.88	:	3.34	9	9.16	10.66	10.38	14.46
1afr	4:0:0:0	:	8.02	11.66	13.23	8.79	15.6	15.08	:	4.33	9.69	9.77	5.89	12.54	12.64
2uw1	4:0:0:0	:	8	11.55	13.24	8.74	15.11	14.89	:	4.38	9.5	9.78	5.65	12.21	12.66
5nlt	4:0:0:0	:	6.69	12.98	11.59	17.14	12.4	19.08	:	4.73	9.71	9.12	13.82	10.51	15.78
7pp7	4:0:0:0	:	8.64	11.22	13.14	9.03	15.26	15.21	:	5.23	9.33	9.82	5.78	12.39	13.12
8rpg	4:0:0:0	:	12.99	10.31	12.25	17.23	23.98	14.71	:	9.71	6.99	9.06	14.61	19.63	11.83
4oua	4:0:0:0	:	10.77	11.49	13.25	11.05	11.91	16.87	:	8.92	9.19	8.98	7.49	9.11	13.66
8bbq	4:0:0:0	:	11.39	12.76	12.49	15.8	16.87	8.02	:	8.9	9.59	9.7	12.09	13.93	4.93
